# Mosaic Turner syndrome shows reduced penetrance in an adult population study

**DOI:** 10.1038/s41436-018-0271-6

**Published:** 2018-09-05

**Authors:** Marcus A. Tuke, Katherine S. Ruth, Andrew R. Wood, Robin N. Beaumont, Jessica Tyrrell, Samuel E. Jones, Hanieh Yaghootkar, Claire L. S. Turner, Mollie E. Donohoe, Antonia M. Brooke, Morag N. Collinson, Rachel M. Freathy, Michael N. Weedon, Timothy M. Frayling, Anna Murray

**Affiliations:** 1 Genetics of Complex Traits, University of Exeter Medical School, RILD Level 3, Royal Devon & Exeter Hospital, Barrack Road, Exeter, UK; 2 Peninsula Clinical Genetics, Royal Devon & Exeter Hospital, Gladstone Road, Exeter, UK; 3 Macleod Diabetes & Endocrine Centre, Royal Devon and Exeter Hospital, Exeter, UK; 4 Wessex Regional Genetics Laboratory, Salisbury NHS Foundation Trust, Salisbury District Hospital, Salisbury, UK

**Keywords:** Turner syndrome, mosaicism, aneuploidy, trisomy

## Abstract

**Purpose:**

Many women with X chromosome aneuploidy undergo lifetime clinical monitoring for possible complications. However, ascertainment of cases in the clinic may mean that the penetrance has been overestimated.

**Methods:**

We characterized the prevalence and phenotypic consequences of X chromosome aneuploidy in a population of 244,848 women over 40 years of age from UK Biobank, using single-nucleotide polymorphism (SNP) array data.

**Results:**

We detected 30 women with 45,X; 186 with mosaic 45,X/46,XX; and 110 with 47,XXX. The prevalence of nonmosaic 45,X (12/100,000) and 47,XXX (45/100,000) was lower than expected, but was higher for mosaic 45,X/46,XX (76/100,000). The characteristics of women with 45,X were consistent with the characteristics of a clinically recognized Turner syndrome phenotype, including short stature and primary amenorrhea. In contrast, women with mosaic 45,X/46,XX were less short, had a normal reproductive lifespan and birth rate, and no reported cardiovascular complications. The phenotype of women with 47,XXX included taller stature (5.3 cm; SD = 5.52 cm; *P* = 5.8 × 10^−20^) and earlier menopause age (5.12 years; SD = 5.1 years; *P* = 1.2 × 10^−14^).

**Conclusion:**

Our results suggest that the clinical management of women with 45,X/46,XX mosaicism should be minimal, particularly those identified incidentally.

## Introduction

X chromosome aneuploidy is a common chromosome abnormality.^1^ It can be difficult to determine the clinical importance of X chromosome aneuploidy because population-based studies have been limited to tens of individuals ascertained from prenatal screening and without long-term follow up into older age.^2–4^ Cohorts recruited through clinical features may be affected by ascertainment bias, because only individuals with syndromic features are being tested. It is increasingly important to determine the phenotypic consequences of genetic abnormalities in nonclinical populations as more genomic testing is carried out on individuals in the general population, including direct-to-consumer testing.^5^

Women with trisomy X (47,XXX) are reported to be taller than average and to have earlier menopause, but the evidence to support these associations is limited to potentially biased collections of cases reaching medical attention.^6^ In contrast, women with a 45,X chromosome complement or Turner syndrome are generally short (20 cm below population mean), may have physical features such as a webbed neck, and about 80% have primary amenorrhea.^7^ Women with Turner syndrome are at an increased risk of hearing difficulties and cardiac disorders, particularly dissection of the aorta.^8–11^ Hypergonadotrophic hypogonadism in Turner syndrome means that pregnancy is often difficult to achieve spontaneously, but if achieved, pregnancies are considered high risk, predominantly due to an increased risk of cardiac complications.^12^

In this study, we tested over 244,000 women from the UK Biobank, a population-based study of adults aged 40–70, for X chromosome aneuploidy using single-nucleotide polymorphism (SNP) array data and identified 326 individuals with whole X chromosome imbalances. The availability of such a large population sample allowed us to characterize the phenotypic characteristics, including adult diseases, of women with X chromosome aneuploidy in a nonclinical context.

## Materials and methods

### UK Biobank cohort

UK Biobank recruited over 500,000 individuals aged 37–73 years (99.5% were between 40 and 69 years) between 2006 and 2010 from across the United Kingdom. Participants provided a range of information via questionnaires and interviews (e.g., demographics, health status, lifestyle, anthropometric measurements).^13^ SNP genotypes were generated from the Affymetrix Axiom UK Biobank array (~450,000 individuals) and the UK BiLEVE array (~50,000 individuals). This data set underwent extensive central quality control (http://biobank.ctsu.ox.ac.uk) resulting in 244,848 females of white European descent included in subsequent analyses. Basic characteristics for these women are given in Supplemental Table S1. Our study was carried out using UK Biobank, which is covered by its own ethical review process. Details of patient/public involvement and ethical governance in the UK Biobank are available online (http://www.ukbiobank.ac.uk/about-biobank-uk/ and https://www.ukbiobank.ac.uk/wp-content/uploads/2011/07/Summary-EGF-consultation.pdf?phpMyAdmin=trmKQlYdjjnQIgJ%2CfAzikMhEnx6).

### Identification of women with chromosome X aneuploidy

**Fig. 1:**
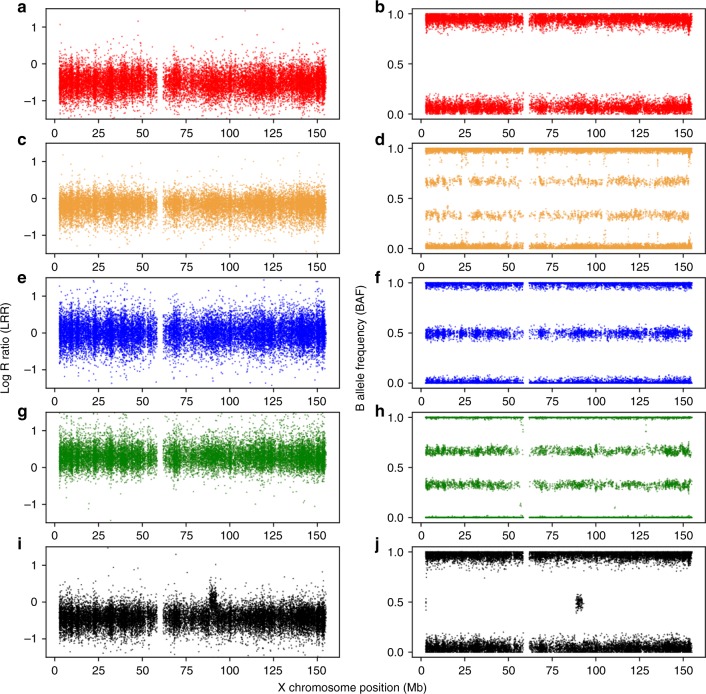
**Exemplar log R ratio (LRR) and B-allele frequency (BAF) plots for each detected X chromosome state.** (**a**,**b**) Respective LRR and BAF for a >80% dosage 45,X individual. (**c**,**d**) LRR and BAF for a mosaic (≤80%) dosage 45,X/46,XX individual respectively. (**e**,**f**) Respective LRR and BAF of a 46,XX individual. (**g**,**h**) Respective LRR and BAF of a 47,XXX individual. Finally for comparison, plots (**i**) and (**j**) represent the respective LRR and BAF of a 46,XY male illustrating a similar LRR dosage to 45,X; note the 4.5-Mb 2-copy dosage region at 88.5 Mb on chromosome X, which is homologous to the Y chromosome present in all males (PAR3)

Log R ratio (LRR) and B-allele frequency (BAF) values for 18,725 nonpseudoautosomal, chromosome X SNP probe sets were provided by UK Biobank. For each female, we calculated the mean LRR, and the number of probe sets falling inside the expected BAF heterozygous range (values between 0.49 and 0.51). We classified females as having X chromosome aneuploidy if they were outliers in both the cohort mean LRR distribution and the expected heterozygous BAF count distribution (Supplemental Fig. S1). Outliers for both mean LRR and BAF heterozygote count were defined as more than 1.5 times the interquartile range away from the mean.^14^ We visually inspected the distribution of chromosome-wide LRR and BAF to ensure aneuploidy was consistent along the full X chromosome only (Fig. 1, Supplemental Figs. S2–S4). The method was validated in a series of Turner syndrome cases tested by cytogenetics (see Supplemental Methods and Supplemental Fig. S5).

In females with chromosome X loss, the percentage of mosaicism was estimated by dividing individual mean LRR by the lowest observed mean LRR across all individuals, giving a “dosage” of 45,X cells. We defined females with chromosome X loss as mosaic 45,X/46,XX if the dosage was ≤80% and as nonmosaic 45,X if the dosage was >80%. We chose 80% mosaicism relatively arbitrarily, but it corresponded to the cytogenetic validation data (Supplementary Methods) and all but one of the individuals with hospital episode statistics (HES) records of Turner syndrome, detected by our method, had a dosage >80%. For the 186 women we classified as mosaic 45,X/46,XX, the estimated percentage of 45,X cells per sample ranged from 15 to 79%.

### Association testing for a range of phenotypes

We tested the association of X chromosome aneuploidy with a range of phenotypes and traits including anthropometric, reproductive, cardiovascular, learning/memory, and incidence of various diseases (Supplemental Table S1). X chromosome aneuploidy was stratified into three groups: 45,X nonmosaic samples, 45,X/46,XX mosaic samples, and 47,XXX samples. Controls were all other women in UK Biobank (max *N* = 244,522). We used linear regression models in STATA 13, adjusting for SNP chip type (UKB Axiom or UK BiLEVE), ancestry-principal components 1 to 5 supplied by UK Biobank, test center, and age. Hypertension and hypothyroidism were tested using logistic regression and household income was tested using ordinal logistic regression. Variables were inverse normalized to account for skewed distributions.

## Results

### Detection of chromosome X aneuploidy

We detected 326 women with whole X chromosome imbalances among the 244,848 women from the UK Biobank, 110 with a higher dosage indicating an additional X chromosome and 216 with a lower dosage, suggesting only one X chromosome in some or all cells (Fig. 1 and Supplemental Figs. S2–S4). Of the 216 suspected 45,X women there was a range of estimated dosages with mean LRRs ranging from –0.5 to –0.07 (mean = –0.23). Of these women, 30 had an estimated X chromosome dosage in the lowest quintile, suggesting the majority of cells had only one X, consistent with a 45,X karyotype. The prevalence of 45,X was therefore 12/100,000 women. Our classification of 186 women with a dosage ≤ 80% as mosaic gives a prevalence of 76/100,000 women with mosaic 45,X/46,XX in UK Biobank.

### Detection of 45,X and 45,X/46,XX mosaicism compared with hospital record data

No women self-reported having Turner syndrome at their baseline visit. There are no general practitioner (GP) records, no links to clinical genetics registries, and only in-patient hospital record (HES) data for 78% of UK Biobank participants at the time of analysis. Of the 30 women we classified with nonmosaic 45,X, 29 had HES data available, of whom 16 had an ICD-10 code for Turner syndrome (55%). This left 13 individuals who had had an overnight hospital stay, but Turner syndrome was not recorded in HES (Supplemental Fig. S6). Of the 186 women we classified as having mosaic 45,X/46,XX only one (0.5%) had a HES record of “Turner syndrome,” and she had an estimated X chromosome loss of 77.5%, close to our cutoff of 80% for nonmosaic 45,X.

In addition to the 216 women we characterized with SNP array data as 45,X nonmosaic or 45,X/46,XX mosaic, 7 women had a HES record of either “Turner syndrome” (*n* = 5) or “Mosaicism 45,X/46,XX” (*n* = 2). Of the five women with a HES record of nonmosaic “Turner syndrome,” two were not outliers for whole X chromosome dosage, but on inspection of SNP intensity and BAF data, they had a deletion of the p arm and duplication of the q arm, suggesting 46,X,i(Xq) (isochromosome Xq) (Supplemental Fig. S7); and three were not detected by the SNP array method because their SNP array metrics were well within the normal range. The two women with a HES record of “Mosaicism 45,X/46,XX” both had a normal 46,XX array profile. These samples were either below the limit of detection or could represent age-related loss of the X, which is not detected by our array method.

### Detection of partial X chromosome deletion and isochromosome Xq

After detecting that some women with HES records of “Turner syndrome” had a likely isochromosome Xq, we reanalyzed the data separately for the X chromosome p and q arms to detect additional cases with X chromosome abnormality causing Turner syndrome. We identified a total of five potential 46,X,i(Xq) cases, including the two that had a “Turner syndrome” ICD-10 code (Supplemental Fig. S8). This protocol modification also identified nine women with an X chromosome deletion of between 15 and 55 megabases in size (Supplemental Fig. S9). The 14 women with partial deletions and 46,X,i(Xq) were of shorter stature (Supplemental Table S2).

### 45,X and 45,X/46,XX mosaicism and height

**Fig. 2:**
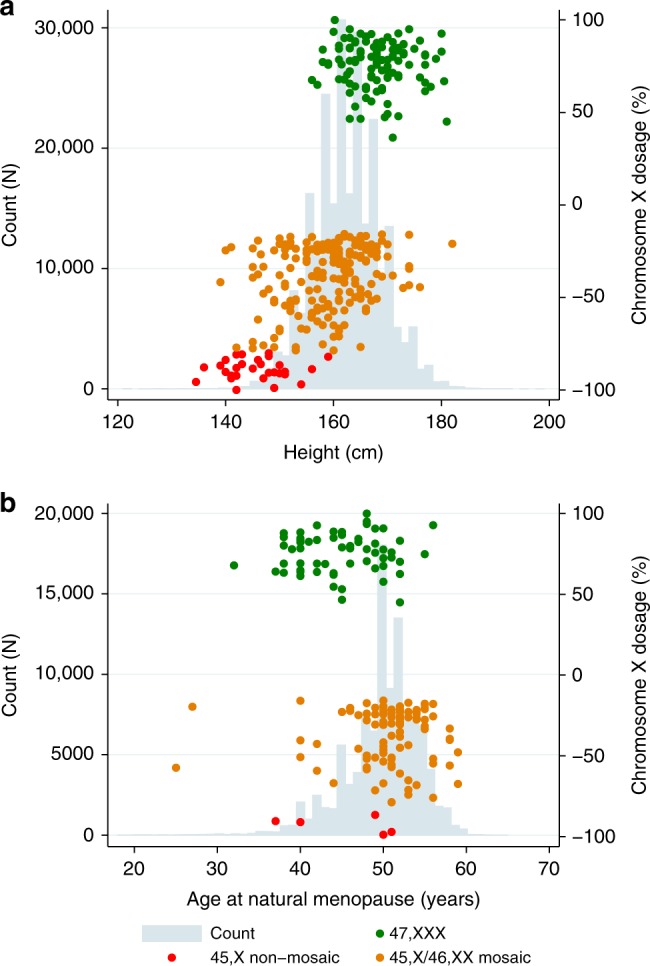
Relationship between chromosome X dosage in 45, X and 47, XXX females for two quantitative traits. (**a**) Shows the height of 45, X and 47, XXX females in UK Biobank and (**b**) Shows the natural menopause age 45, X and 47, XXX females in UK Biobank. X chromosome dosage is color coded (see key) for each category tested in the analysis. Distribution of each trait in controls is shown in gray histogram for (**a**) height and (**b**) natural menopause age

The 30 women with evidence of nonmosaic 45,X chromosome loss were on average 17.2 cm shorter than 46,XX females (Tables 1 and 2, Fig. 2, Supplemental Table S3). In contrast, the 186 mosaic 45,X/46,XX cases were on average only 3.8 cm shorter than 46,XX women (*P* = 4.0 × 10^−14^). Height ranged from 139 to 182 cm in the mosaic group, and 7% of these women were taller than one standard deviation above the mean height in 46,XX women. There was a weak correlation between percentage mosaicism and height in the women with mosaic 45,X/46,XX (*r* = 0.23).

**Table 1:** Characteristics of UK Biobank females stratified by X chromosome aneuploidy status

Trait	Status	*N* Included	*N* Missing	Mean	SD	Min. value	Max. value
Age at recruitment (years)	45,X	30	0	54.84	7.77	42	69
	45,X/46,XX	186	0	59.69	8.00	42	70
	46,XX	244,522	0	57.09	7.94	40	71
	47,XXX	110	0	56.83	7.06	41	69
Height (cm)	45,X	30	0	145.47	5.72	134.5	159
	45,X/46,XX	186	0	158.89	7.52	139	182
	46,XX	244,037	485	162.66	6.23	121	199
	47,XXX	110	0	167.98	5.52	156	181
Menarche age (years)	45,X	14	16	15.36	2.59	12	19
	45,X/46,XX	177	9	13.15	1.61	9	18
	46,XX	237,744	6778	12.96	1.61	5	25
	47,XXX	104	6	12.73	1.96	7	17
Natural menopause age (years)	45,X	5	25	45.40	6.43	37	51
	45,X/46,XX	95	91	50.58	5.35	25	59
	46,XX	108,479	136,043	50.02	4.53	18	65
	47,XXX	60	50	44.90	5.10	32	56
Number of pregnancies	45,X	29	1	0.24	0.64	0	2
	45,X/46,XX	186	0	2.22	1.75	0	10
	46,XX	240,046	4476	2.30	1.58	0	31
	47,XXX	107	3	1.93	1.77	0	10
Fluid intelligence score (0–13)	45,X	11	19	5.82	1.94	3	9
	45,X/46,XX	79	107	5.99	2.32	2	12
	46,XX	118,776	125,746	5.80	2.04	0	13
	47,XXX	51	59	4.39	1.72	1	8
Household income category (1–5)	45,X	21	9	2.24	1.18	1	5
	45,X/46,XX	156	30	2.28	1.17	1	5
	46,XX	203,115	41,407	2.53	1.18	1	5
	47,XXX	78	32	1.50	0.75	1	4
Townsend deprivation index	45,X	30	0	−1.32	2.54	−6.11	5.70
	45,X/46,XX	186	0	−1.26	2.89	−5.39	6.71
	46,XX	244,238	284	−1.51	2.93	−6.26	11.00
	47,XXX	110	0	−0.54	3.38	−5.95	6.81
Birth weight (kg)	45,X	14	9	3.07	0.28	2.72	3.40
	45,X/46,XX	89	77	3.36	0.40	2.55	4.28
	46,XX	130,961	92,844	3.35	0.42	2.5	4.5
	47,XXX	56	54	3.27	0.45	2.55	4.37
Body mass index (kg/m^2^)	45,X	30	0	27.19	4.30	18.36	35.94
	45,X/46,XX	185	1	26.70	4.97	18.64	46.25
	46,XX	243,502	1020	27.02	5.14	12.12	74.68
	47,XXX	110	0	29.18	5.48	17.86	46.48

“45,X” are individuals assumed to be nonmosaic Turner syndrome individuals. “45,X/46,XX” are individuals assumed to be mosaic Turner syndrome. “46,XX” are controls, and “47,XXX” are trisomy X individuals. Sixteen and 25 nonmosaic 45,X cases did not report menarche or menopause ages respectively

**Table 2:** Association between X chromosome aneuploidy and nine phenotypes

Trait	Status	*N*	Effect (SD)	Low 95% CI (SD)	High 95% CI (SD)	*P*
Height (cm)	45,X	30	−2.73	−3.07	−2.38	**1.5** **×** **10** ^**−53**^
	45,X/46,XX	186	−0.54	−0.68	−0.40	**4.0** **×** **10** ^**−14**^
	47,XXX	110	0.84	0.66	1.03	**5.8** **×** **10** ^**−20**^
Menarche age (years)	45,X	14	1.33	0.81	1.86	**5.9** **×** **10** ^**−7**^
	45,X/46,XX	177	0.12	−0.03	0.26	0.12
	47,XXX	104	−0.15	−0.34	0.05	0.14
Natural menopause age (years)	45,X	5	−0.99	−1.86	−0.13	0.024
	45,X/46,XX	95	0.12	−0.08	0.32	0.23
	47,XXX	60	−0.98	−1.23	−0.73	**1.2** **×** **10** ^**−14**^
Number of pregnancies	45,X	29	−1.50	−1.86	−1.14	**3.3** **×** **10** ^**−16**^
	45,X/46,XX	186	−0.12	−0.27	0.02	0.09
	47,XXX	107	−0.26	−0.44	−0.07	0.0076
Fluid intelligence score (0–13)	45,X	11	−0.17	−0.74	0.39	0.55
	45,X/46,XX	79	0.09	−0.12	0.30	0.39
	47,XXX	51	−0.74	−1.00	−0.48	**3.7** **×** **10** ^**−8**^
Household income category (1–5)	45,X	21	−0.89	−1.66	−0.12	0.024
	45,X/46,XX	156	−0.21	−0.51	0.08	0.15
	47,XXX	78	−1.98	−2.45	−1.51	**8.7** **×** **10** ^**−17**^
Townsend deprivation index	45,X	30	0.13	−0.20	0.47	0.43
	45,X/46,XX	186	0.13	0.00	0.27	0.052
	47,XXX	110	0.24	0.07	0.42	0.0067
Birth weight (kg)	45,X	13	−0.67	−1.19	−0.14	0.013
	45,X/46,XX	89	0.02	−0.19	0.23	0.86
	47,XXX	56	−0.21	−0.47	0.05	0.11
Body mass index (kg/m^2^)	45,X	30	0.13	−0.26	0.51	0.51
	45,X/46,XX	185	−0.09	−0.25	0.06	0.23
	47,XXX	110	0.46	0.26	0.66	**6.3** **×** **10** ^**−6**^

*CI* confidence interval.

The effect of the aneuploidy on each trait is given in number of standard deviations change compared with 46,XX women, with an association *P* value. *P* values that pass the threshold for statistical confidence, corrected for multiple testing, are highlighted (i.e., equivalent to *P* < 0.05)

### 45,X and 45,X/46,XX mosaicism and ovarian function

Primary amenorrhea was not specifically coded in UK Biobank, but 16/30 women with nonmosaic 45,X answered “don’t know” or “prefer not to answer” to the question “How old were you when your periods started?”. This proportion (53%) was much higher than that in 46,XX women (2.9%) and we assume most of these women did not go through menarche. For the other 14 women with nonmosaic 45,X, menarche ages between 12 and 19 years were recorded. In contrast, only 9 of the 186 women with mosaic 45,X/46,XX did not report an age at menarche, and the mean menarche timing was 13.2 years in those 177 individuals who recorded an age, not different from the mean age of 12.95 years in the 46,XX women (*P* = 0.12).

The recruitment ages of the 30 women with nonmosaic 45,X ranged from 42 to 69 years, but only 5 reported a natural menopause age and the odds ratio (OR) for not reporting an age at natural menopause compared with women with 46,XX was 3.98 (95% confidence interval [CI] = 1.50, 13.31; *P* *=* 0.0026). Ninety-five of the 45,X/46,XX mosaic women reported an age at natural menopause (Tables 1 and 2, Fig. 2, Supplemental Table S3), at an average age of 50.6 years (range = 25–59 years), again not different from the mean age in control women with 46,XX (mean = 50 years; range = 18–65; *P* = 0.23).

Only 4 of the 30 nonmosaic 45,X cases had ever been pregnant, much fewer than in the remaining 46,XX women with an odds ratio for never being pregnant of 32.35 (95% CI = 11.21, 127.22; *P* = 8.98 × 10^−17^). Most of the mosaic 45,X/46,XX cases reported a pregnancy, with only 37 of 186 reporting they had never been pregnant, a frequency not different from women with 46,XX. The mean number of pregnancies in the 45,X/46,XX women was 2.2, not different from the control 46,XX individuals (*P* *=* 0.09). There was also no increased incidence of pregnancy loss in the women with 45,X/46,XX, either from miscarriage, termination, or stillbirth.

### 45,X and 45,X/46,XX mosaicism and cardiac function

Heart defects are a common reported feature of Turner syndrome, but we found few cases of heart defects in 45,X women, based on self-report and ICD-10 codes. Four of the 30 women with 45,X had a medically diagnosed heart condition, including coarctation/dissection of the aorta and myocardial infarction. Blood pressure and hypertension risk were elevated (e.g., hypertension OR = 3.15; 95% CI = 1.41, 6.99; *P* *=* 0.005), and arterial elasticity was 1.05 indices lower in the 30 nonmosaic 45,X women (se = 0.32; *P* = 0.001) and 13 were on blood pressure medication (OR = 3.66; 95% CI = 0.93, 32.08; *P* = 7.7 × 10^−4^). Hypertension and blood pressure were not raised in the mosaic 45,X/46,XX women (hypertension OR = 1.02; 95% CI = 0.75, 1.40; *P* = 0.88) (Table 3), The women with mosaic 45,X/46,XX had not had more cardiac operations and were not more likely to be on blood pressure medication than the 46,XX women.

**Table 3:** Arterial stiffness, blood pressure, deafness, heel bone mineral density, hypothyroidism, and hypertension in women with X chromosome aneuploidy compared with women with 46,XX karyotype

Phenotype	Status	*N* ^a^	Mean	SD	Min. value	Max. value	Effect in SD (se)^b^	*P*
Pulse wave	45,X	10	6.31	2.55	3.1	10.7	−1.02 (0.32)	0.001
Arterial	45,X/46,XX	57	9.14	3.57	4.8	18.8	0.02 (0.13)	0.9
Stiffness index	46,XX	78,897	8.81	3.93	1	530		
Diastolic blood pressure (mm/Hg)	45,X	30	89.10	14.83	69	117	0.52 (0.17)	0.002
	45,X/46,XX	186	83.95	13.93	50.5	126	−0.04 (0.07)	0.55
	46,XX	243,408	84.08	13.23	44.5	147		
Systolic blood pressure (mm/Hg)	45,X	30	150.45	25.65	109	203	0.50 (0.16)	0.003
	45,X/46,XX	186	143.61	26.36	86	232	−0.04 (0.07)	0.52
	46,XX	243,858	140.59	24.28	72	252.5		
Hypertension (yes/no)	45,X	10/20					3.15 (1.41,6.99)	0.005
	45,X/46,XX	86/99					1.02 (0.75,1.40)	0.88
	46,XX	128,474/114,539						
Left ear speech reception threshold (SNR)	45,X	9	−4.78	2.67	−9	−0.5	0.84 (0.31)	0.0075
	45,X/46,XX	54	−6.44	1.88	−9	−1	0.04 (0.13)	0.75
	46,XX	76,887	−6.71	1.90	−11.25	8		
Right ear speech reception threshold (SNR)	45,X	9	−4.83	1.80	−7	−2	0.92 (0.31)	0.0034
	45,X/46,XX	55	−5.92	2.63	−9.5	5.5	0.22 (0.13)	0.083
	46,XX	76,816	−6.67	1.92	−11.5	8		
Wears a hearing aid (yes/no)	45,X	10/13					35.43 (14.79,84.91)	1.2 × 10^−15^
	45,X/46,XX	101/15					2.54 (1.45,4.43)	0.0011
	46,XX	132,646/6,129						
Reported hearing problems (yes/no)	45,X	11/17					6.54 (3.03,14.10)	1.7 × 10^−6^
	45,X/46,XX	118/61					1.74 (1.27,2.38)	5.1 × 10^−4^
	46,XX	183,121/50,393						
Heel bone mineral density (g/cm^2^)	45,X	30	0.45	0.12	0.23	0.70	**−**0.63 (0.17)	2.3 × 10^−4^
	45,X/46,XX	183	0.50	0.12	0.27	0.97	**−**0.10 (0.07)	0.14
	46,XX	240,063	0.52	0.12	0.00	1.82		
Hypothyroidism (yes/no)	45,X	20/10					6.61 (3.07,14.23)	1.4 × 10^−6^
	45,X/46,XX	166/20					1.31 (0.82,2.08)	0.26
	46,XX	225,349/19,173						

The effect of the aneuploidy on each trait is given in standard deviations compared with 46,XX women with an association *P* value. Signal-To-Noise Ratio values are abbreviated as 'SNR'.

^a^*N* shows the number of individuals within the group except for binary categorical traits hypothyroidism and hypertension, which give *N*-controls/*N*-cases.

^b^Effects given in SD (se) except for yes/no traits (logistic regression), which are given as odds ratio (OR) with 95% confidence intervals in parentheses

### Trisomy X phenotype

Trisomy X was detected in 110 women, giving a prevalence of 45/100,000 in UK Biobank women. A further two cases appeared to be mosaic 46,XX/47,XXX, but were excluded from further analyses because there were too few in this category to analyze separately. Of the 110 47,XXX women, 90 were present in the HES records, but none had a prior diagnosis of “Karyotype 47,XXX” as determined by ICD-10 code.

We found an association between trisomy X and adult height (ß = 0.84 SDs; 95% CI = 0.66, 1.03; *P* = 5.8 × 10^−20^). Women with 47,XXX were 5.3 cm taller on average than 46,XX women (Fig. 2, Tables 1 and 2, Supplemental Table S3). Women with trisomy X had normal age at menarche, but an average 5.12 years earlier age of natural menopause (ß = -0.98 SDs; 95% CI = -1.23, -–0.73; *P* = 1.2 × 10^−14^) (Tables 1 and 2, Fig. 2, Supplemental Table S3). Women with 47,XXX had a similar number of pregnancies (mean = 1.9, range 0–10) and no higher frequency of pregnancy loss than 46,XX controls. We also found that women with trisomy X had a lower fluid intelligence (ß = –0.74 SDs; 95% CI = –1.0, –0.48; *P* *=* 3.7 × 10^−8^) and a decreased household income (ß = –1.98 SDs; 95% CI = –2.45, –1.51; *P* = 8.7 × 10^−17^) than 46,XX women (Tables 1 and 2, Supplemental Table S3). Sixty-three percent of women in the 47,XXX group had a household income less than £18,000 per year, compared with 24% of 46,XX control women. Women with 47,XXX were more likely to live alone (OR = 2.07; 95% CI = 1.36, 3.12; *P* *=* 7 × 10^−4^) with 35.2% (37/110) living alone compared with 19.8% of 46,XX women. The women with 47,XXX also had a higher body mass index (BMI) than the 46,XX women, by on average over 2 BMI units (ß = 0.46 SDs; 95% CI = 0.26, 0.66; *P* *=* 6.3 × 10^−6^).

## Discussion

The availability of more than 244,000 adult women with SNP array data in a population-based study enabled us to assess X chromosome aneuploidy phenotypes without the potential ascertainment biases of clinical presentation and with sufficient statistical power to quantify phenotypic effects, including adult-onset diseases. Previous studies have either concentrated on clinically ascertained women, or prenatally ascertained cases numbering in the tens and not followed up into adulthood. While these studies have provided fundamental clinical descriptions of Turner and XXX syndrome, they have not focused on the penetrance of the genetic anomalies in the general population throughout the life course. We identified 326 women in UK Biobank with whole X chromosome dosage anomalies: 216 consistent with having a 45,X or 45,X/46,XX mosaic karyotype and 110 with 47,XXX.

### Prevalence of X chromosome aneuploidy

We detected a 45,X or 45,X/46,XX mosaic karyotype in 88/100,000 women in UK Biobank. The published prevalence of Turner syndrome is 40/100,000, but only 60% of those Turner syndrome cases are caused by 45,X or 45,X/46,XX mosaicism, the rest being due to other abnormalities such as deletions or isoXq,^15, 16^ thus the prevalence of aneuploidy causing Turner syndrome is around 24/100,000. This is nearly four times lower than the prevalence we detected in UK Biobank, suggesting that many cases go undiagnosed. Most series report approximately 16% of aneuploidy cases being mosaic 45,X/46,XX, but in UK Biobank the proportion was 86%. The prevalence of 45,X was only 12/100,000, lower than the expected prevalence of approximately 20/100,000. This difference is likely to be due in part to the UK Biobank favoring healthy individuals, but also we may have classified some 45,X cases as mosaic.

The prevalence of 47,XXX detected by SNP array in UK Biobank was 45/100,000, substantially lower than the reported incidence of 100/100,000 live births. We observed an association with IQ-related measures in women with 47,XXX, and it is known that individuals with lower IQ were less likely to participate in the study.^17^

### Most women with 45,X/46,XX mosaicism detected incidentally will not require clinical follow up

The most important implication of our data is that many women with a 45,X cell line likely do not require currently recommended interventions.^18^ The international clinical practice guidelines do not readily differentiate mosaic from nonmosaic 45,X, yet our data suggest that level of mosaicism is an important clinical indicator of Turner syndrome features and complications.^18^ In UK Biobank there were 186 women who were mosaic for X chromosome loss. Surprisingly, the phenotype in these women was unremarkable: while they were slightly shorter on average, many of the women in this category were of average height, with the tallest individual being 182 cm, despite 20% of blood cells being 45,X (Supplemental Fig. S3ah). This group of women went through menarche and menopause at an average age, had an average number of children, were not at increased risk of pregnancy loss, and there was no evidence of increased risk of cardiac complications. It is likely that women with mosaic 45,X/46,XX seen in clinical practice are a more severe subgroup of the population-wide group of women with this chromosome anomaly.

Currently any women identified as having a 45,X cell line not thought to be due to age-related loss, would be diagnosed as having Turner syndrome and would be offered extensive monitoring, particularly during pregnancy.^18^ While it is recognized that mosaic cases may be less likely to have abnormalities than nonmosaic 45,X Turner syndrome cases they are still considered high risk with respect to cardiac anomalies and hypertension during pregnancy for instance. In young women, one of the main concerns will be the likelihood of infertility associated with a diagnosis of Turner syndrome. Our data suggest that in fact the presence of a 45,X cell line in blood does not adversely affect reproductive lifespan or fertility in most cases, as long as more than 20% of cells have two X chromosomes.

### Of the 326 women in UK Biobank with X chromosome aneuploidy, 30 had a complete loss of one X chromosome, and their features were consistent with a diagnosis of Turner syndrome

Women with 45,X had a phenotype that was characteristic of that reported for this chromosomal abnormality, i.e., short stature and primary amenorrhea being the main features. Cardiac abnormalities were not very common in this group, but one had coronary artery disease and three had “heart valve disease” and/or “stricture of the artery,” confirming the need for continued screening programs. There were no data available on physical characteristics, such as neck webbing, which is a common physical feature in Turner syndrome.

### Women with 47,XXX are taller, have earlier menopause, and are of below average cognitive ability

These findings are important because they suggest that ascertainment bias has not dramatically influenced the current consensus regarding the 47,XXX phenotype. Traditionally 47,XXX syndrome has been identified by cytogenetic testing, which is costly, labor intensive, and requires fresh tissue to culture dividing cells. Thus the majority of studies on 47,XXX have been on women tested for a clinical presentation such as learning difficulties, or identified through prenatal testing as an incidental finding. Cohorts of older women with 47,XXX are therefore uncommon. Primary ovarian insufficiency (POI) is often a reason for referral for 47,XXX testing.^19^ Our data suggest that there is a genuine effect on reproductive lifespan in women with 47,XXX. This effect appears to be limited to the end of reproductive life however, as these women had average menarche age, but went through menopause on average more than 5 years earlier than women with 46,XX, with 10 women meeting the criteria for POI, with menopause before 40 years. The frequency of POI in 47,XXX women is therefore 9%, approximately ten times that seen in 46,XX women. We found no evidence for an impact of 47,XXX on reproductive function throughout life, as these women had an average number of pregnancies and no significant increase in pregnancy loss.

The UK Biobank women with 47,XXX were on average 5.3 cm taller than the 46,XX women. Adult height is associated with puberty timing, with earlier puberty resulting in shorter adult height. The 47,XXX effect on adult height is however unlikely to be driven by later puberty timing, as menarche age was not significantly different from the 46,XX women. It is more likely that the increase in height is caused by dosage-sensitive genes responsible for postpubertal growth.

The IQ of girls with 47,XXX is reported to be within the normal range, but approximately 10–15 points below their sibling average. Lower IQ is also reported in adult cases of 47,XXX, along with psychosocial features, such as low self-esteem, language difficulties, and increased prevalence of psychiatric disorders.^20^ Few series of older women with 47,XXX have been reported previously, but a study from Denmark found reduced socioeconomic status in a series of 108 women with 47,XXX.^21^ In the UK Biobank study we found a substantial reduction in fluid intelligence compared with the 46,XX women. “Fluid intelligence” describes the capacity to solve problems that require logic and reasoning ability, independent of acquired knowledge. We observed an increase in BMI in women with 47,XXX, that is not reported as a typical feature of this chromosome abnormality, and may be related to the observed lower IQ. There was also a reduction in household income in women with 47,XXX. The association with lower household income was not a consequence of general lower socioeconomic status because Townsend deprivation index was not significantly associated with 47,XXX status. Ability to work and type of employment taken by younger women with 47,XXX has been reported in other studies, but with much smaller sample sizes. Thus the reduced IQ observed in adolescents with 47,XXX continues into older age and appear to have a lifelong effect on income.

### Limitations

The samples tested in this study were from peripheral blood and may not reflect the level present in brain, ovary, or heart, for example. Nonaneuploid causes of Turner syndrome were not included in our analyses, although we detected cases of apparent isoXq, and X chromosome deletions, but we were unable to verify these cytogenetically. Any cases with Y chromosome material were excluded as sex mismatches.

Our study is biased in favor of healthier individuals with a more benign phenotype.^17^ Biobank volunteers are more likely to be female, have higher IQ, and be from a higher socioeconomic group than the general population.^17^ Furthermore cases with a more severe phenotype that results in early death will not be represented in this data set. Cases of X chromosome aneuploidy with a severe phenotype may be underrepresented in the Biobank cohort, but our study goes a long way toward redressing the bias seen in many published series of cases ascertained through clinical referrals .^22^

### Validation

While we were not able to karyotype any of the samples from UK Biobank, we did use two array platforms to test an independent series of patients with various levels of 45,X mosaicism and found close correlation between the methods. Further validation came from finding 16 previously known Turner syndrome cases in UK Biobank among the 30 individuals who we classified as nonmosaic 45,X with the array dosage data. Three previously diagnosed cases of Turner syndrome had a normal 46,XX SNP array profile; this may be a sample mix-up, or a clinical case not confirmed by genetic testing, or a more complex genetic abnormality not detected by the array method. Indeed two additional previously known Turner syndrome cases were suspected to be 46X,i(Xq), which contribute to about 15% of published cases.^15, 23^ We adapted our protocol to find additional nonaneuploid X chromosome abnormalities and found a total of five suspected 46X,i(Xq) and nine with X chromosome deletions, although we were not able to validate with an independent technology. We are confident that the cases we have identified do not include false positives, but there may be X chromosome imbalances that we did not detect.

### Conclusions

We have assessed X chromosome aneuploidy phenotypes into adulthood, without the ascertainment biases of clinical presentation. X chromosome aneuploidy was not always associated with an adverse phenotype: 45,X/46,XX mosaic females had a normal reproductive lifespan and birth rate, with no reported cardiovascular complications. This study will inform the review of guidelines on the future management of women with a 45,X/46,XX mosaic karyotype, particularly as more individuals are obtaining genomic information either as part of health-care assessments or through direct-to-consumer genetic testing.

## Electronic supplementary material

Supplementary information
