# Supplementary material for: Mosaic Turner syndrome shows reduced penetrance in an adult population study
Source: Genet Med. 2018 Sep 5;21(4):877–86. doi: 10.1038/s41436-018-0271-6 (PMC6752315; doi:10.1038/s41436-018-0271-6)
Supplement: Supplementary file 1 — Supplementary information [file 41436_2018_271_MOESM1_ESM.docx]

**Supplemental Information**

**Funding Information**

**Supplemental Methods.** SNP array analysis in UK Biobank and validation of 45,X mosaicism

**Supplemental Results.** Additional disorders in women with X chromosome aneuploidy

**Supplemental Figure S1.** Histograms representing the population-level data used to determine X chromosome aneuploidy outliers. In figure (**a**) each data point in the histogram represents the mean Log R Ratio (LRR) across chromosome X for a UK Biobank female, dotted red lines are the cut-offs used for mean LRR outliers (cut-off values: lower=-0.078, upper=0.087) and for (**b**) each data point in the histogram represents the number of heterozygote B Allele Frequencies (BAF) ranging from 0.49 to 0.51 with the lower red dotted line being the cut-off for BAF heterozygote count outliers (cut-off value=521). Females defined as outliers in both mean LRR and BAF heterozygote count were labelled as having X chromosome aneuploidy

**Supplemental Figure S2.** Plots (**a-ad**) represent Log R Ratio (LRR) and B Allele Frequency (BAF) in 30 samples that were identified as being full 45,X individuals (> 80% 45,X)

**Supplemental Figure S3.** Plots (**a-gd**) represent Log R Ratio (LRR) and B Allele Frequency (BAF) in 186 samples that were identified as being 45,X/46,XX mosaic individuals (between 15% and 80% 45,X)

**Supplemental Figure S4.** Plots (**a-df**) represent Log R Ratio (LRR) and B Allele Frequency (BAF) in 110 samples that were identified as being full 47,XXX individuals

**Supplemental Figure S5.** Log R Ratio (LRR) and B Allele Frequency (BAF) plots for each of the validation samples tested by conventional cytogenetics and compared with the SNP array method using two technologies: the Affymetrix UK Biobank Axiom array (**a, c, e, g, i and k**), and the Illumina Infinium HTS Global Screening array (**b, d, f, h, j and l**). Figures (**a**) and (**b**) represent LRR/BAF of a 45,X sample confirmed in both cytogenetics and both SNP arrays, (**c**) confirms the 11% 45,X/46,XX mosaicism in cytogenetic testing on the Axiom SNP array but appears 46,XX in the Illumina SNP array in (**d**), (**e**) and (**f**) represent LRR/BAF of a 20-25% 45,X/46,XX mosaic on both SNP arrays and was 26% 45,X/46,XX mosaic in cytogenetic testing, (**g**) and (**h**) were 46,XX in both SNP arrays confirming cytogenetic testing, (**i**) and (**j**) were 30% 45,X/46,XX on the SNP arrays but 13% 45,X/46,XX in cytogenetic testing, LRR suggested 67% mosaicism in (**k**), but BAF was not consistent across the whole X and would have been removed from our analysis, but (**i**) confirmed 50% 45,X/46,XX mosaicism in cytogenetic testing on the Illumina SNP array

**Supplemental Figure S7.** Plots (**a-g**) showing the remaining seven cases with a prior diagnosis of Turner syndrome not detected in our analysis. Two (**a and b**) were reported as ‘Turner mosaics’ but had a normal 46,XX SNP array profile, three (**c, e and g**) were reported as 45,X but had a normal 46,XX SNP array profile and two (**d and f**) had a deletion of the p-arm and duplication of the q-arm, suggesting isochromosome Xq

**Supplemental Figure S6.** The relationship between the 30 individuals detected as having full 45,X (indicated as blue circles) in our data and a total of 24 individuals with a ‘Q96 Turner Syndrome’ ICD-10 code (indicated as green circles). An explanation is given for the individuals with ICD-10 codes that were not identified as 45,X by our analyses

**Supplemental Figure S8.** Plots (**a-e**) represent Log R Ratio (LRR) and B Allele Frequency (BAF) in 5 individuals that were estimated as being 46,X,i(Xq) when analysing the Xp and Xq arms separately. In each case the karyotype is indicated by the presence of a single copy of Xp and three copies of Xq

**Supplemental Figure S9.** Plots (**a-i**) represent Log R Ratio (LRR) and B Allele Frequency (BAF) in 9 individuals that had significantly large deletions when analysing Xp and Xq arms separately. The deletions range from 15Mb to 55Mb in size. Plot (**b**) is the only sample with a deletion of any part of Xq in this size range, all other deletions were on Xp

**Supplemental Table S1.** Summary of demographic characteristics of 245,203 female participants of white European ancestry with valid genetic data. Characteristics are stratified by socioeconomic, anthropometric, cognitive, health and reproductive measures where available

**Supplemental Table S2.** Association of 46,X,i(Xq) and 46,XX,del(X) with inverse normalised height

**Supplemental Table S3.** Association between X chromosome ploidy and nine untransformed phenotypes. The effect of the aneuploidy on each trait is given in SD units compared with 46,XX women

**Supplemental Table S4.** Degree of X chromosome loss in samples tested by conventional cytogenetics, compared to SNP arrays

**Funding Information.** A.R.W. and T.M.F. are supported by the European Research Council grant: 323195:GLUCOSEGENES-FP7-IDEAS-ERC. R.M.F. is a Sir Henry Dale Fellow (Wellcome Trust and Royal Society grant: 104150/Z/14/Z). H.Y. is an R D Lawrence Fellow, funded by Diabetes UK. R.B. is funded by the Wellcome Trust and Royal Society grant: 104150/Z/14/Z. J.T. is funded by the ERDF and a Diabetes Research and Wellness Foundation Fellowship. S.E.J. is funded by the Medical Research Council (grant: MR/M005070/1). M.A.T., M.N.W. and A.M. are supported by the Wellcome Trust Institutional Strategic Support Award (WT097835MF). (323195). We thank the High-Throughput Genomics Group at the Wellcome Trust Centre for Human Genetics (funded by Wellcome Trust grant reference 090532/Z/09/Z) for the generation of the array data for validation. The funders had no influence on study design, data collection and analysis, decision to publish, or preparation of the manuscript.

**Supplemental Methods**

**SNP array analysis in UK Biobank**

Principal components were generated in the 1000 Genomes Cohort (phase 3) using high-confidence SNPs to obtain their individual loadings. These loadings were then used to project all of the UK Biobank samples into the same principal component space and individuals were then clustered using principal components 1 to 4. We removed 7 participants who withdrew from the study, and 348 individuals whose self-reported sex did not match their genetic sex based on relative intensities of X and Y chromosome SNP probe intensity.

**Calculation of LRR and BAF from SNP intensity data**

The UK Biobank used Affymetrix Power Tools software to generate LRR values based on SNP genotype clustering; the combined probe intensity for both alleles at each SNP were compared to the population mean (log_2_(R_obs_/R_mean_)) where R is the intensity of the combined alleles at each given probe. The UK Biobank workflow also generated BAF values representing the proportion of the combined SNP allele intensity accounted for by each allele. Further information on how the SNP probe intensity data were derived can be found in the UK Biobank genotyping and quality control document (http://www.ukbiobank.ac.uk/scientists-3/genetic-data/).

**Validation of 45,X mosaicism**

To validate the use of SNP genotype dosage for determining 45,X mosaicism, we tested six lymphocyte DNA samples from an independent source. The DNA was from individuals tested at the Wessex Regional Genetics Laboratory with a range of 45,X mosaicism, as determined by traditional karyotyping. The samples included a non-mosaic 45,X sample, a 46,XX sample and four 45,X/46,XX mosaics. The six samples were genotyped using both the Illumina Infinium HTS assay on Global Screening array, and the Affymetrix Axiom UK Biobank array.

Processing and derivation of LRR and BAF were carried out using Illumina Genome Studio for the Illumina array data. PennCNV-Affy was used to normalise and derive LRR/BAF values from the raw probe intensity data from the Affymetrix array together with 1,000 randomly selected UK Biobank samples. The percentage dosage was derived for Illumina and Affymetrix data separately by dividing mean LRR by the smallest detected mean LRR value amongst the 6 samples. All analysis was carried out blinded to the results of the karyotyping analysis. We checked for concordance between the independent karyotyping results and mean LRR across chromosome X and then visually inspected the LRR/BAF plots (**Supplemental Fig. S5**).

There was high level of concordance between the arrays and cytogenetic estimates, with all samples differing by <10% (**Supplemental Table S4**). A perfect correlation between the two methods may be unrealistic as cytogenetic analysis is carried out on cultured lymphocytes, rather than whole blood and thus could be subject to clonal expansion of one cell population over the other. We predicted that age-related loss of the X chromosome would not be detected by the SNP array methodology. As age-related loss is a random process, with a different X being lost in each cell, the BAF pattern would not differ from 46,XX women and the change in dosage as measured by LRR would be relatively small and beyond the limit of detection by our method. However, the SNP array method can detect 45,X mosaics representing a clonal 45,X cell line as the same X chromosome will be absent in each 45,X cell. In summary we were able to verify that the SNP array methodology was able to accurately call 45,X genetic abnormalities down to about 15% 45,X cells, but not age-related loss of the X.

A range of phenotypes were available in UK Biobank, derived from self-reported questionnaire data, ICD-10 diagnoses recorded in Hospital Episodes Statistics (HES) data and measurements taken at baseline clinic visits as part of the study. ICD-10 diagnoses are added to HES if an individual is admitted to hospital as an inpatient. Seventy-eight percent of UK Biobank individuals had a HES record with one or more ICD-10 codes.

**Supplemental Results**

**Additional disorders in women with X chromosome aneuploidy**

We detected several other conditions in the women with non-mosaic 45,X, but few in the women with mosaic 45,X/46,XX. There was an increased prevalence of diagnosed hypothyroidism in the non-mosaic 45,X women compared with 46,XX women (OR = 6.61; 95% CI = 3.07, 14.23; P = 1.4 x 10^-6^). Heel bone mineral density was slightly reduced in the non-mosaic 45,X women compared to 46,XX women (ß = -0.63 SDs; se = 0.17 SDs; P = 2.3 x 10^-4^). There was an increased incidence of hearing abnormalities in the non-mosaic 45,X women, with more women wearing a hearing aid (OR = 35.43; 95% CI = 14.79, 84.91; P = 1.2 x 10^-15^), reporting ‘hearing problems’ (OR = 6.54; 95% CI = 3.03, 14.10; P = 1.7 x 10^-6^) and having poorer average measured hearing than 46,XX women (ß = 0.88 SDs; se = 0.31 SDs; P = 0.0055) (**Table 3**). In contrast there was no increased prevalence of diagnosed hypothyroidism in the mosaic 45,X/46,XX women (OR = 1.31; 95% CI = 0.82, 2.08; P = 0.26) and bone mineral density was similar to that of 46,XX women (**Table 3**). We did observe hearing problems in the mosaic 45,X/46,XX women, but with lower incidence and effect sizes. For example, there were more 45,X/46,XX women wearing a hearing aid (OR = 2.54; 95% CI = 1.45, 4.43; P = 1.1 x 10^-3^) or reporting ‘hearing problems’ (OR = 1.74; 95% CI = 1.27, 2.38; P = 5.1 x 10^-4^) than 46,XX women, but no difference in measured hearing (P > 0.05) (**Table 3**).

**Supplemental Figure S1.** Histograms representing the population-level data used to determine X chromosome aneuploidy outliers. In figure (**a**) each data point in the histogram represents the mean Log R Ratio (LRR) across chromosome X for a UK Biobank female, dotted red lines are the cut-offs used for mean LRR outliers (cut-off values: lower=-0.078, upper=0.087) and for (**b**) each data point in the histogram represents the number of heterozygote B Allele Frequencies (BAF) ranging from 0.49 to 0.51 with the lower red dotted line being the cut-off for BAF heterozygote count outliers (cut-off value=521). Females defined as outliers in both mean LRR and BAF heterozygote count were labelled as having X chromosome aneuploidy


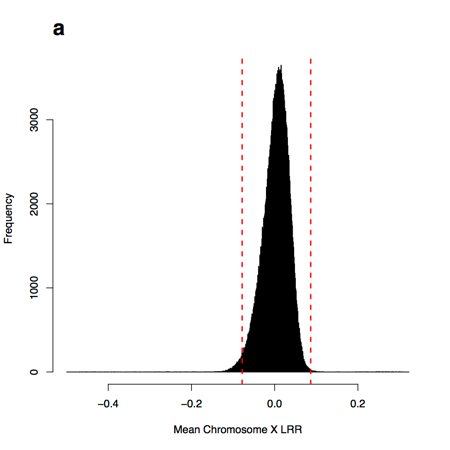

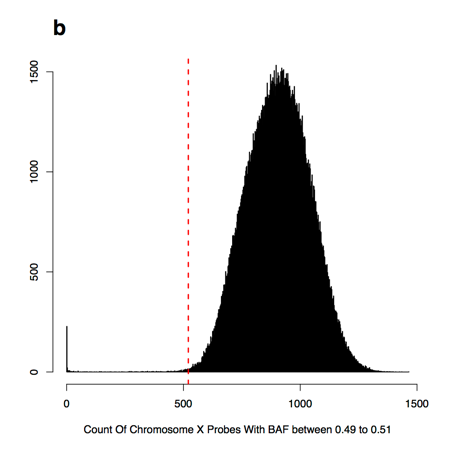


**Supplemental figure S2.** Plots (**a-ad**) represent Log R Ratio (LRR) and B Allele Frequency (BAF) in 30 samples that were identified as being full 45,X individuals (>80% 45,X)


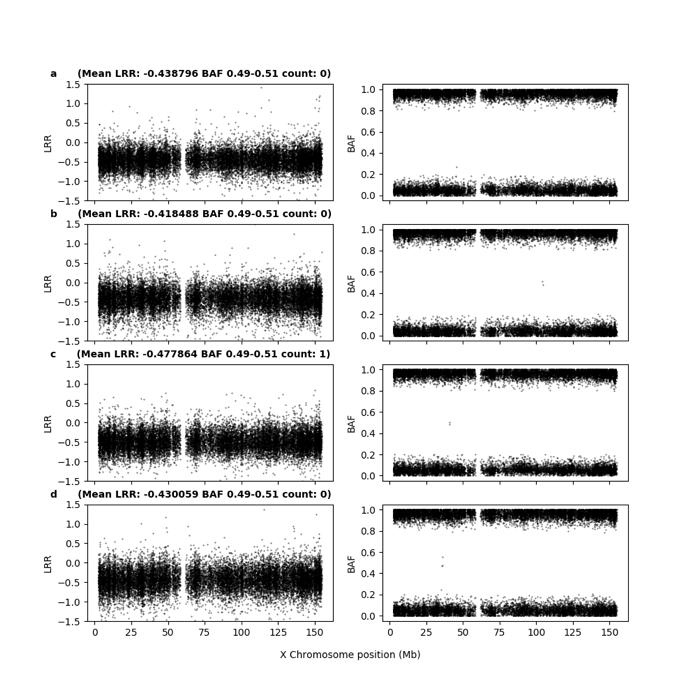

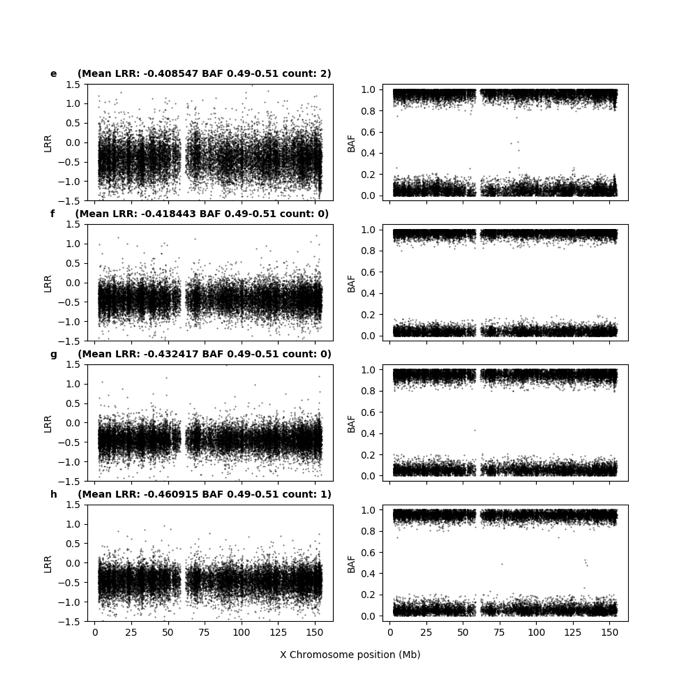

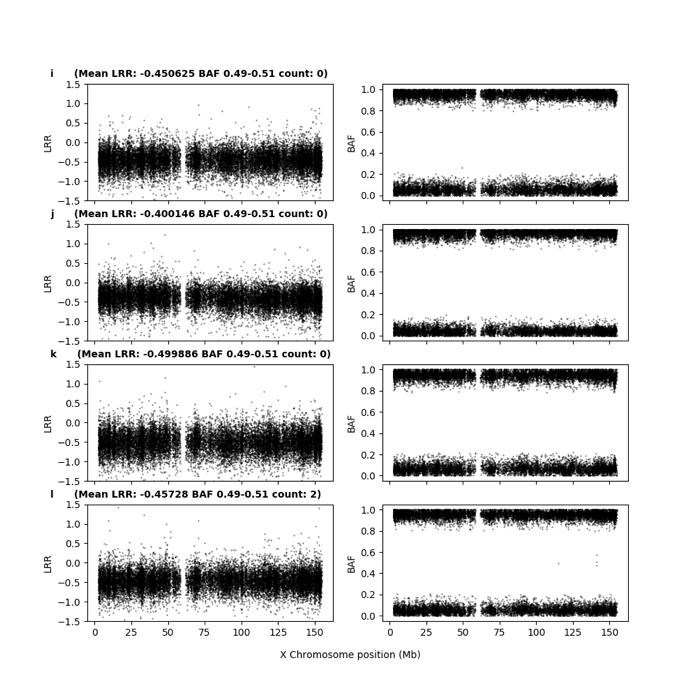

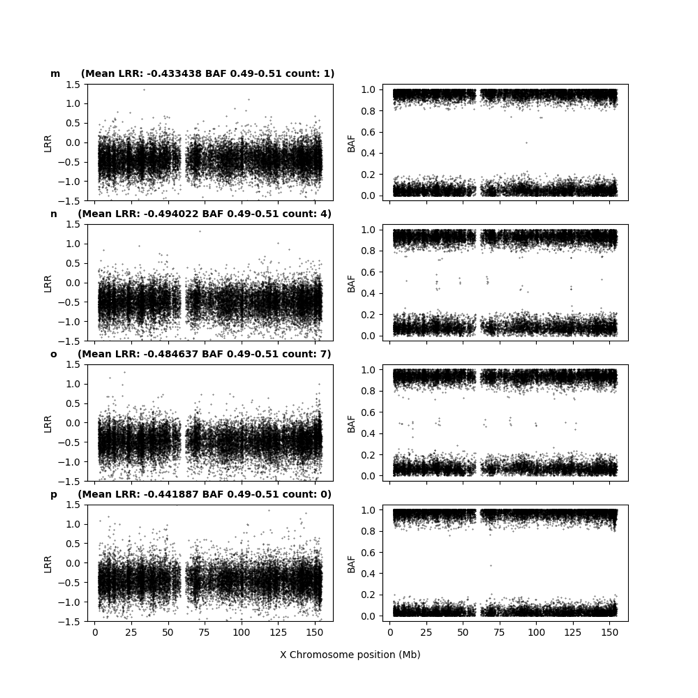

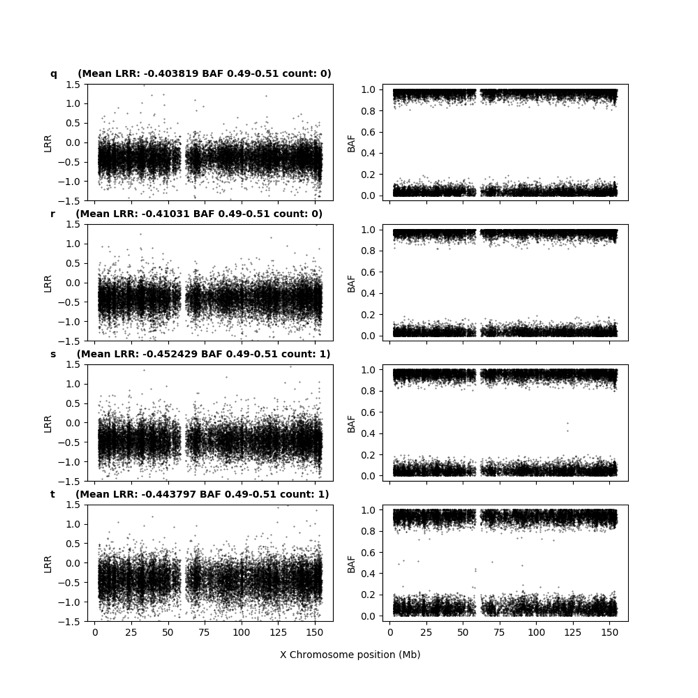

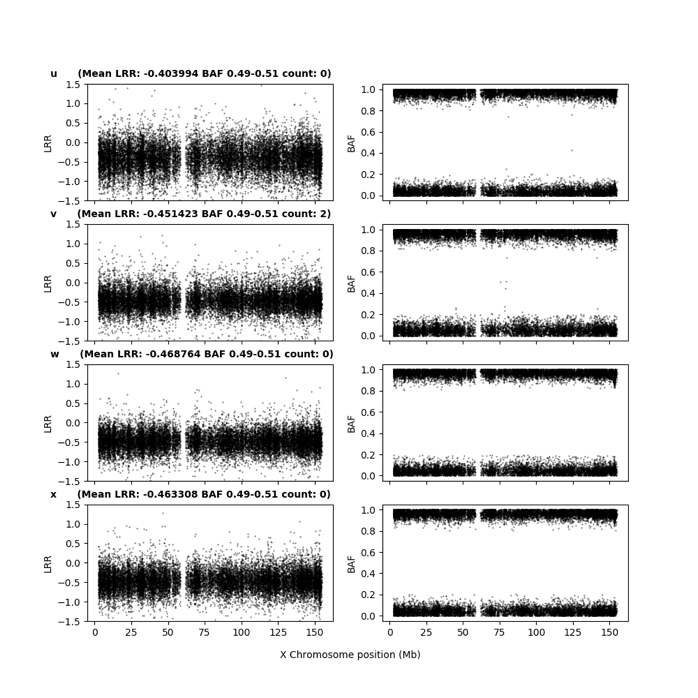

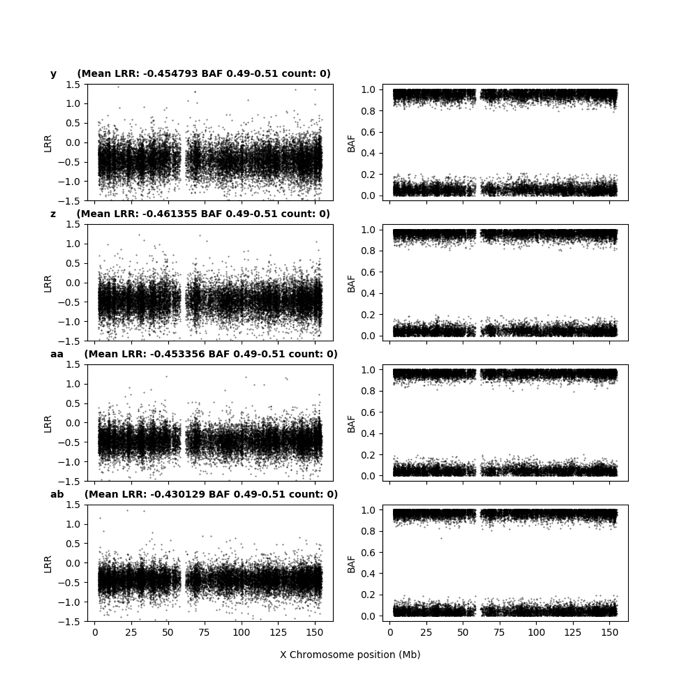

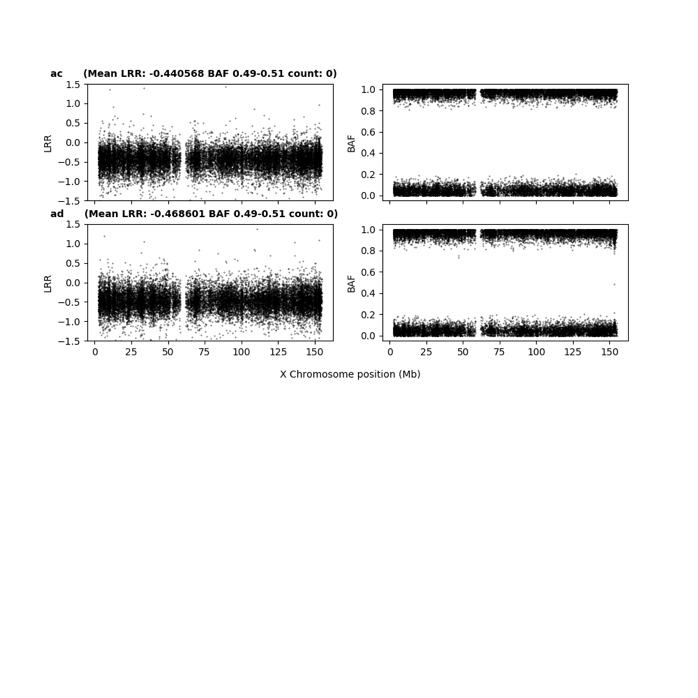


**Supplemental figure S3.** Plots (**a-gd**) represent Log R Ratio (LRR) and B Allele Frequency (BAF) in 186 samples that were identified as being 45,X/46,XX mosaic individuals (between 15% and 80% 45,X)


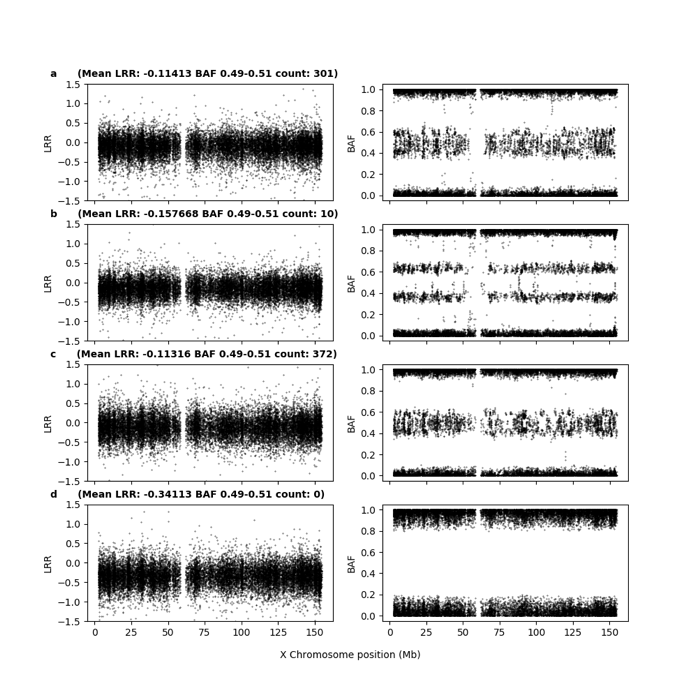

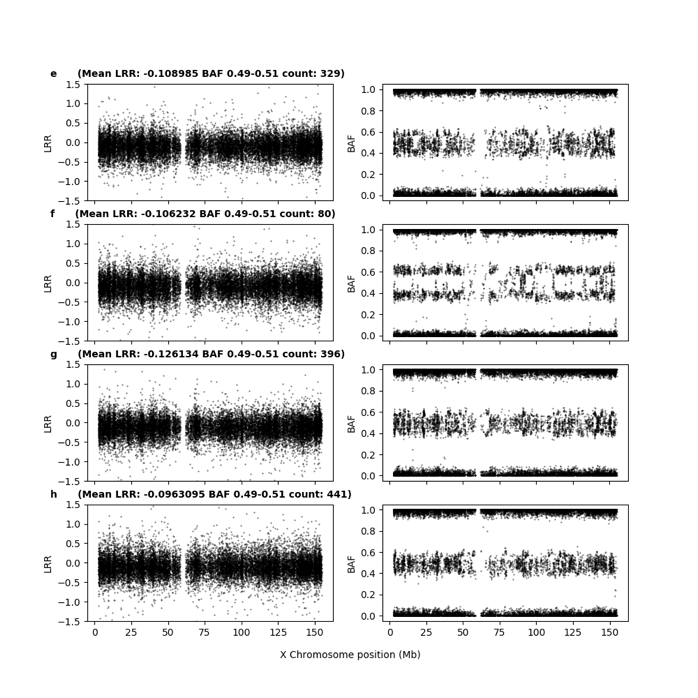

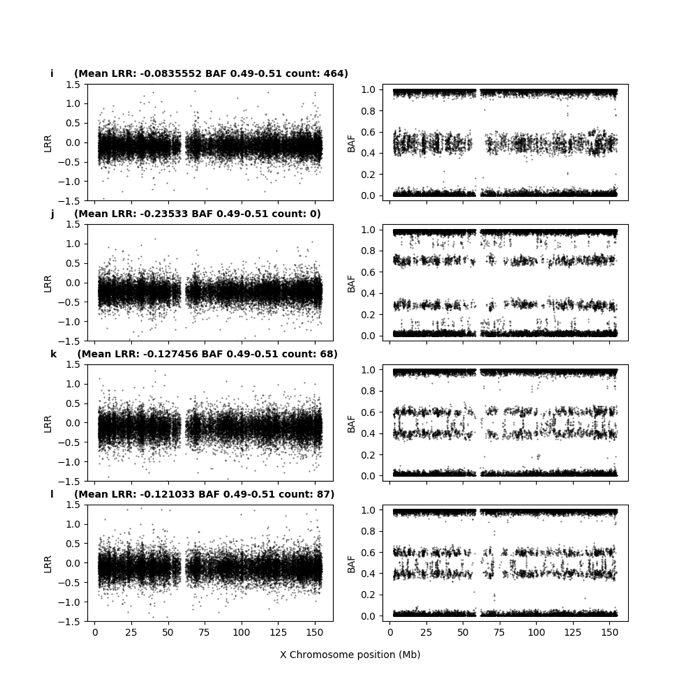

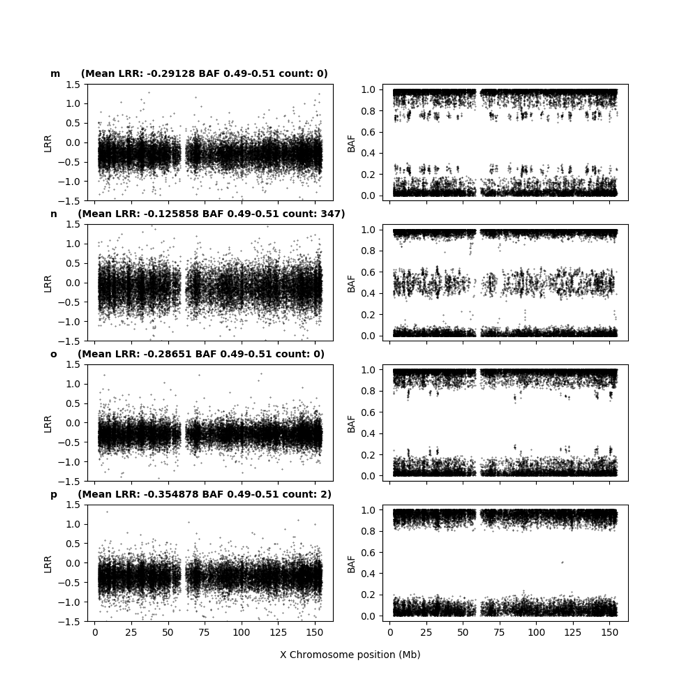

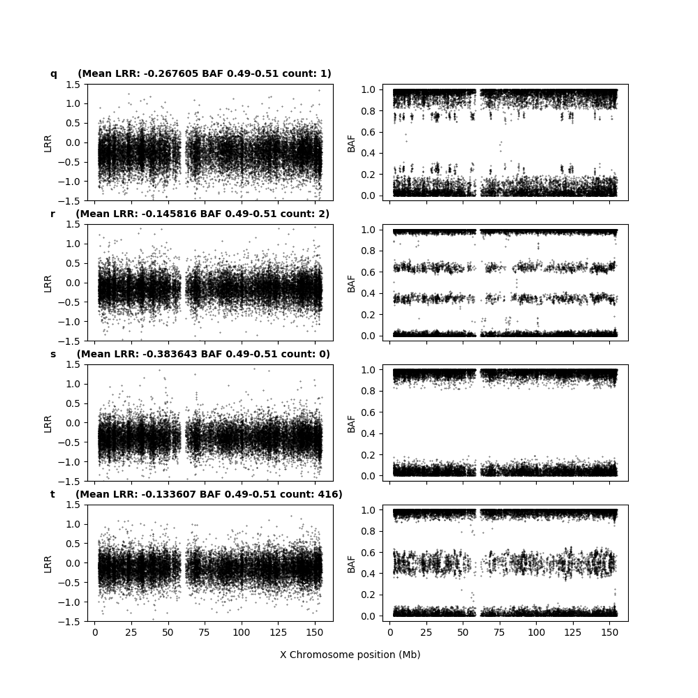

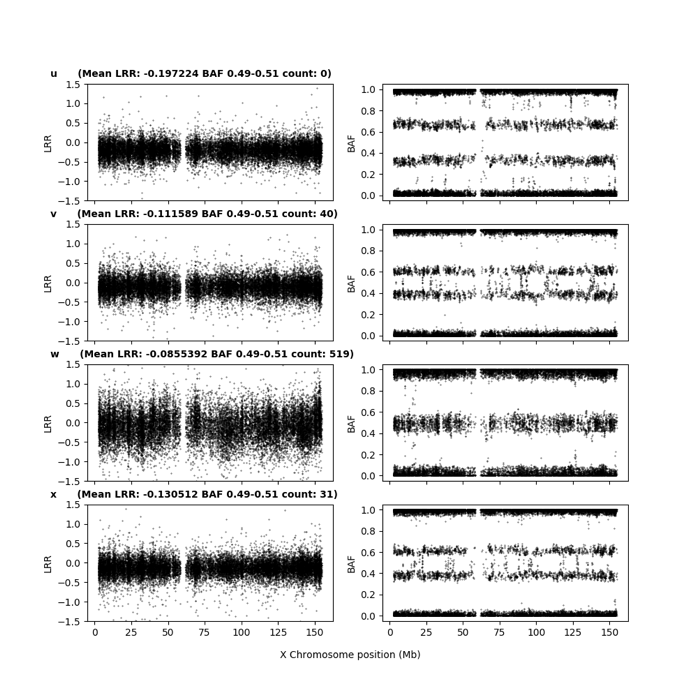

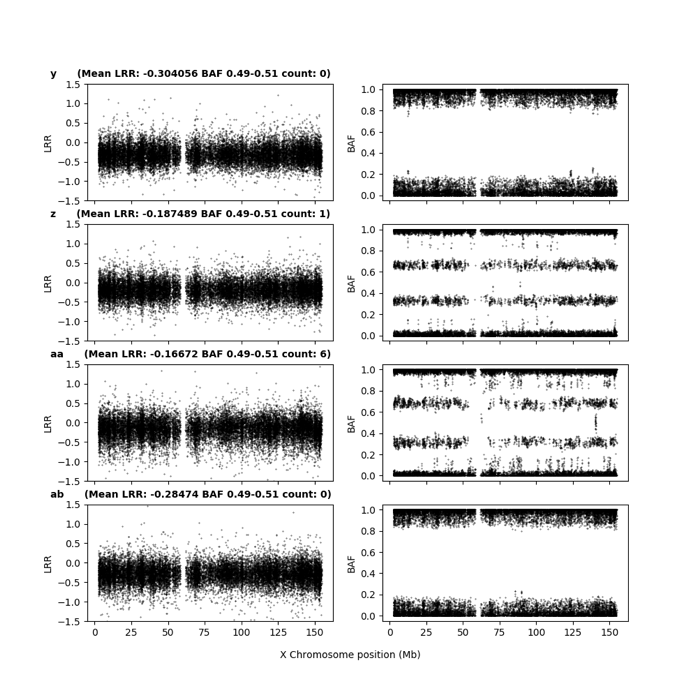

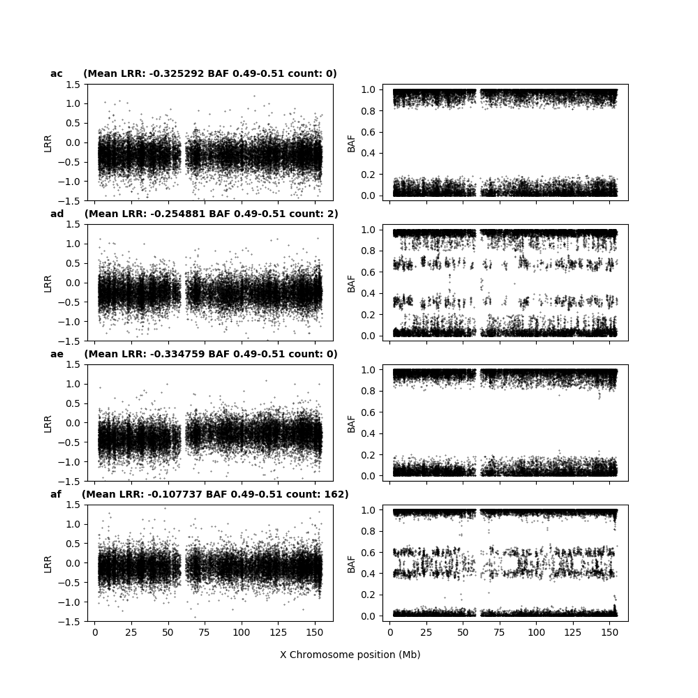

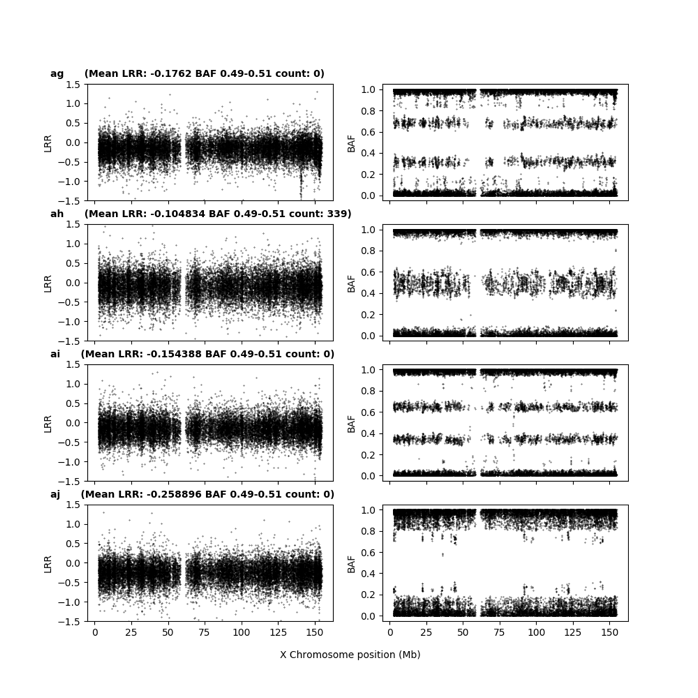

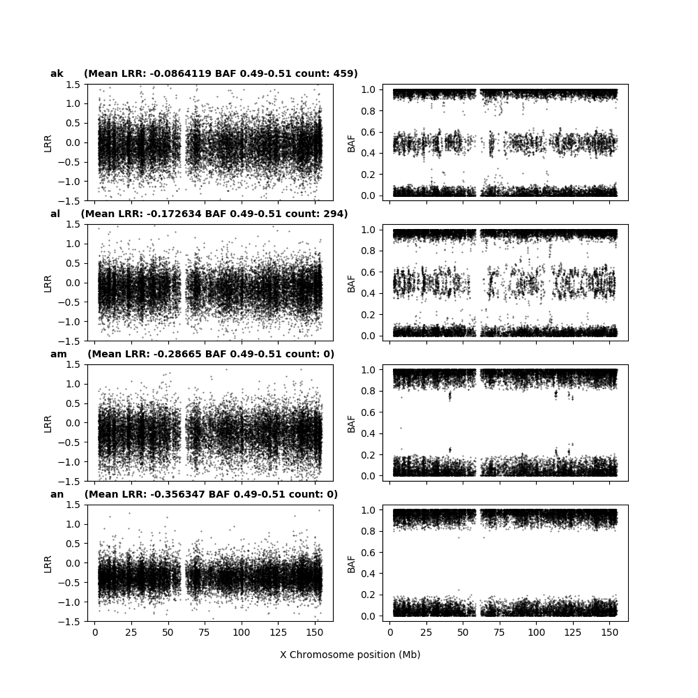

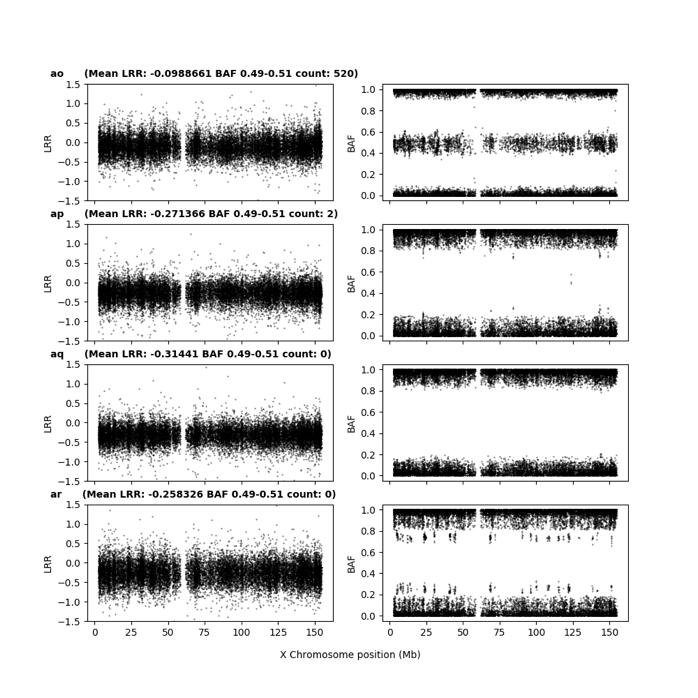

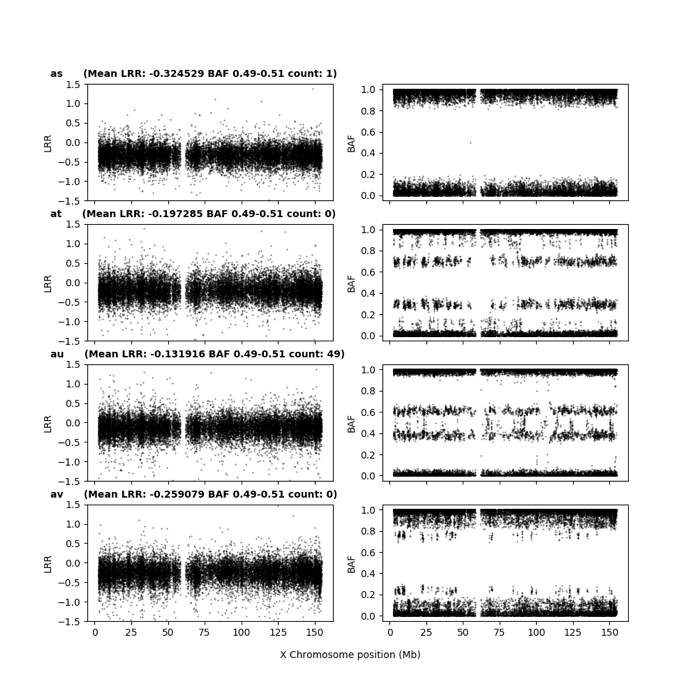

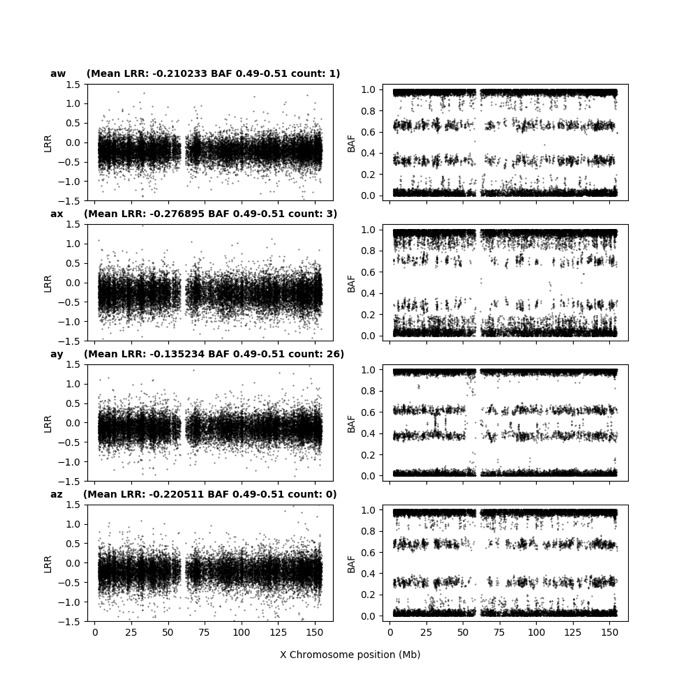

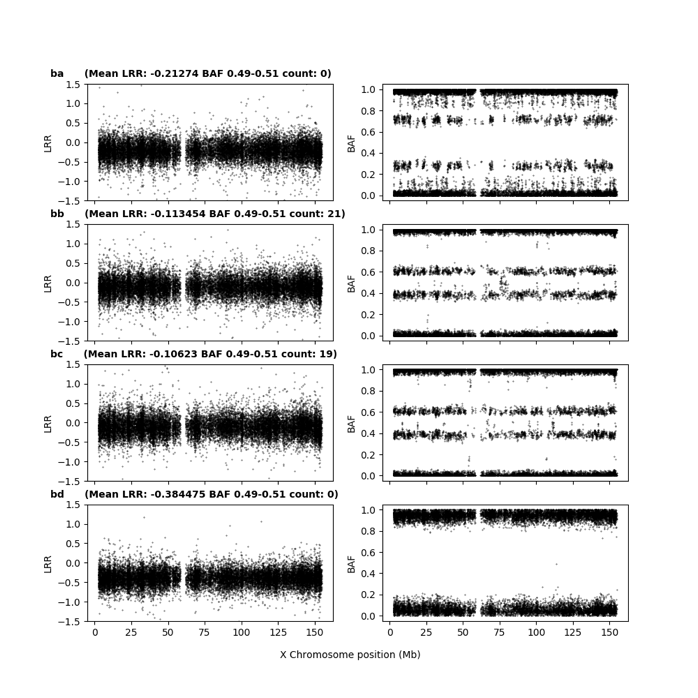

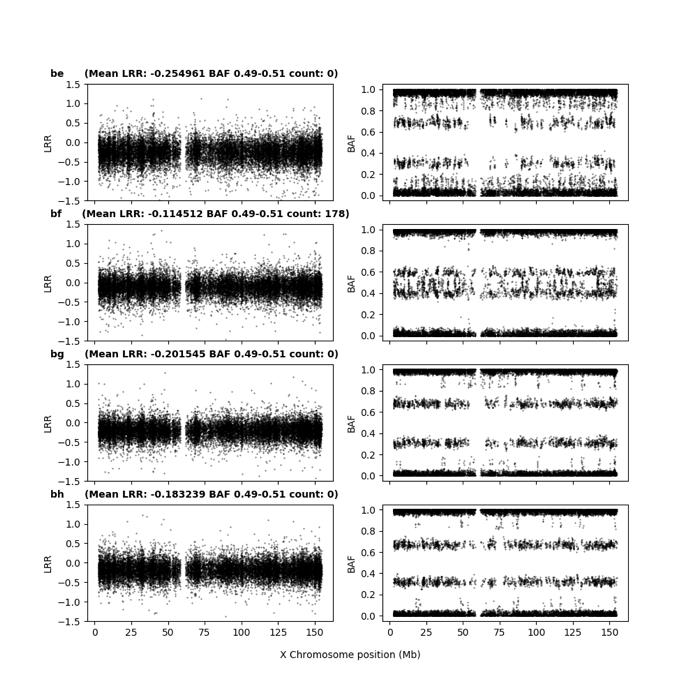

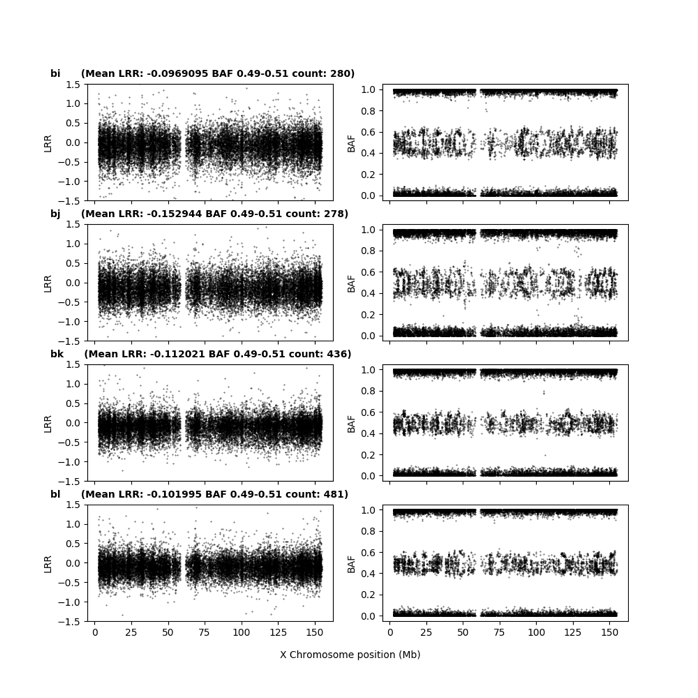

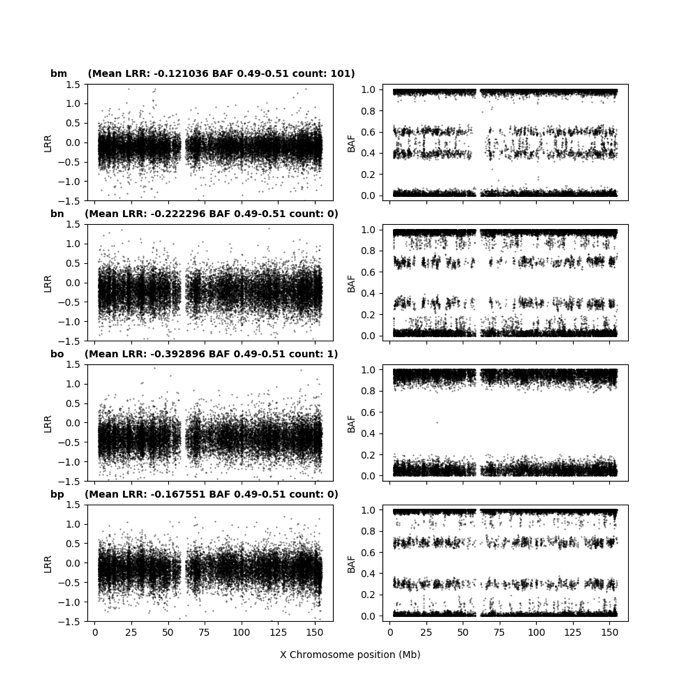

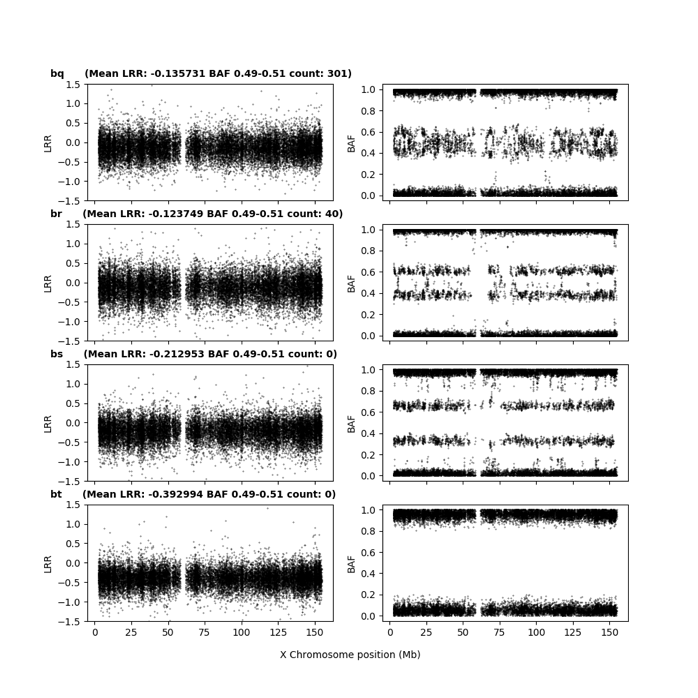

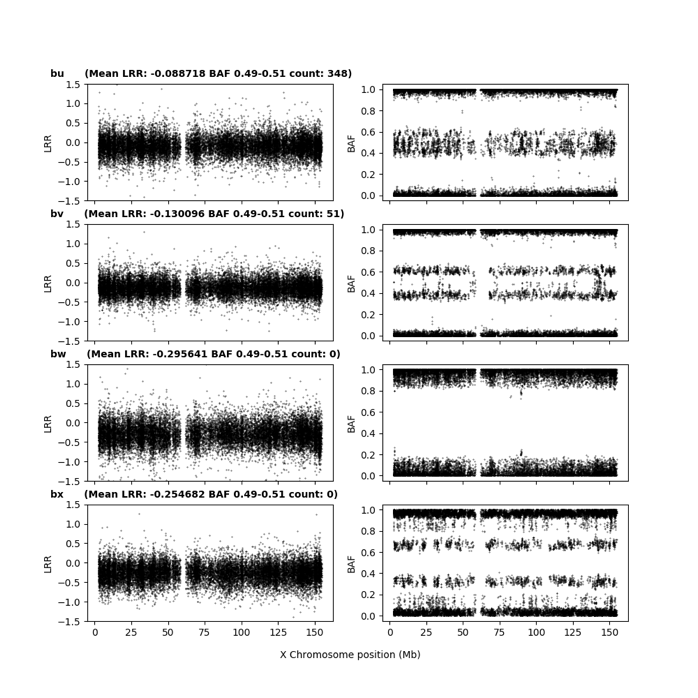

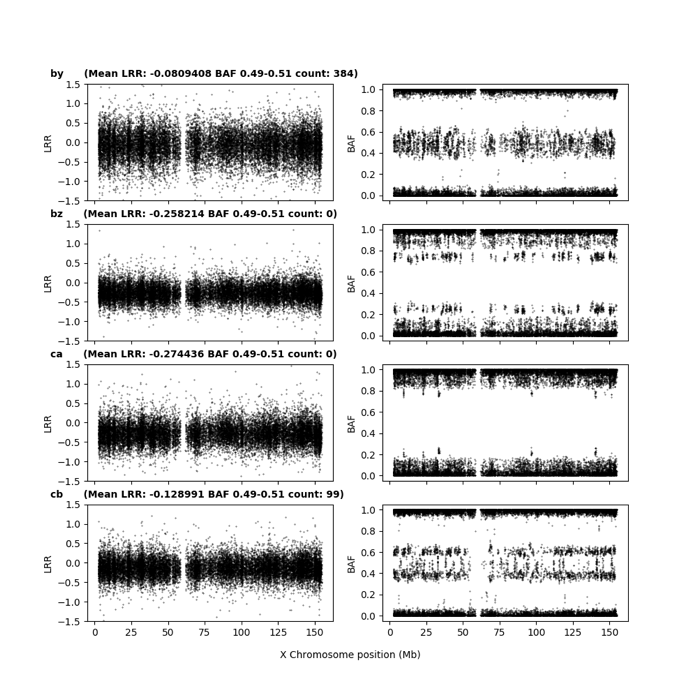

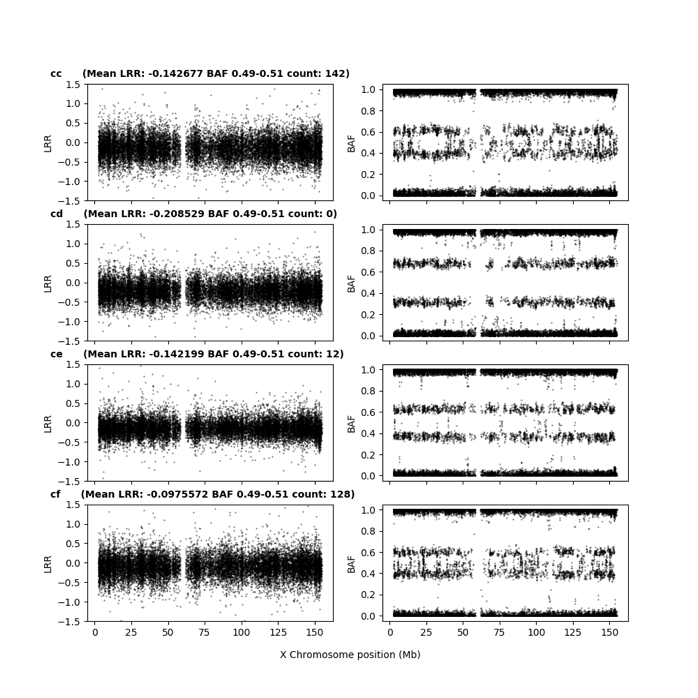

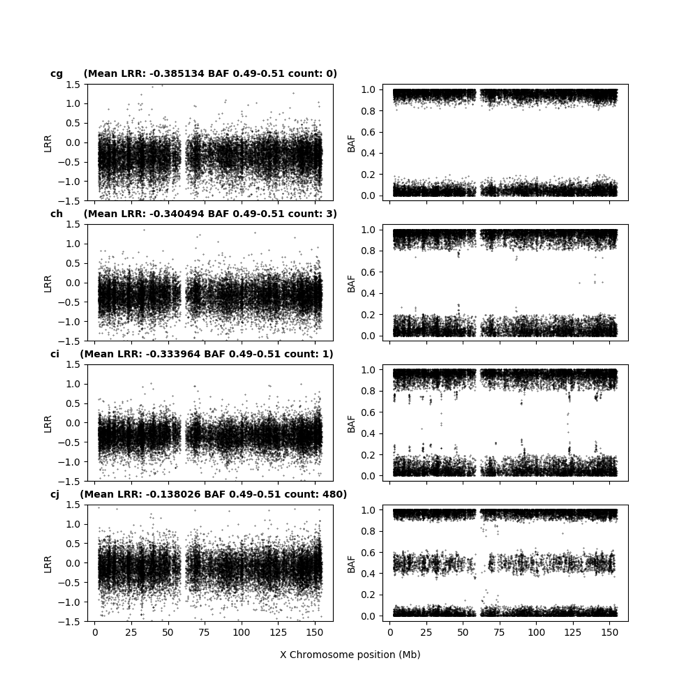

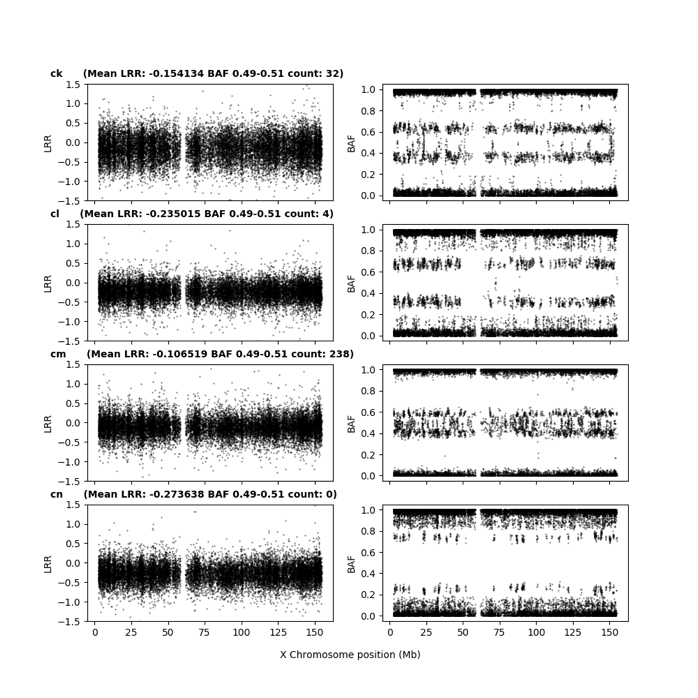

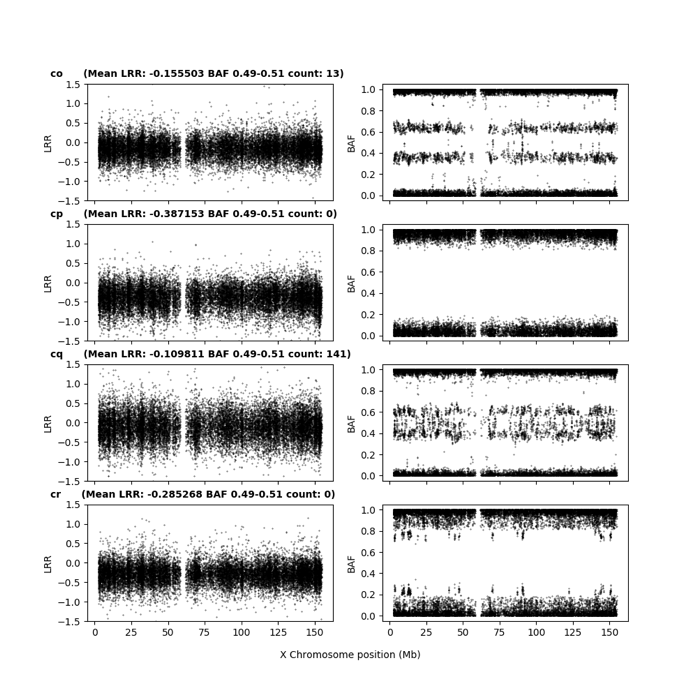

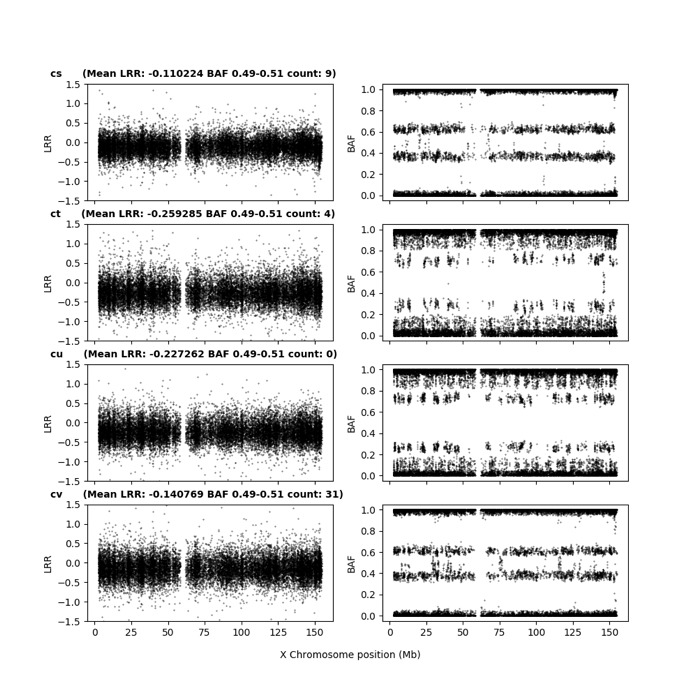

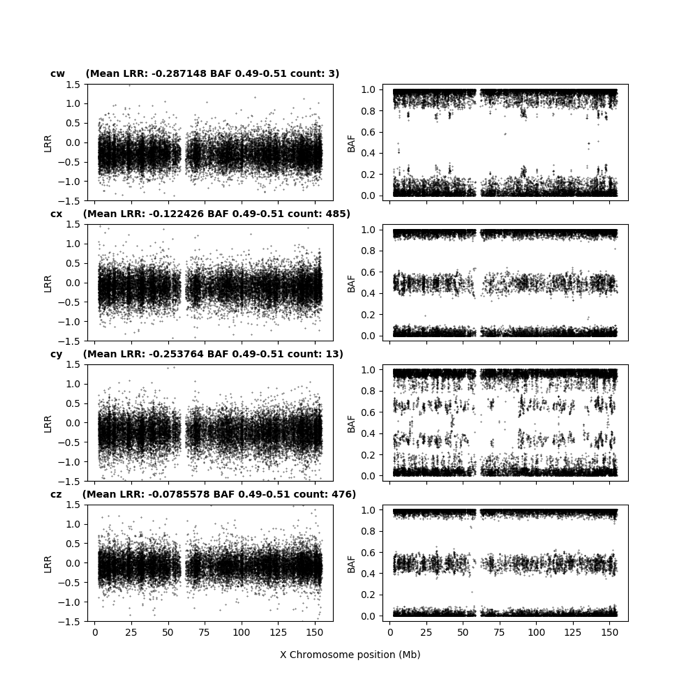

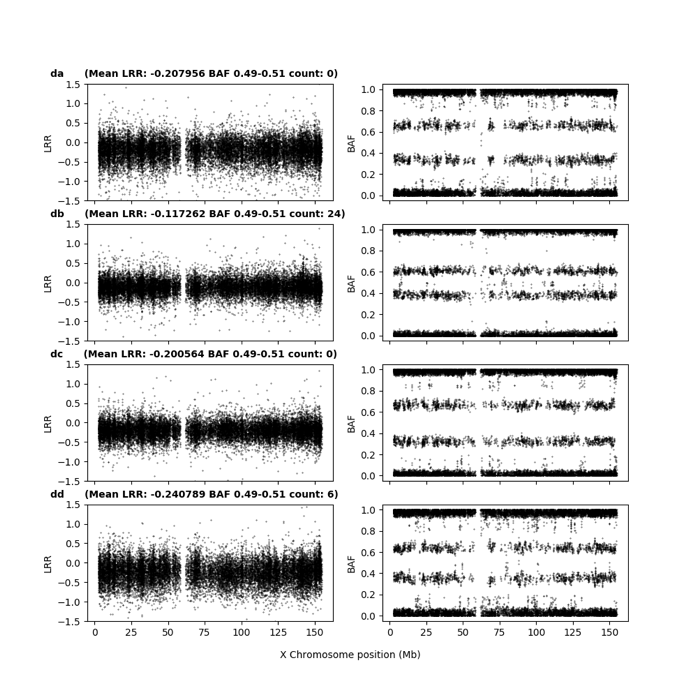

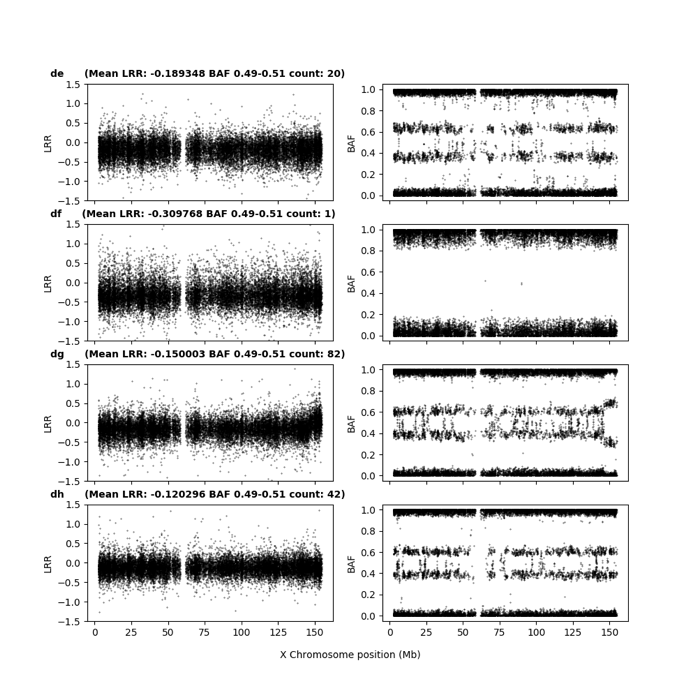

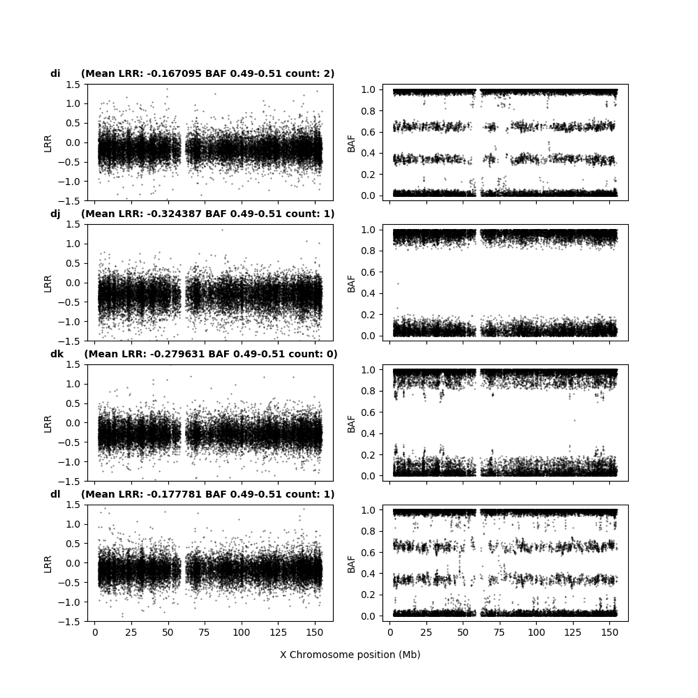

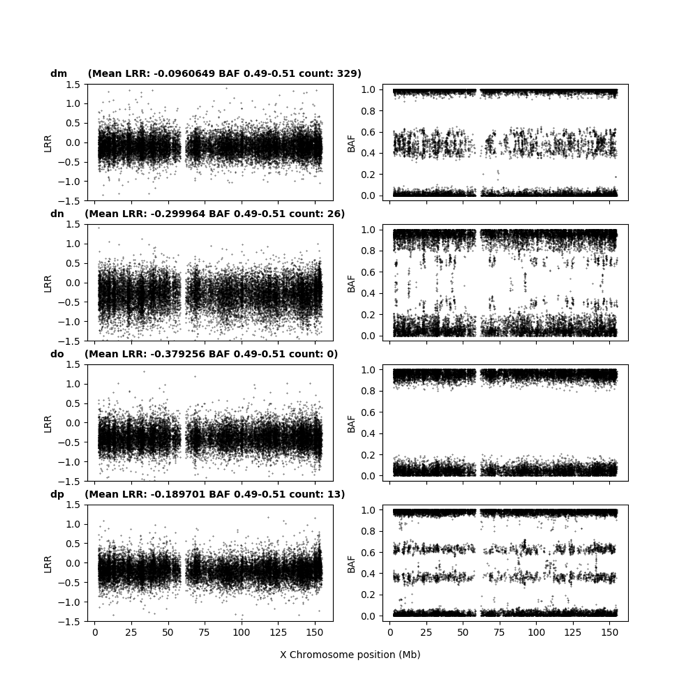

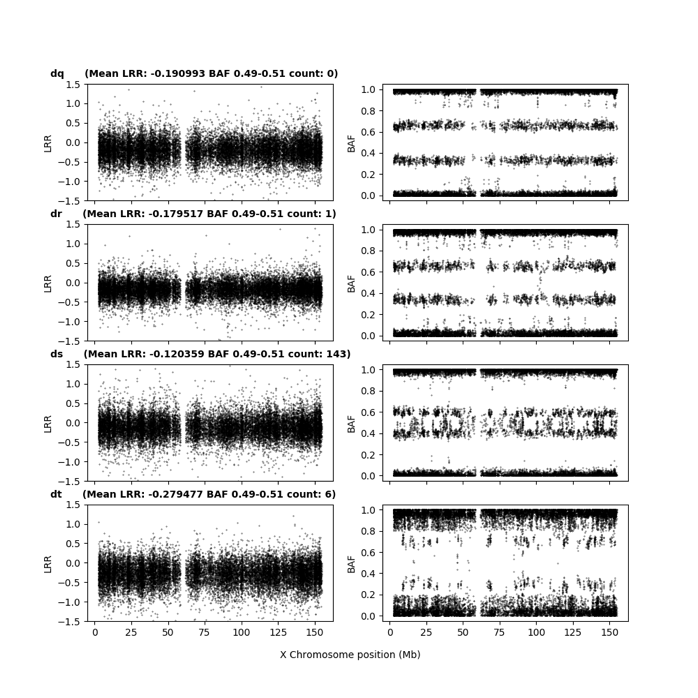

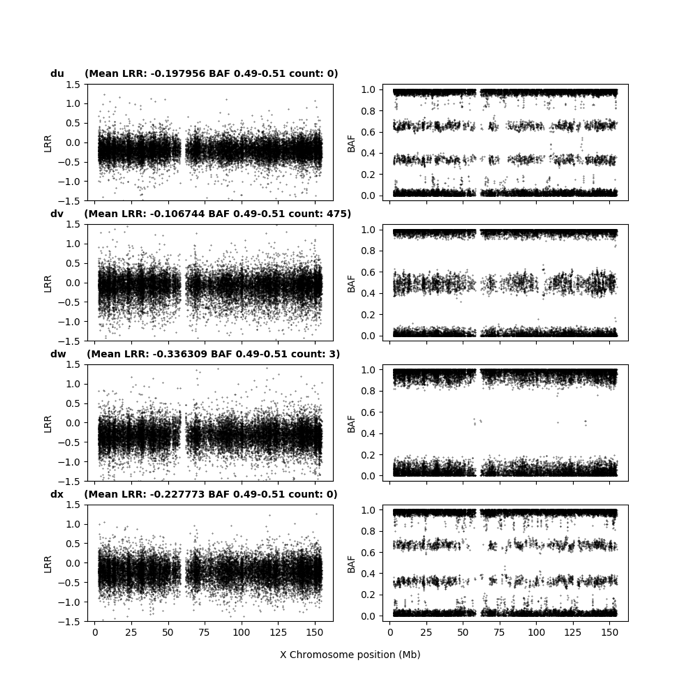

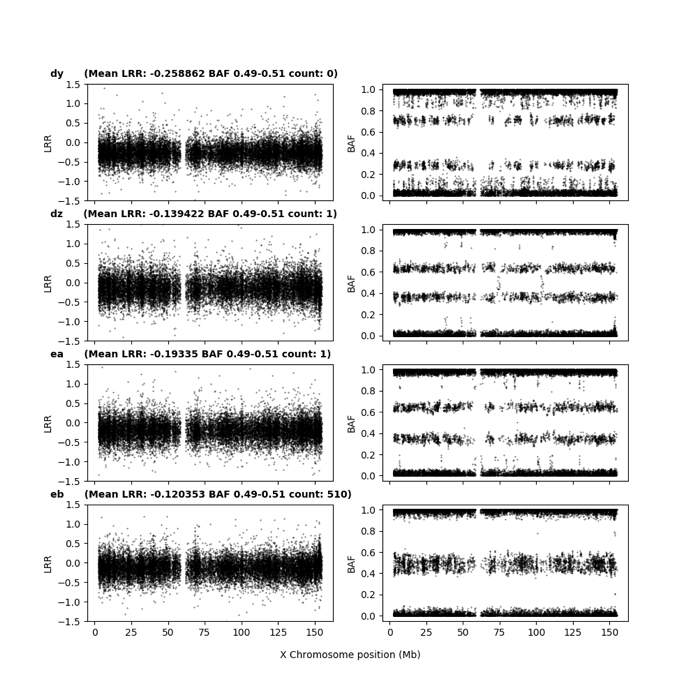

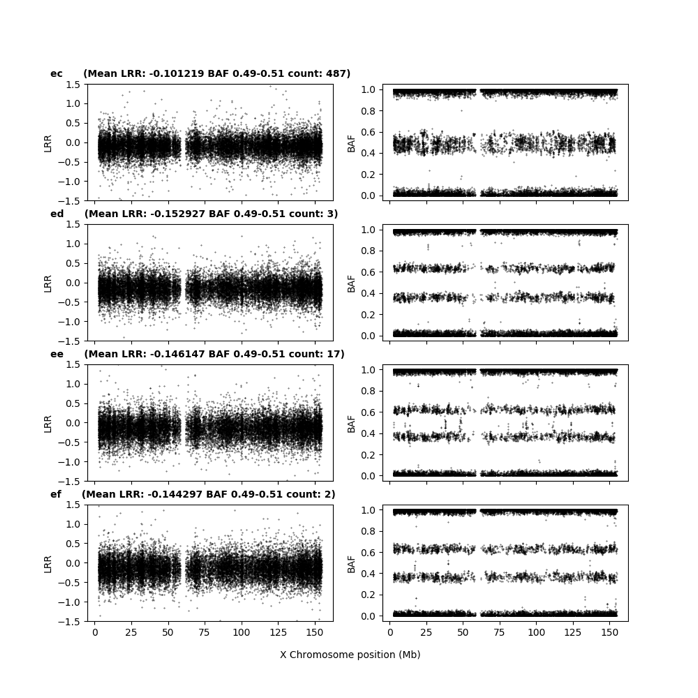

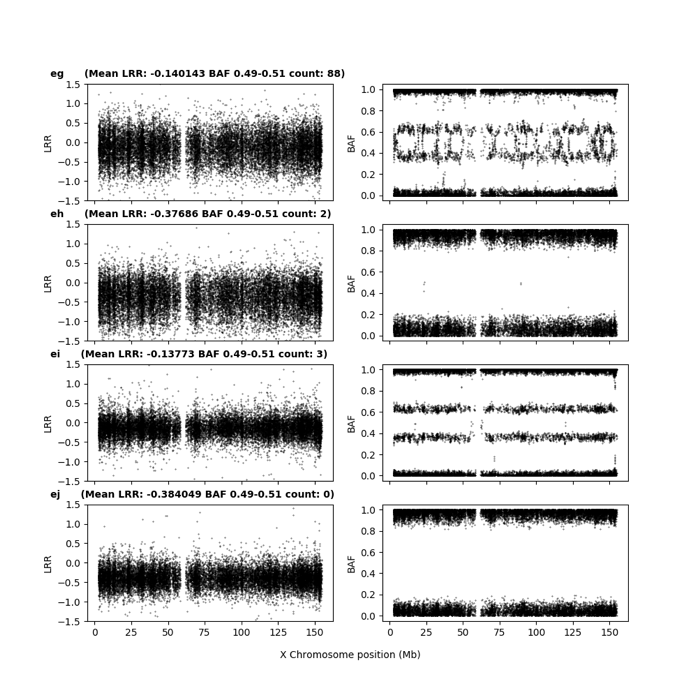

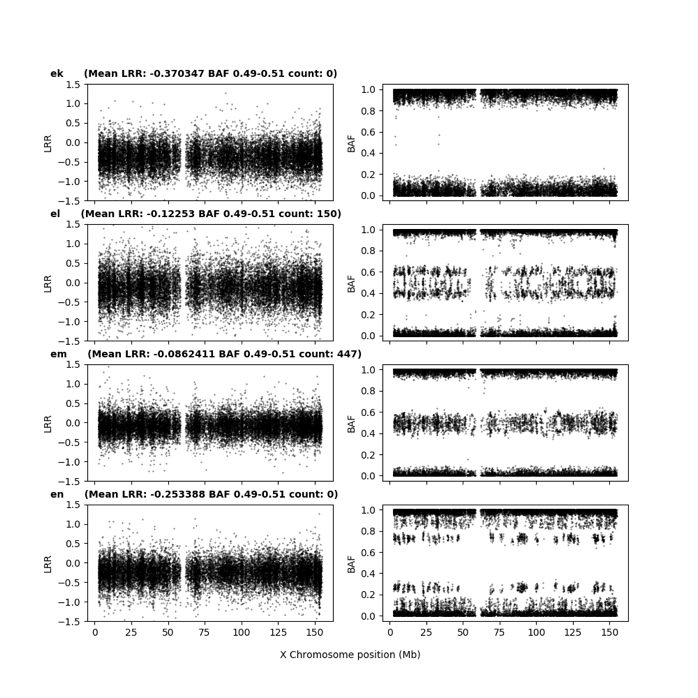

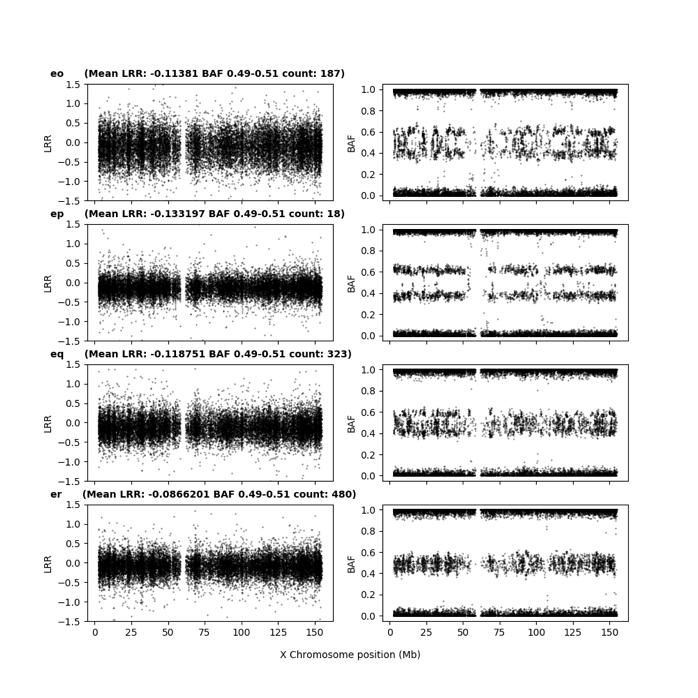

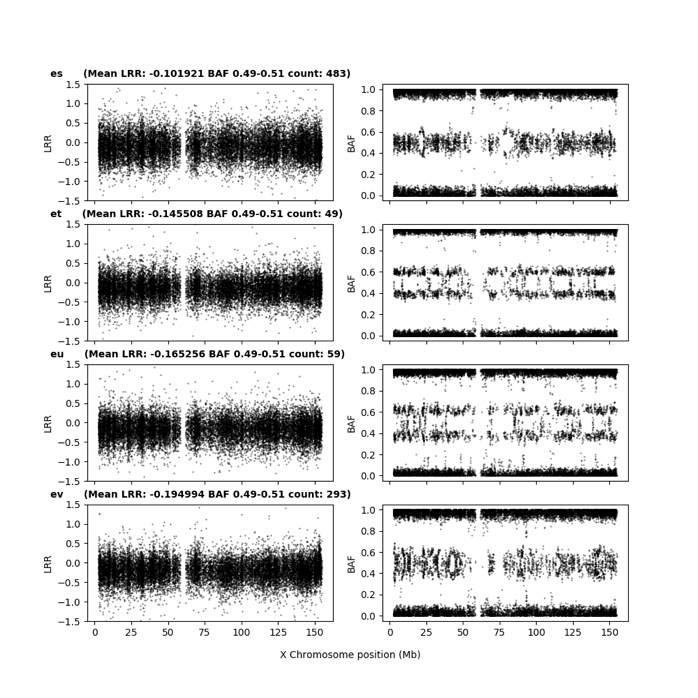

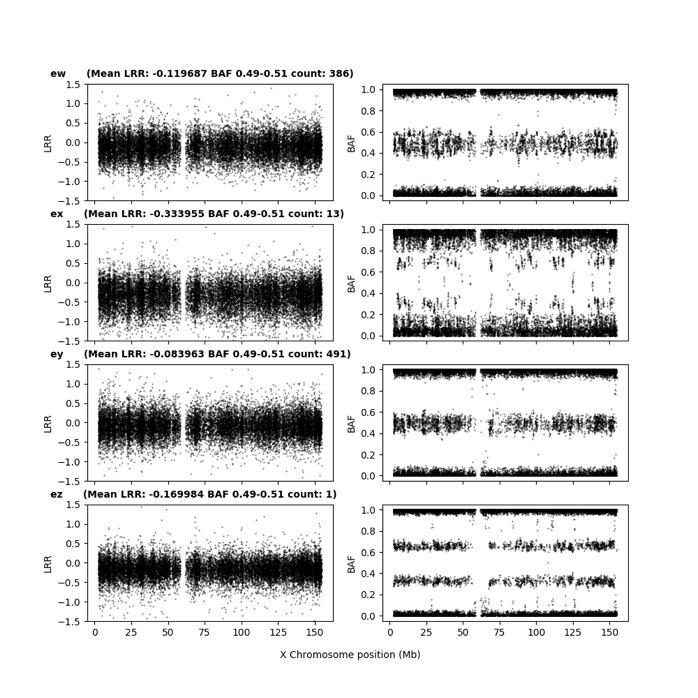

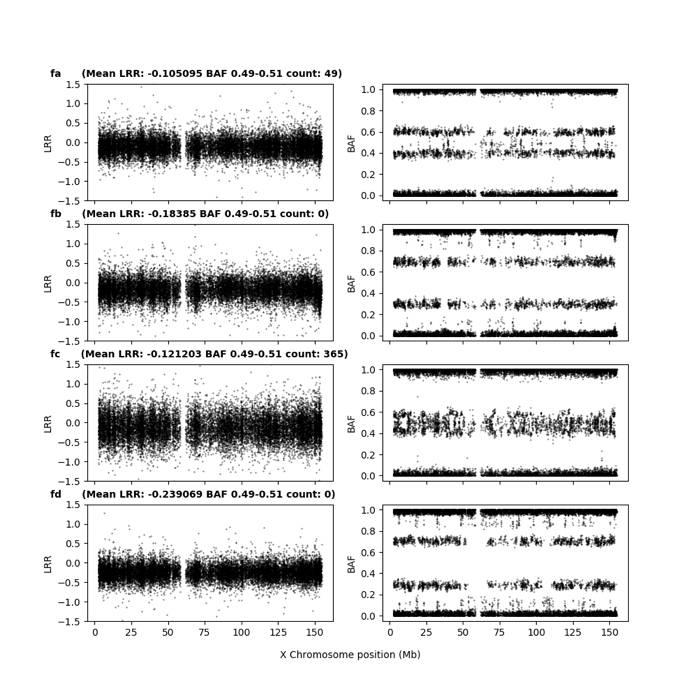

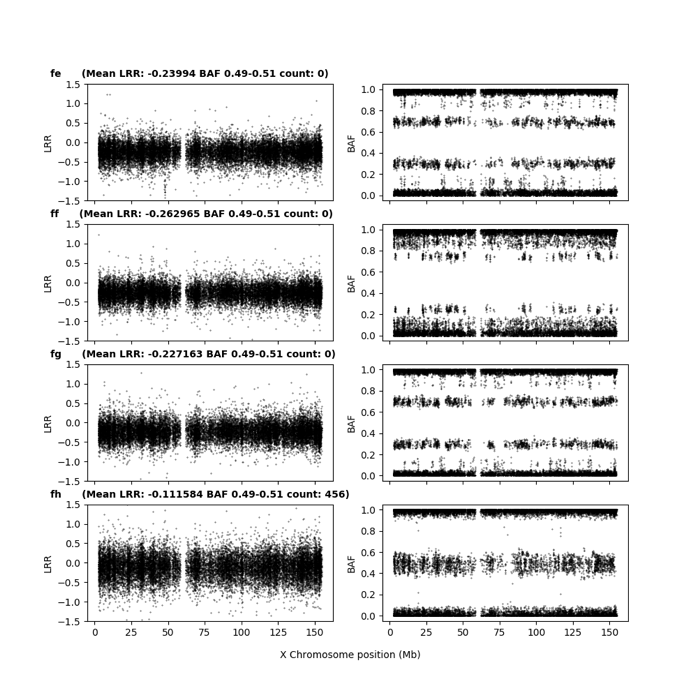

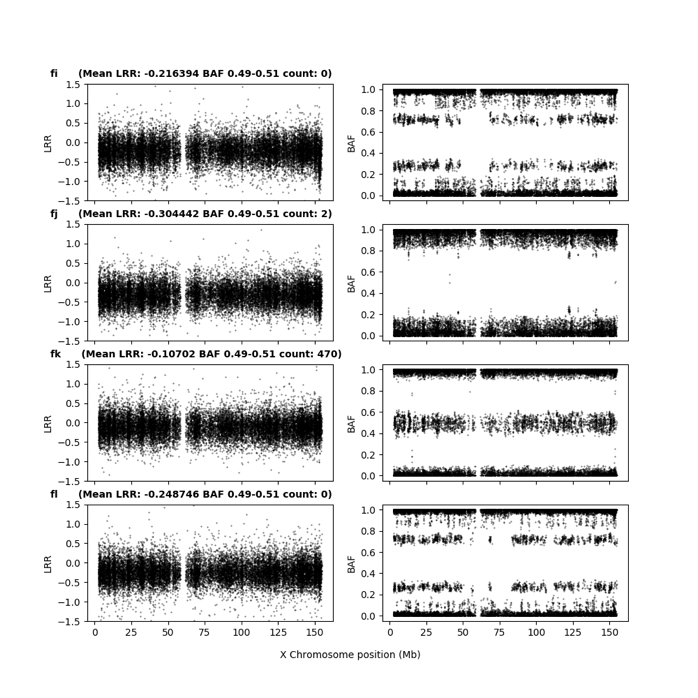

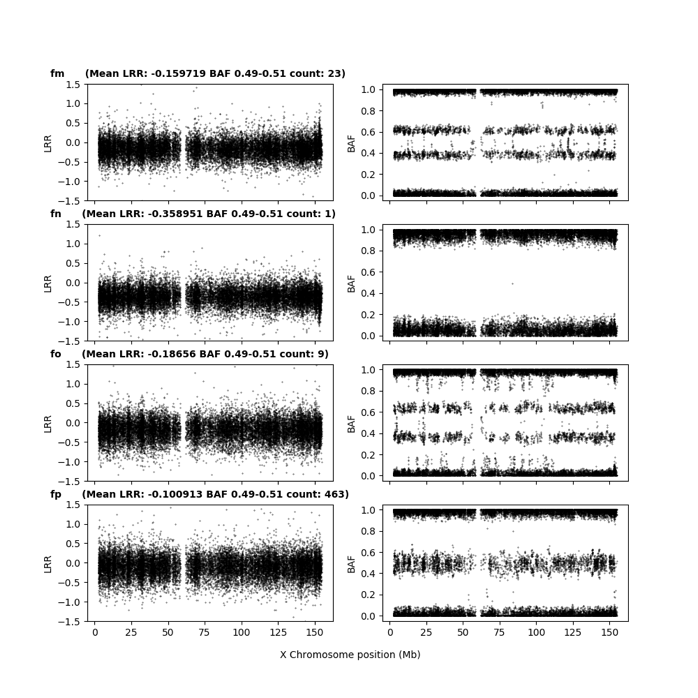

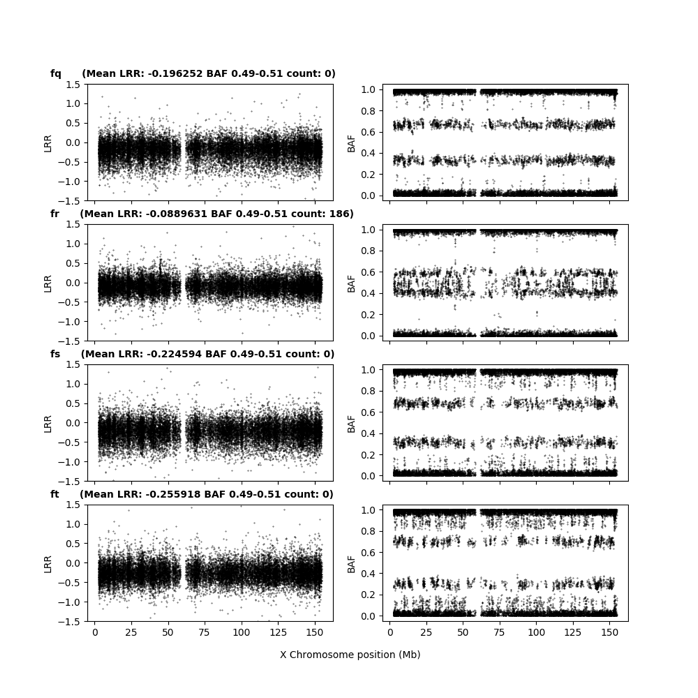

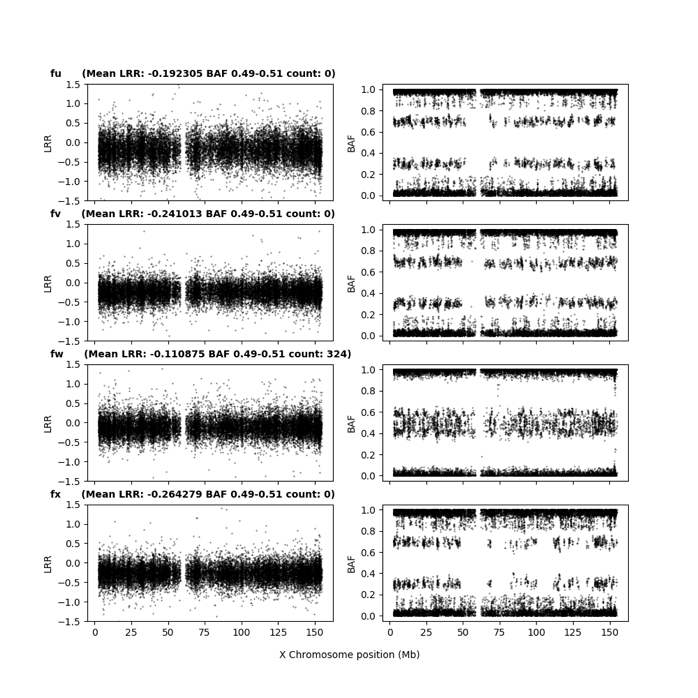

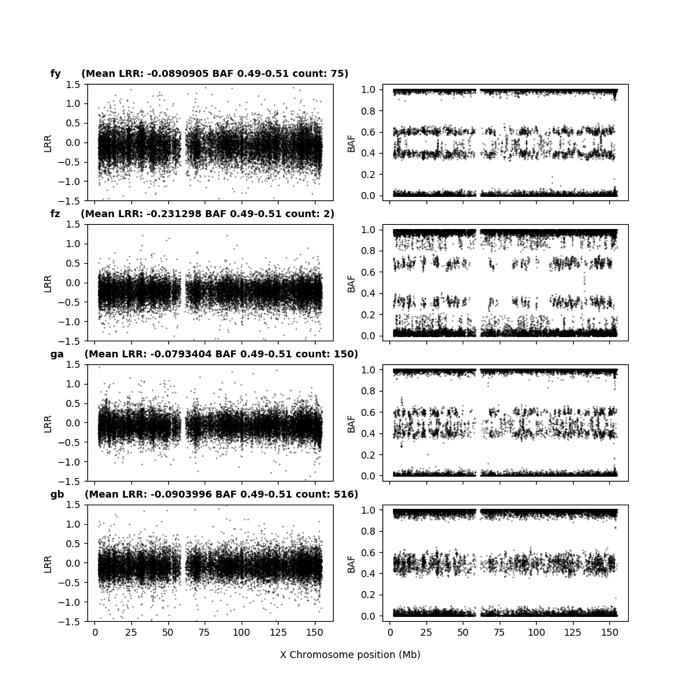

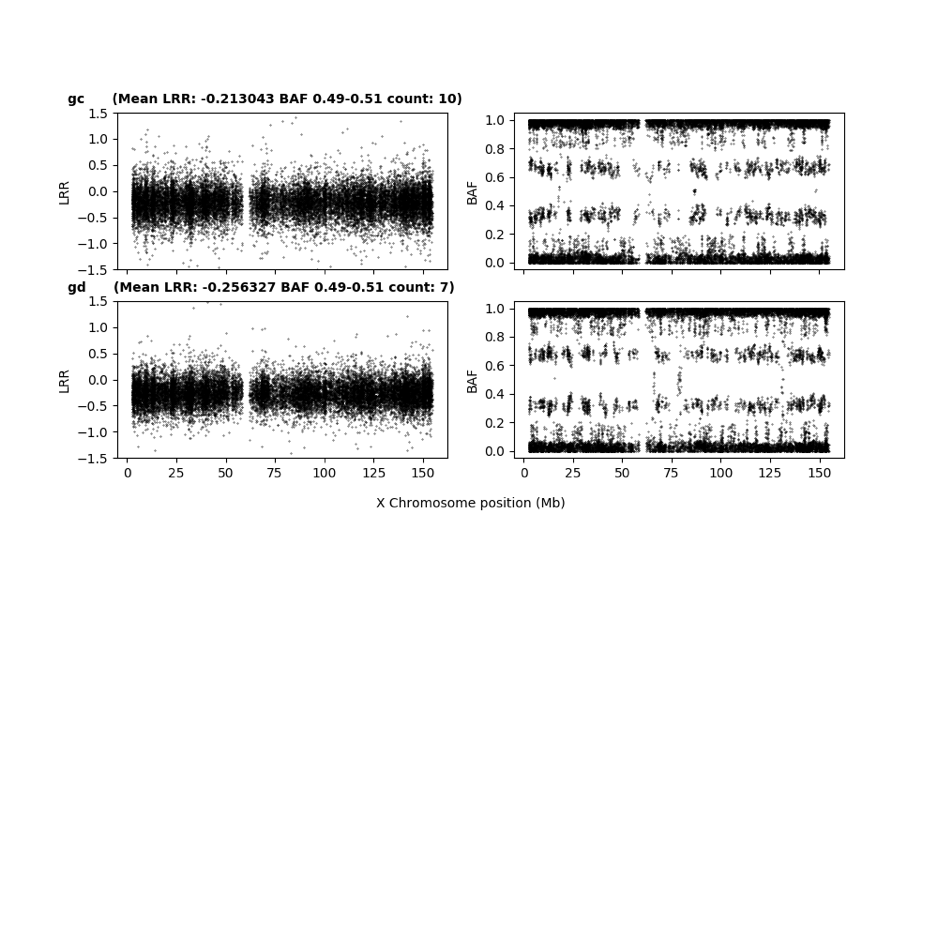


**Supplemental figure S4.** Plots (**a-df**) represent Log R Ratio (LRR) and B Allele Frequency (BAF) in 110 samples that were estimated as being full 47,XXX individuals


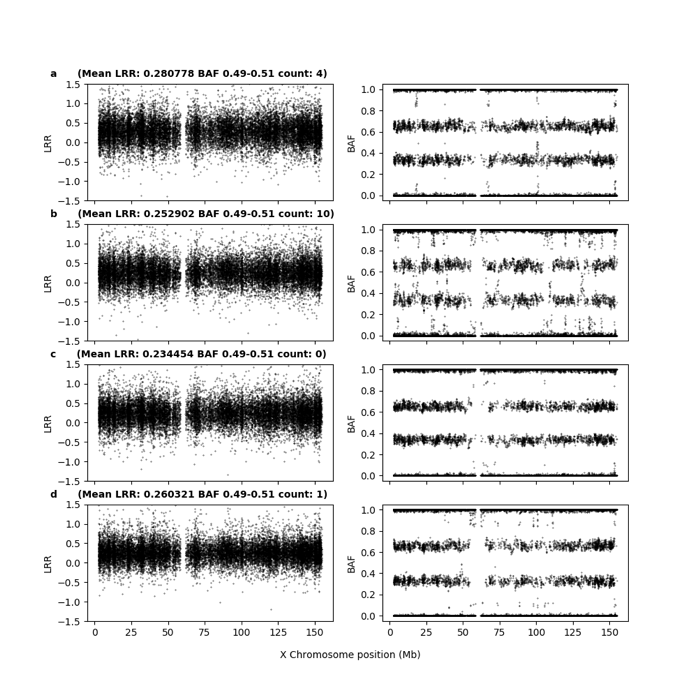
**
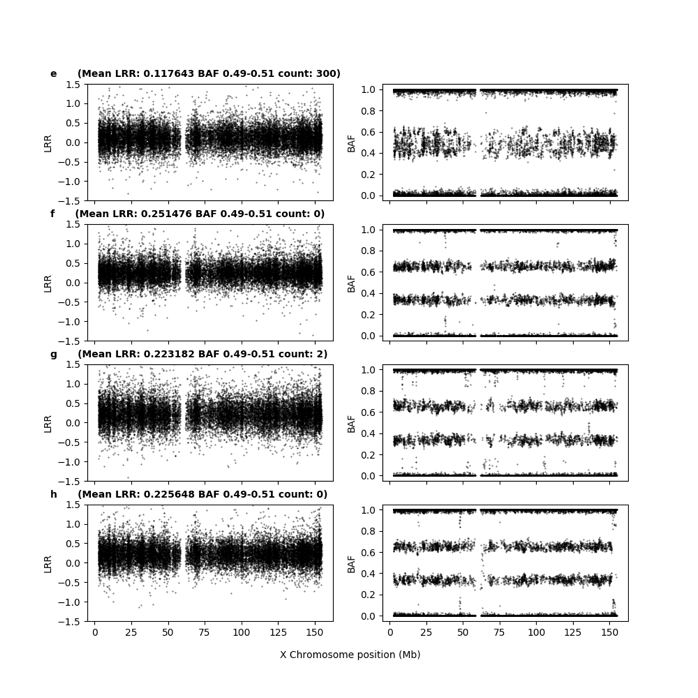

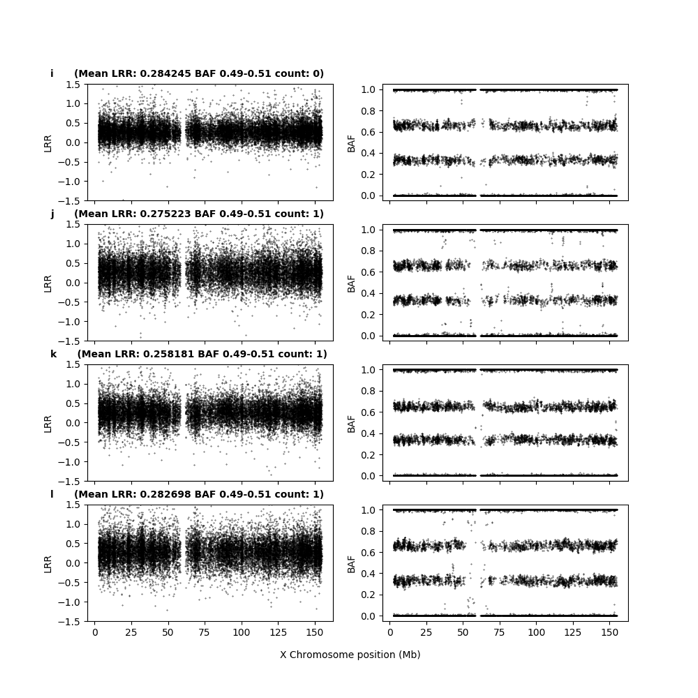

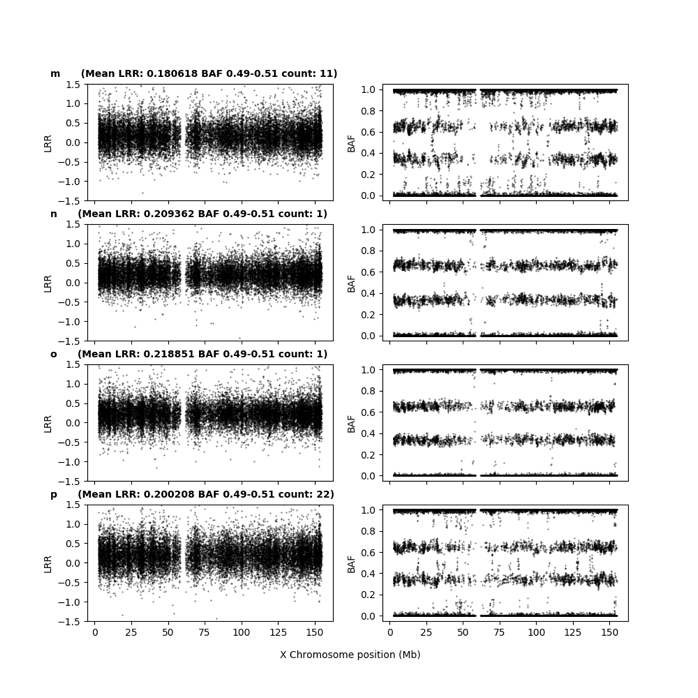

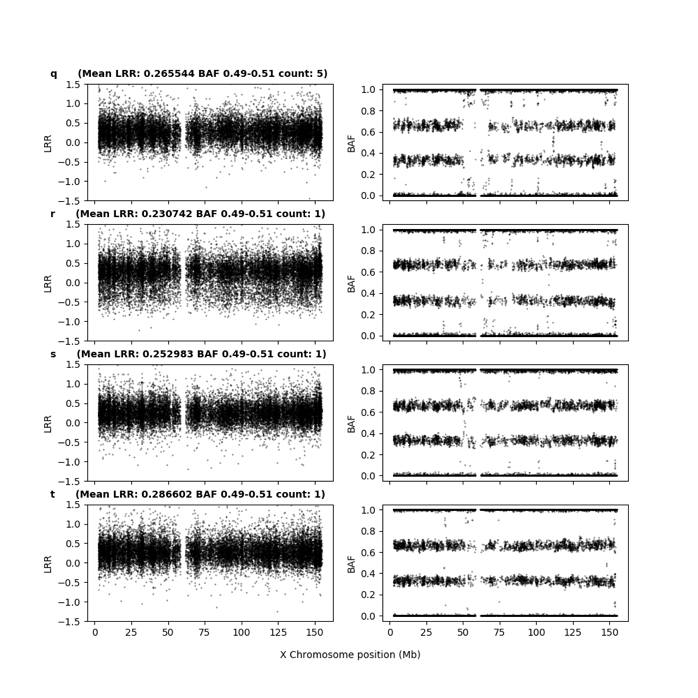

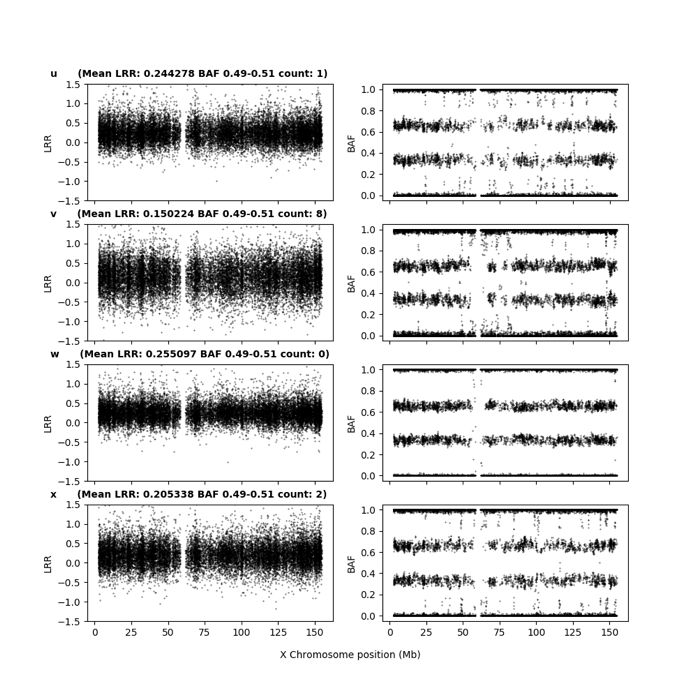

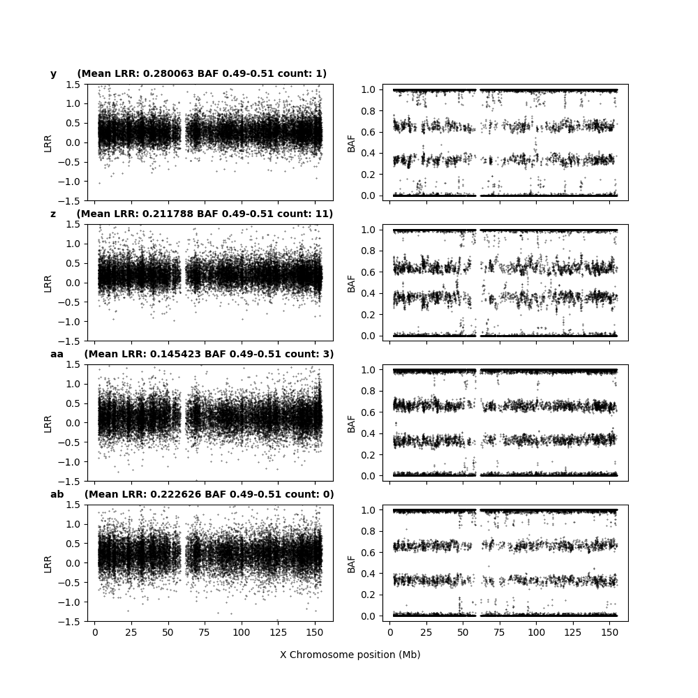

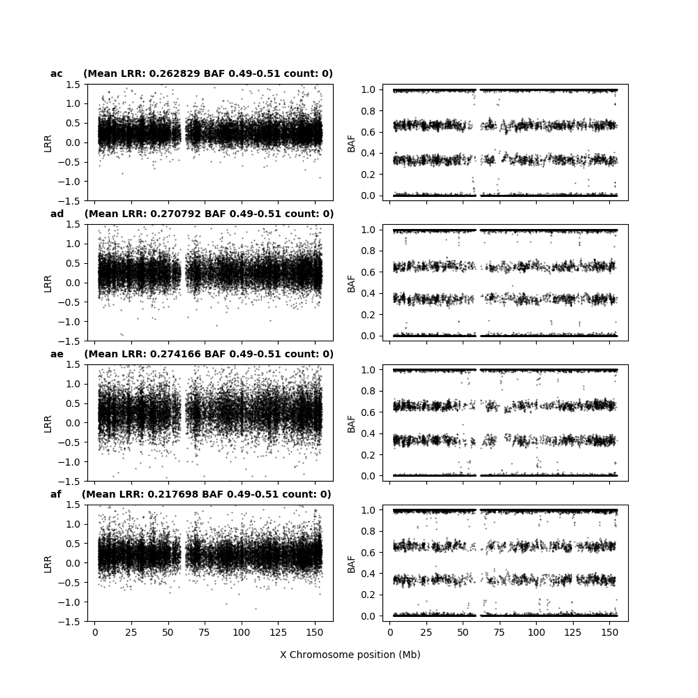

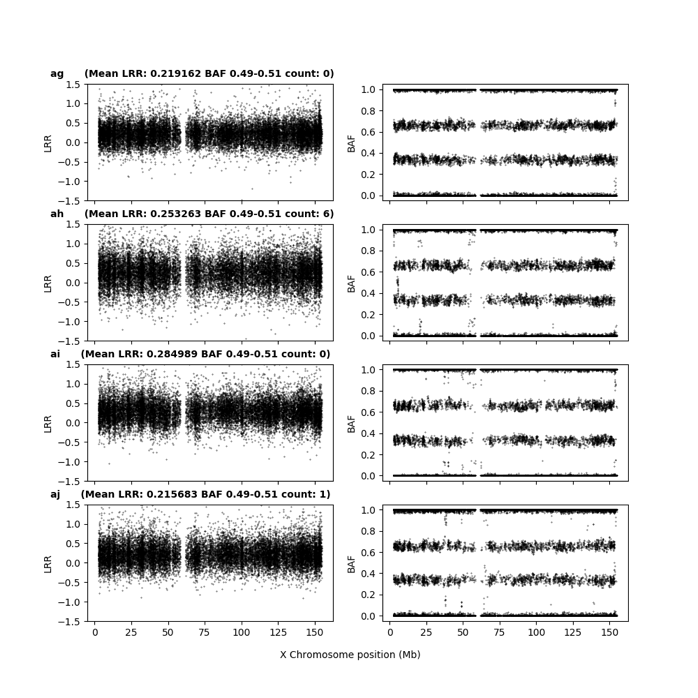

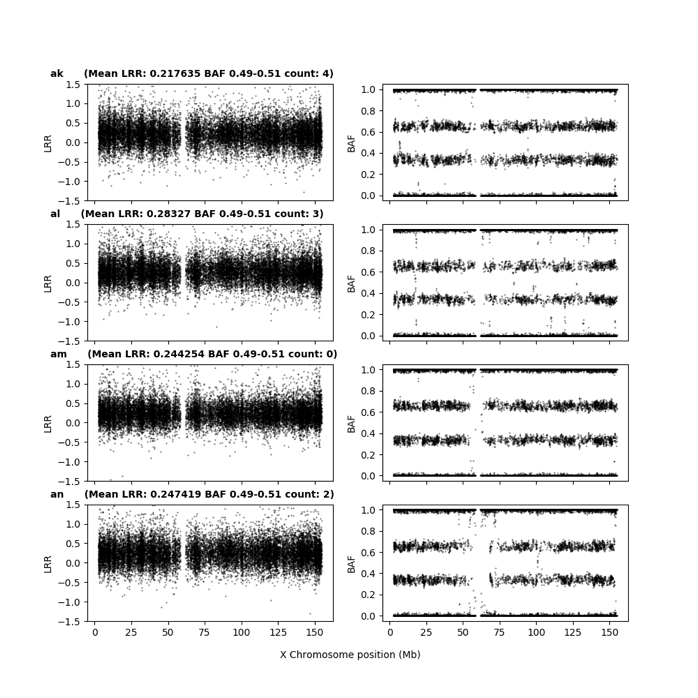

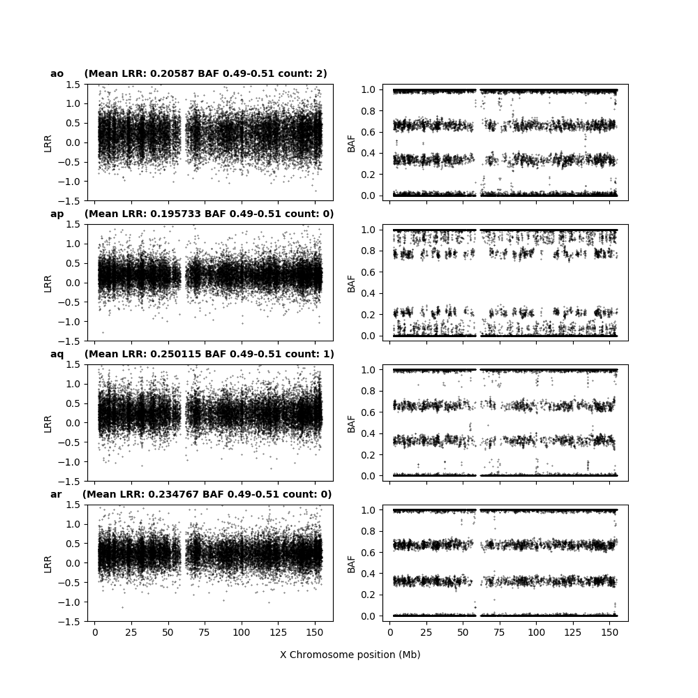

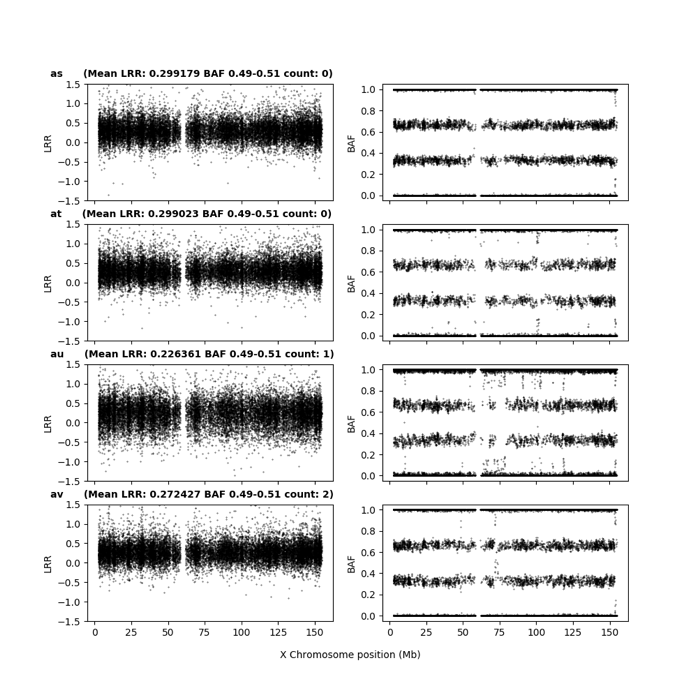

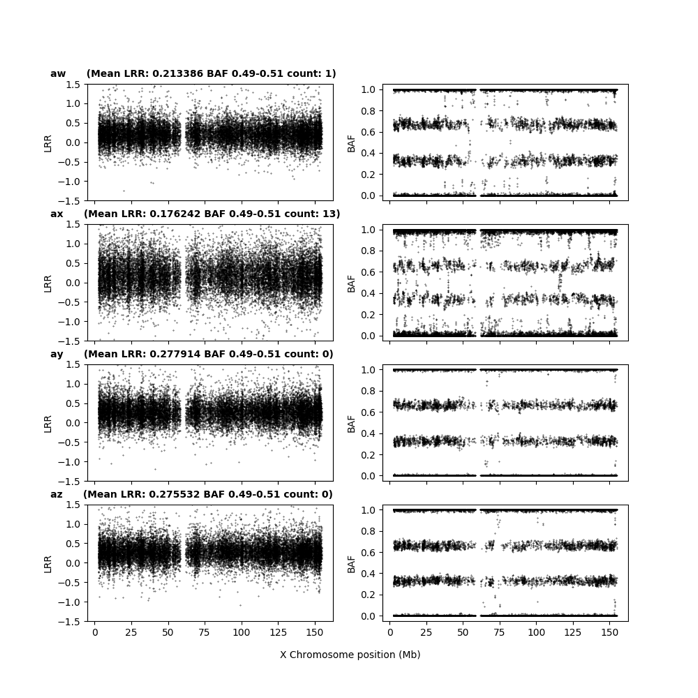

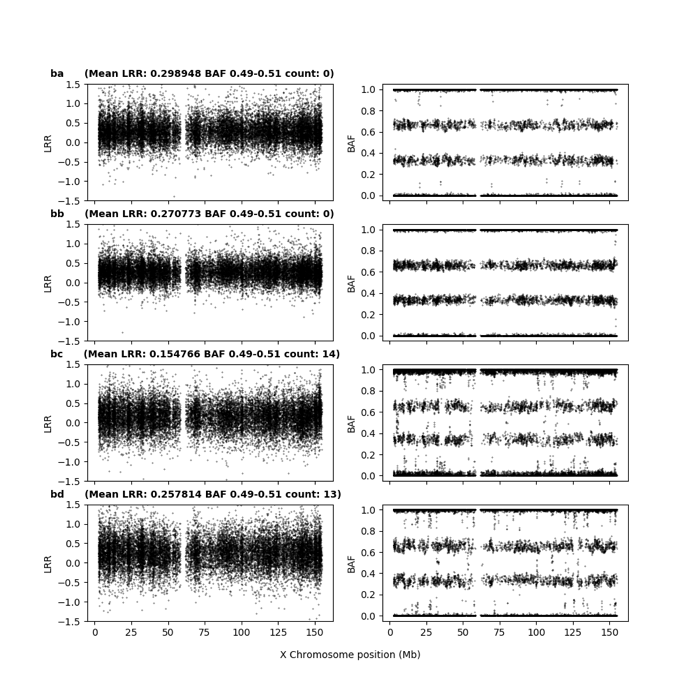

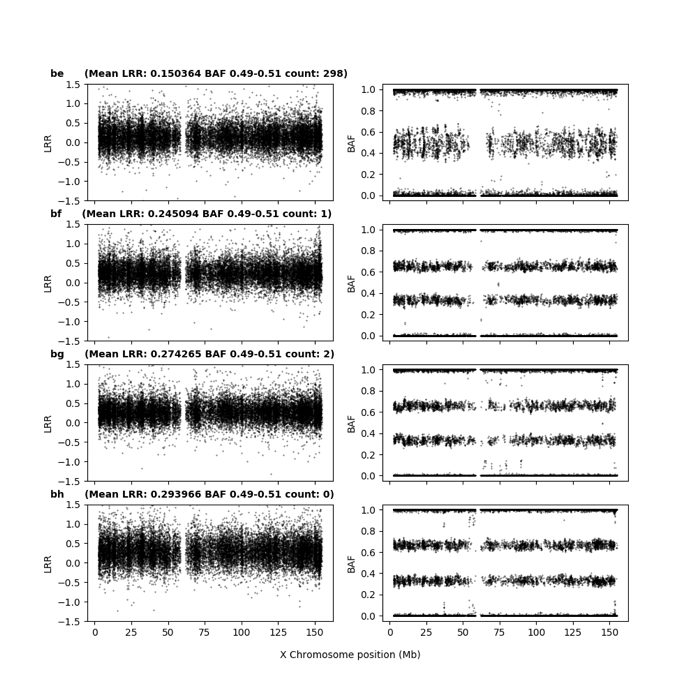

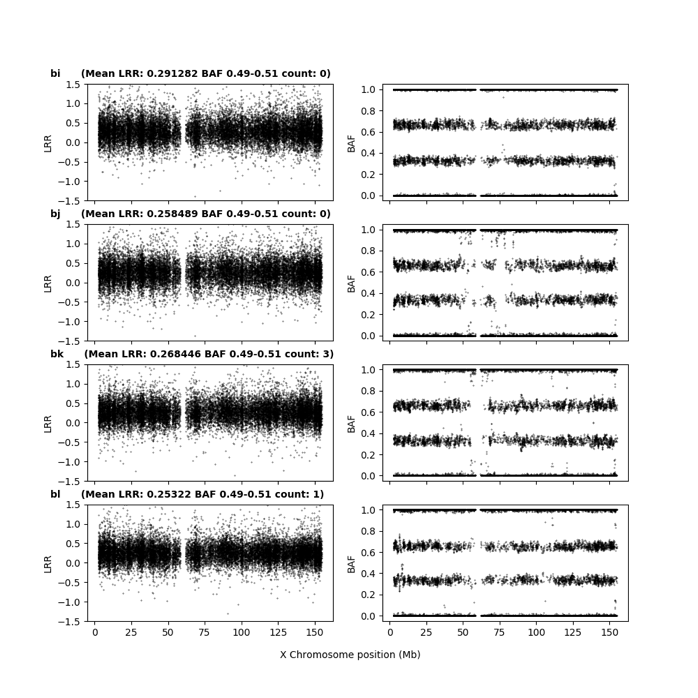

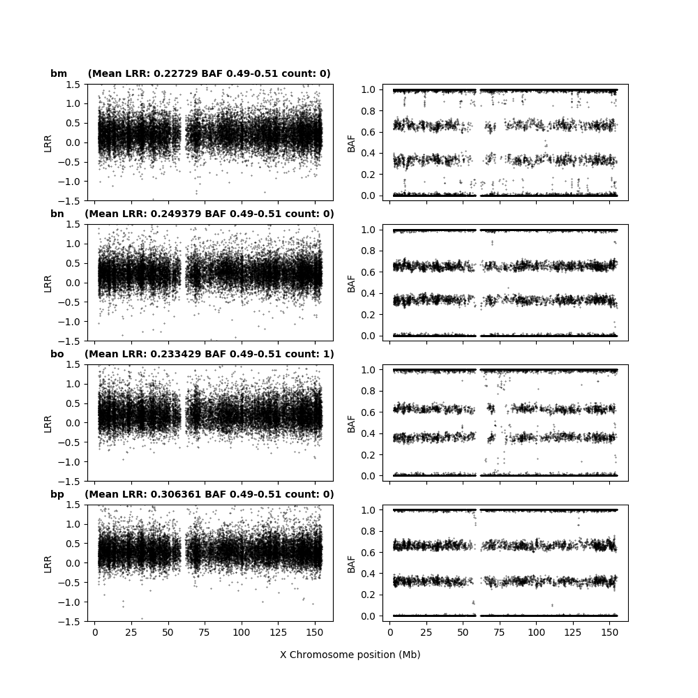

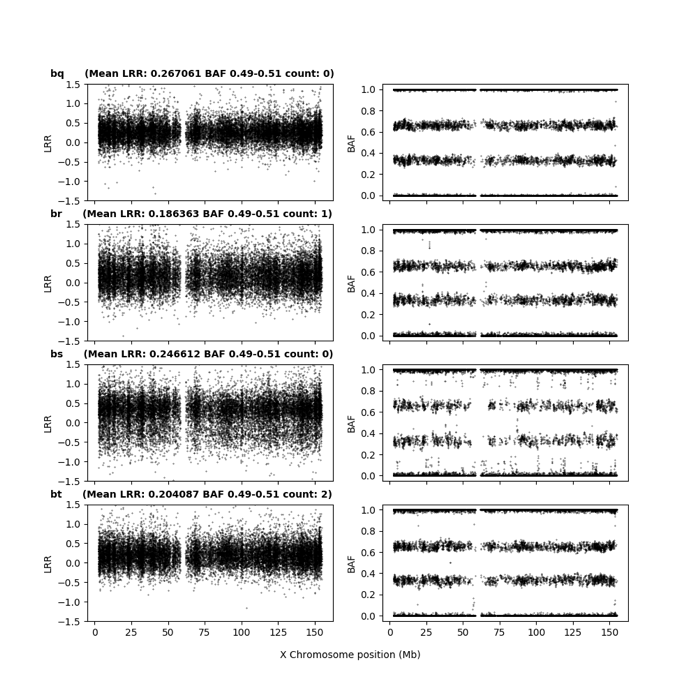

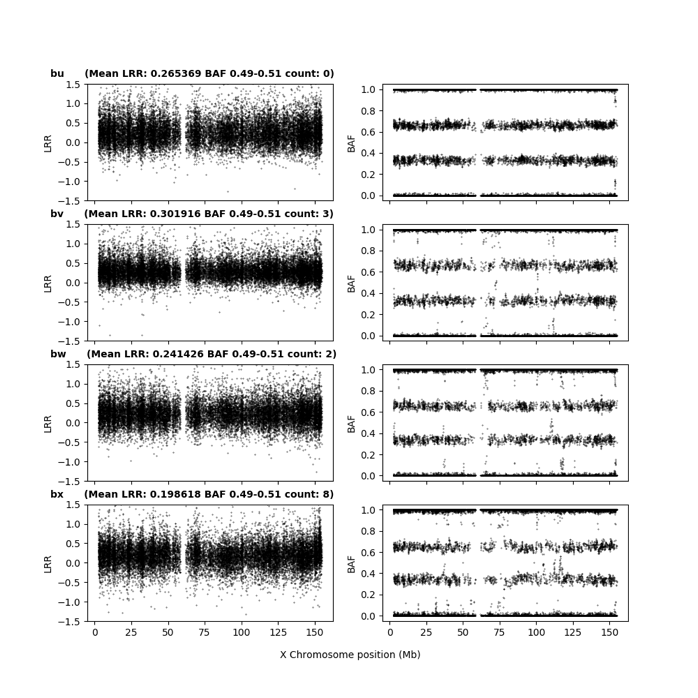

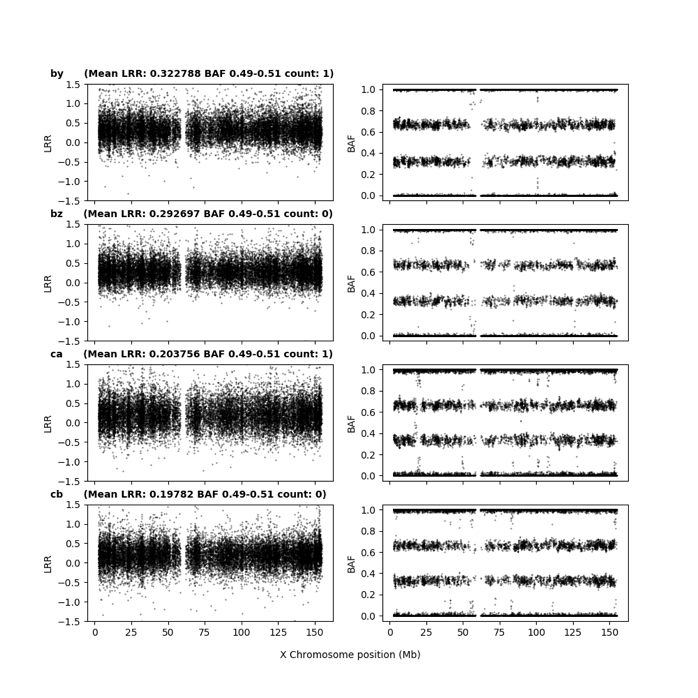

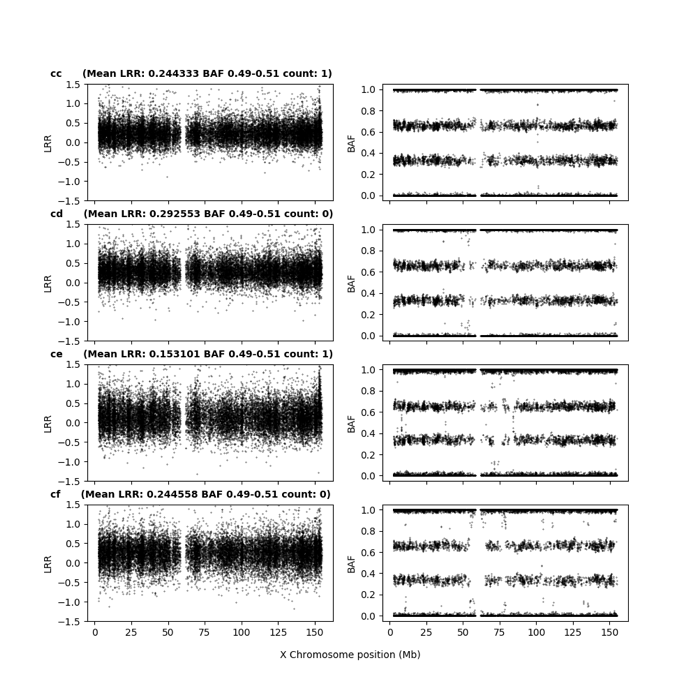

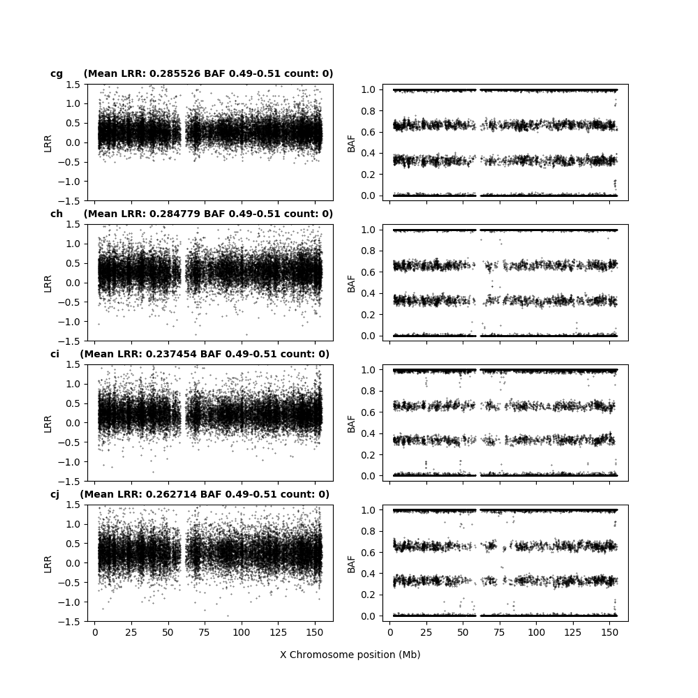

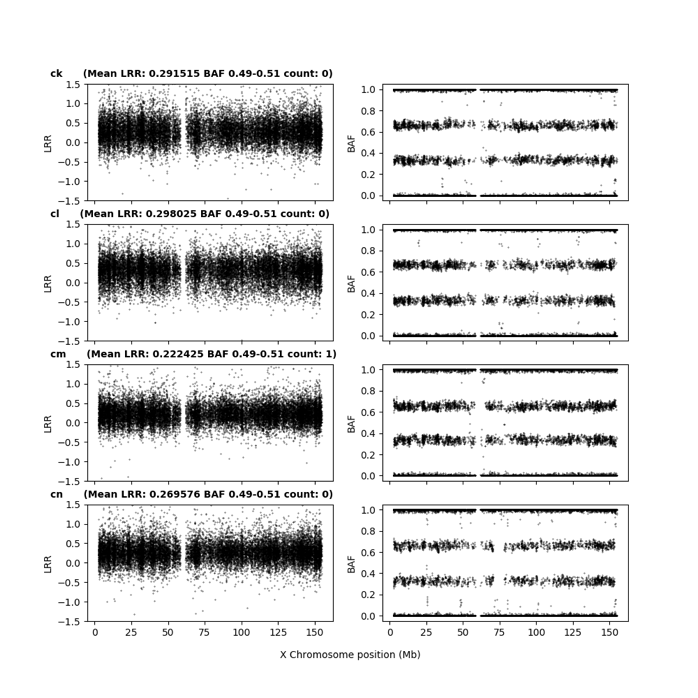

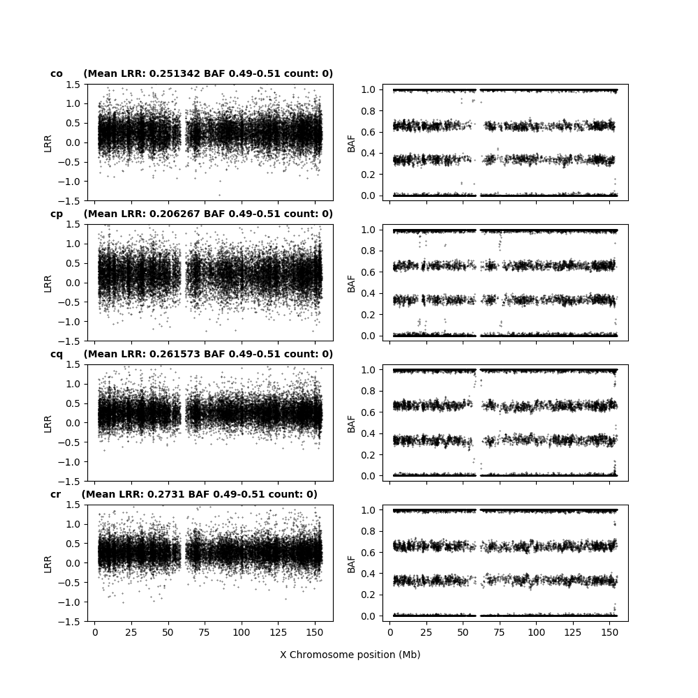

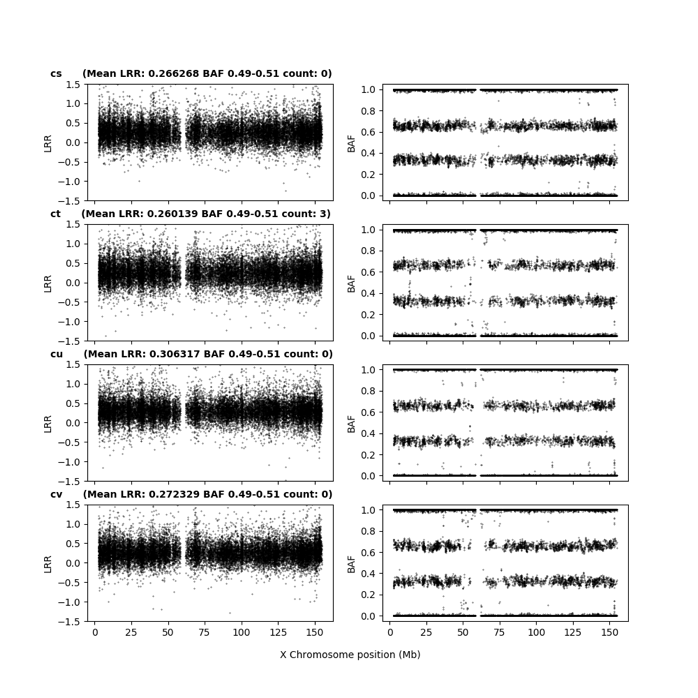

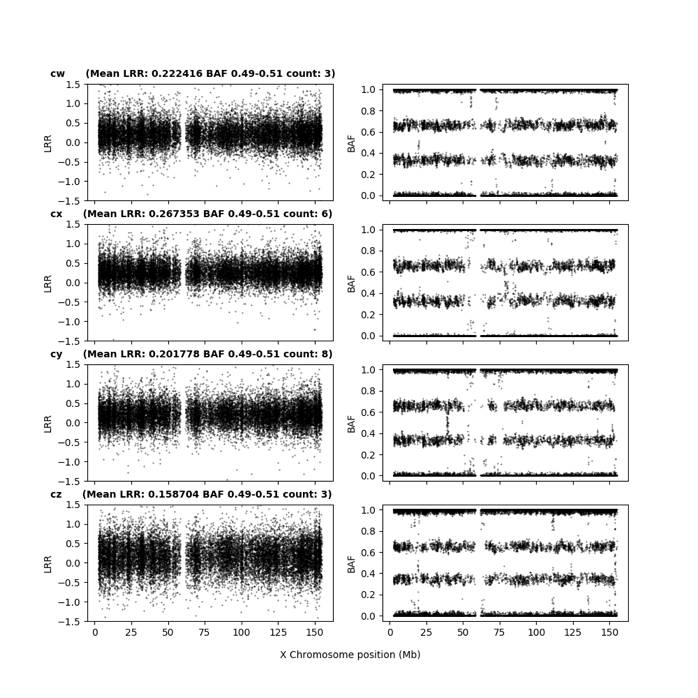

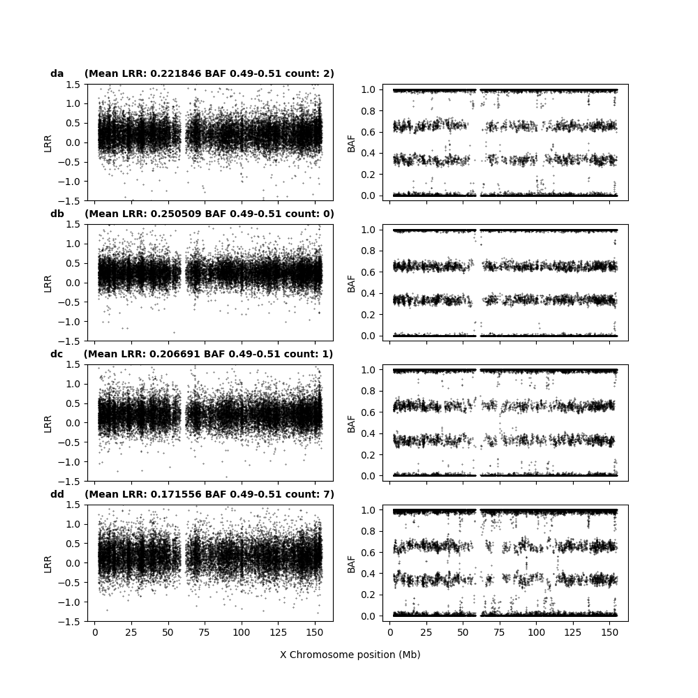

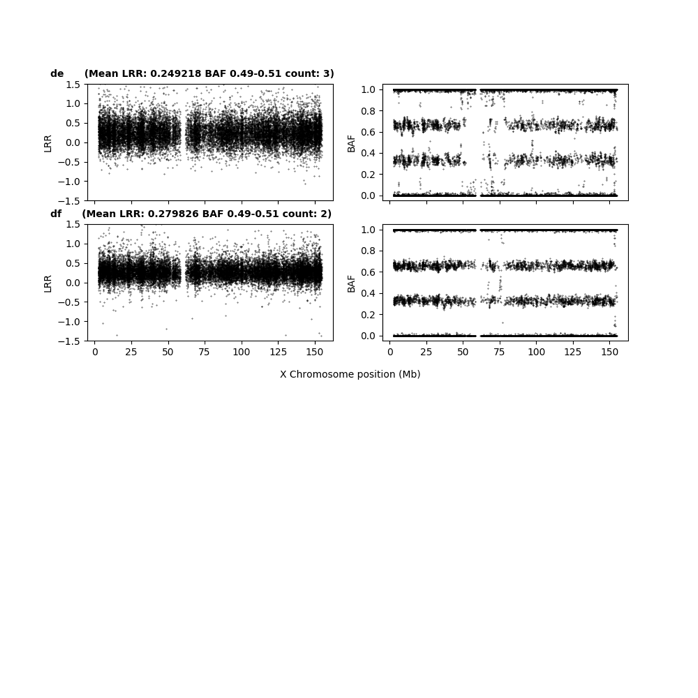
**

**Supplemental figure S5.** Log R Ratio (LRR) and B Allele Frequency (BAF) plots for each of the validation samples tested by conventional cytogenetics and compared with the SNP array method using two technologies: the Affymetrix UK Biobank Axiom array (**a, c, e, g, i and k**), and the Illumina Infinium HTS Global Screening array (**b, d, f, h, j and l**). Figures (**a**) and (**b**) represent LRR/BAF of a 45,X sample confirmed in both cytogenetics and both SNP arrays, (**c**) confirms the 11% 45,X/46,XX mosaicism in cytogenetic testing on the Axiom SNP array but appears 46,XX in the Illumina SNP array in (**d**), (**e**) and (**f**) represent LRR/BAF of a 20-25% 45,X/46,XX mosaic on both SNP arrays and was 26% 45,X/46,XX mosaic in cytogenetic testing, (**g**) and (**h**) were 46,XX in both SNP arrays confirming cytogenetic testing, (**i**) and (**j**) were 30% 45,X/46,XX on the SNP arrays but 13% 45,X/46,XX in cytogenetic testing, LRR suggested 67% mosaicism in (**k**), but BAF was not consistent across the whole X and would have been removed from our analysis, but (**i**) confirmed 50% 45,X/46,XX mosaicism in cytogenetic testing on the Illumina SNP array
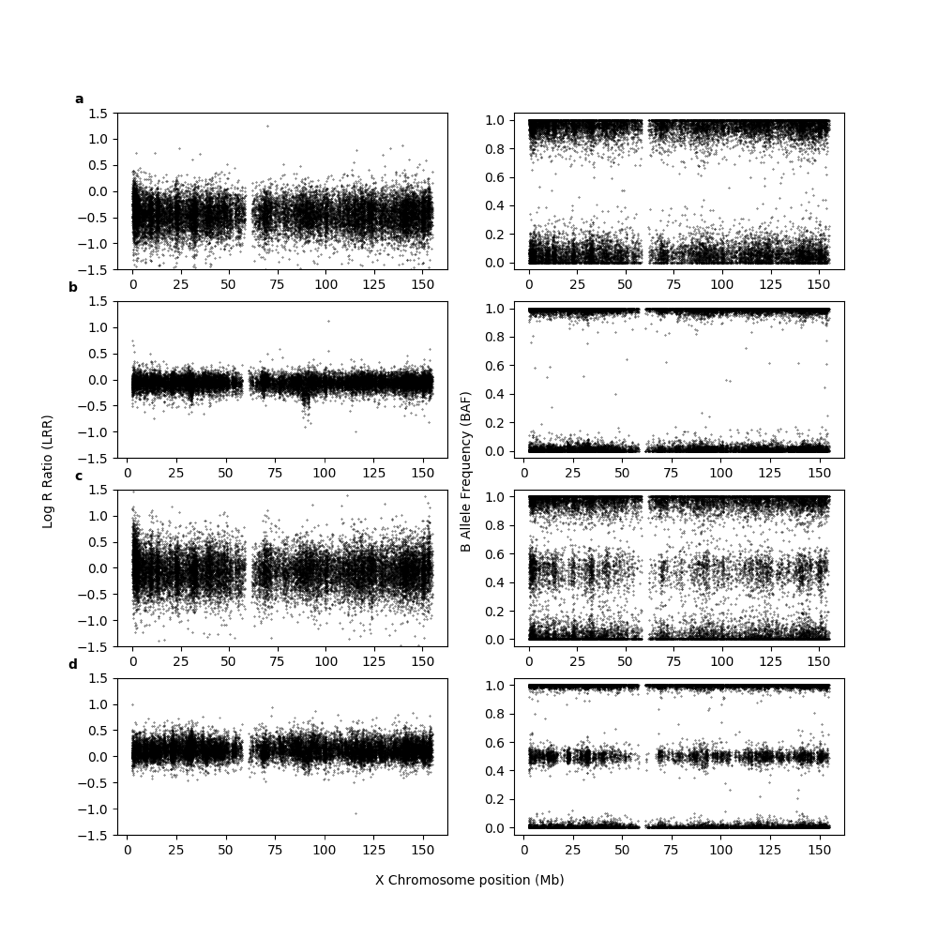


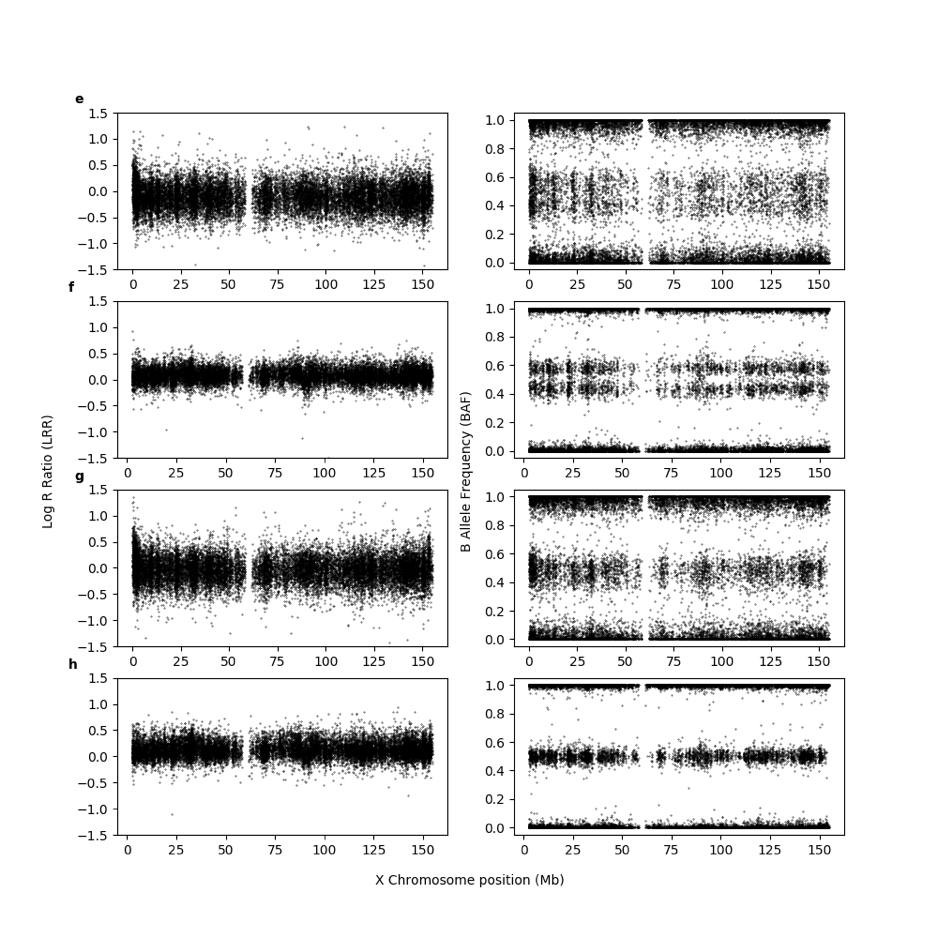

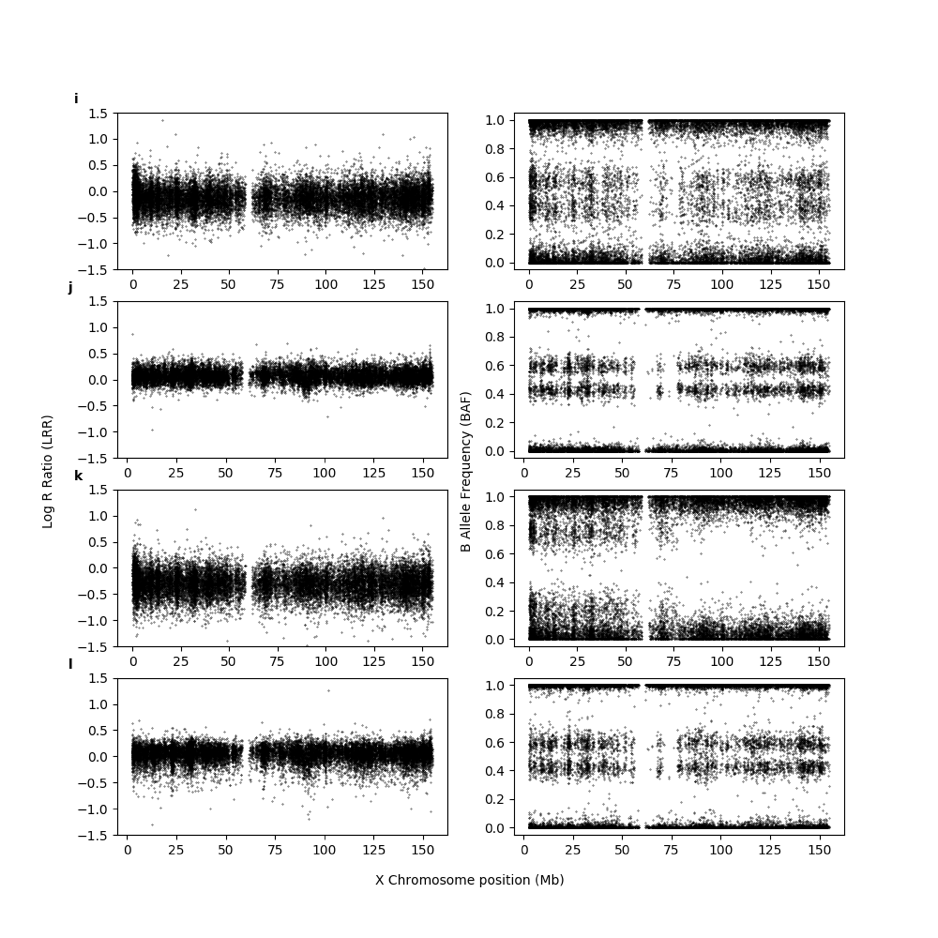


**Supplemental Figure S6.** The relationship between the 30 individuals detected as having full 45,X (indicated as blue circles) in our data and a total of 24 individuals with a ‘Q96 Turner Syndrome’ ICD-10 code (indicated as green circles). An explanation is given for the individuals with ICD-10 codes that were not identified as 45,X by our analyses

| - 5 with a normal 46,XX profile including   2 with a ’Q96.3 Mosaicism, 45,X/46,XX or XY’  diagnosis |
| --- |
| - 2 were Isochromosome Xq karyotype |

Individual with 45,X detected using SNP array that did not have a HES record

Individuals with a HES record that that include an ICD-10 code of ‘Q96.9 Turner Syndrome, unspecified’ but classified as a mosaic when using the SNP array

16

Total of 31 individuals with HES record that include an ICD-10 code of either ‘Q96.9 Turner Syndrome, unspecified’ (20), ‘Q96.9 Turner syndrome, unspecified’ (1) or ‘Q96.3 Mosaicism, 45,X/46,XX or XY’ (2)

29 Individuals with 45,X detected using SNP array that also had a HES record

7

13

**Supplemental Figure S7.** Plots (**a-g**) showing the remaining seven cases with a prior diagnosis of Turner syndrome not detected in our analysis. Two (**a and b**) were reported as ‘Turner mosaics’ but had a normal 46,XX SNP array profile, three (**c, e and g**) were reported as 45,X but had a normal 46,XX SNP array profile and two (**d and f**) had a deletion of the p-arm and duplication of the q-arm, suggesting isochromosome Xq
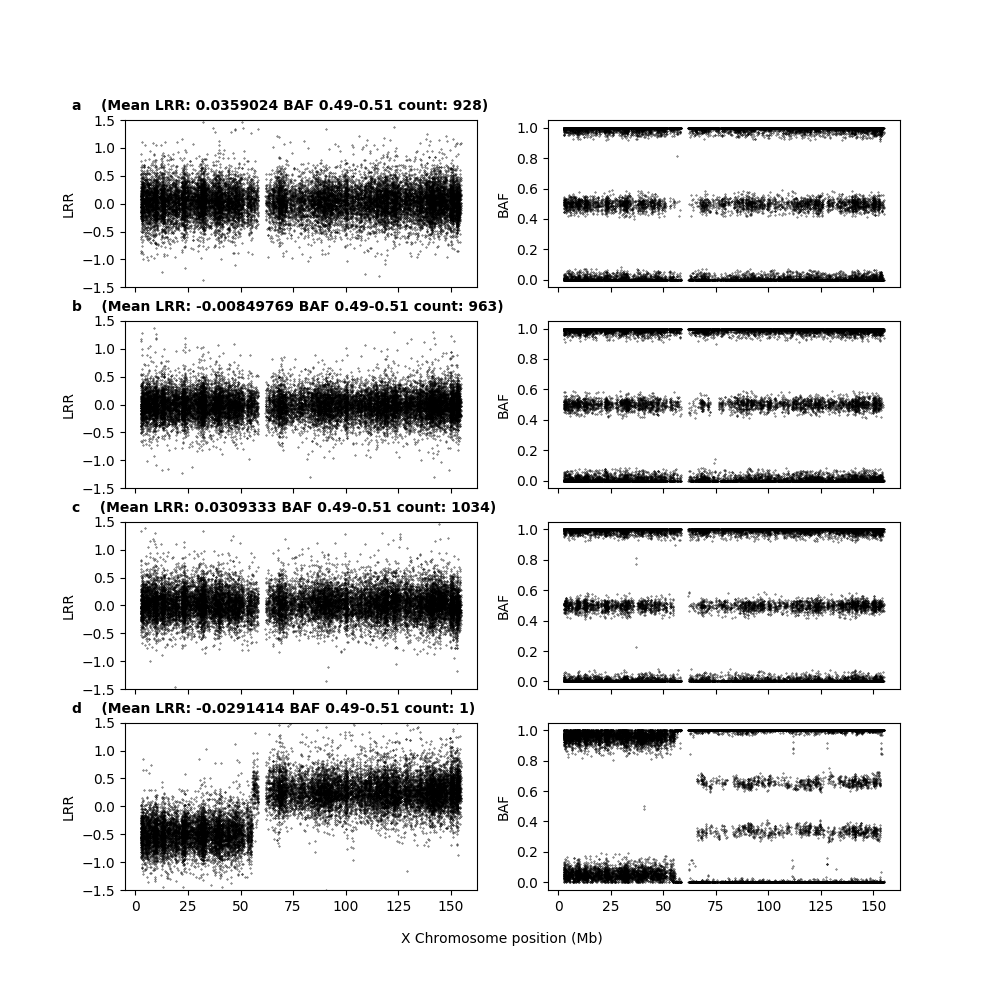


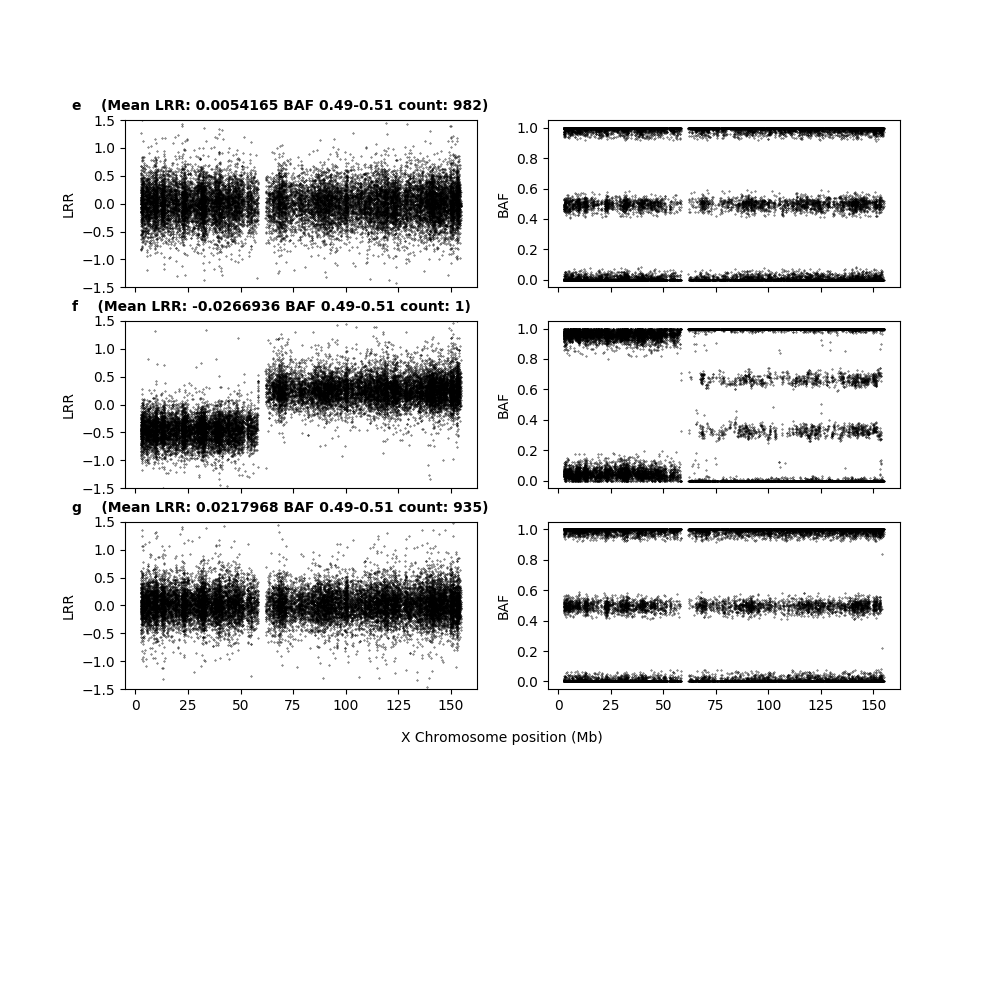


**Supplemental Figure S8.** Plots (**a-e**) represent Log R Ratio (LRR) and B Allele Frequency (BAF) in 5 individuals that were estimated as being 46,X,i(Xq) when analysing the Xp and Xq arms separately. In each case the karyotype is indicated by the presence of a single copy of Xp and three copies of Xq


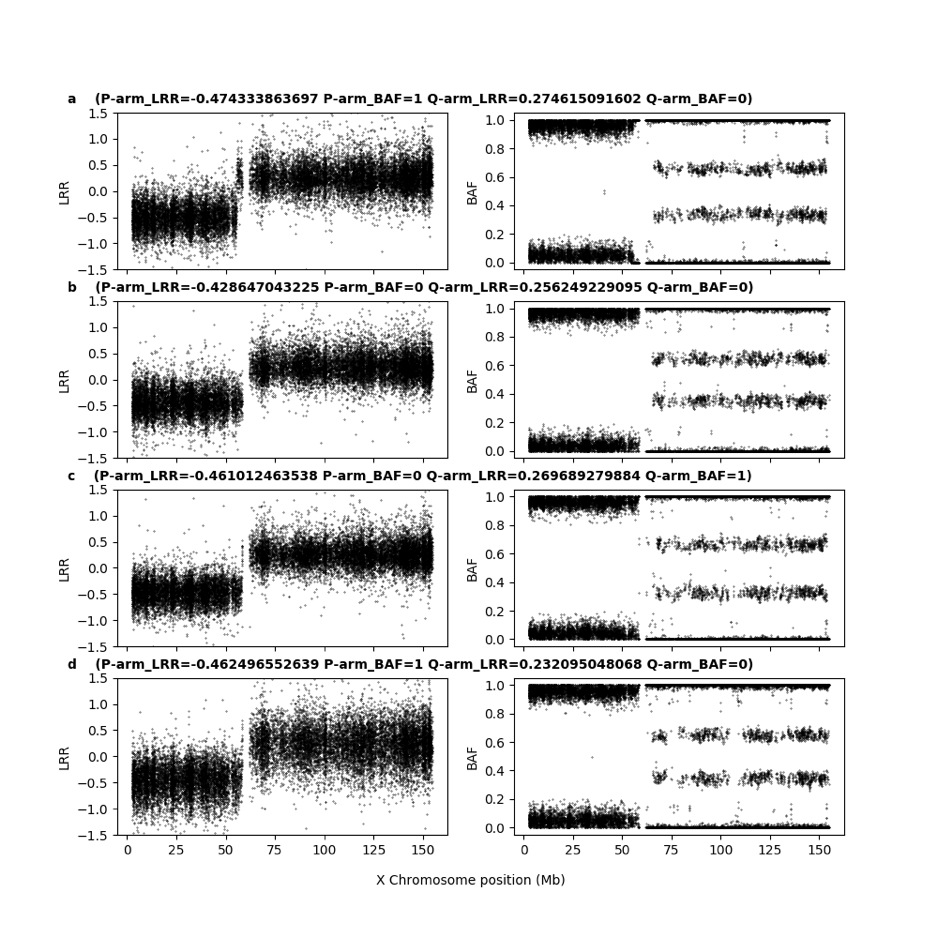


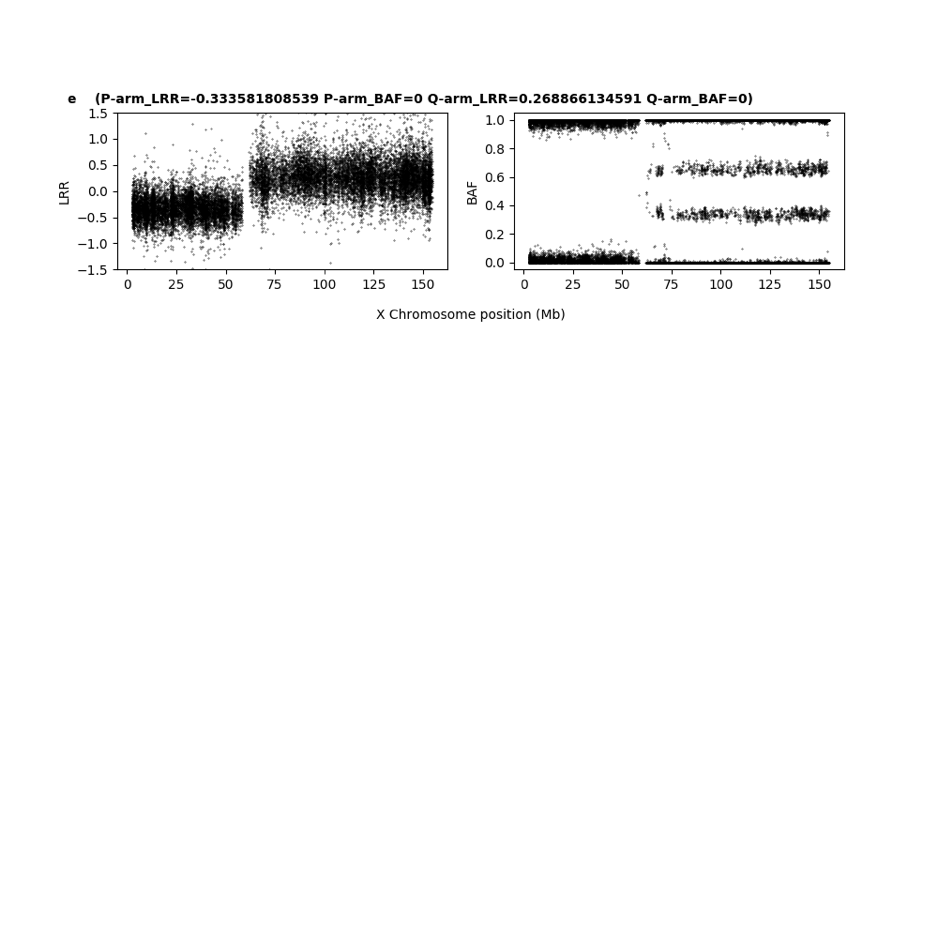


**Supplemental Figure S9.** Plots (**a-i**) represent Log R Ratio (LRR) and B Allele Frequency (BAF) in 9 individuals that had significantly large deletions when analysing Xp and Xq arms separately. The deletions range from 15Mb to 55Mb in size. Plot (**b**) is the only sample with a deletion of any part of Xq in this size range, all other deletions were on Xp
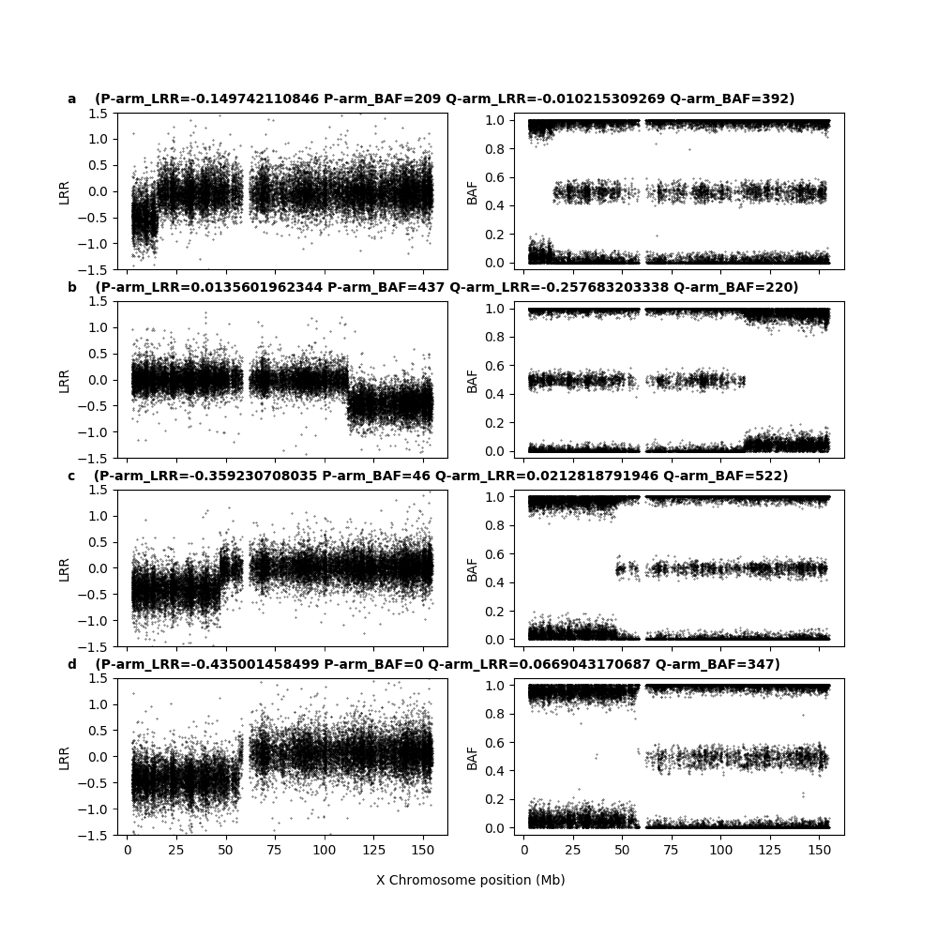


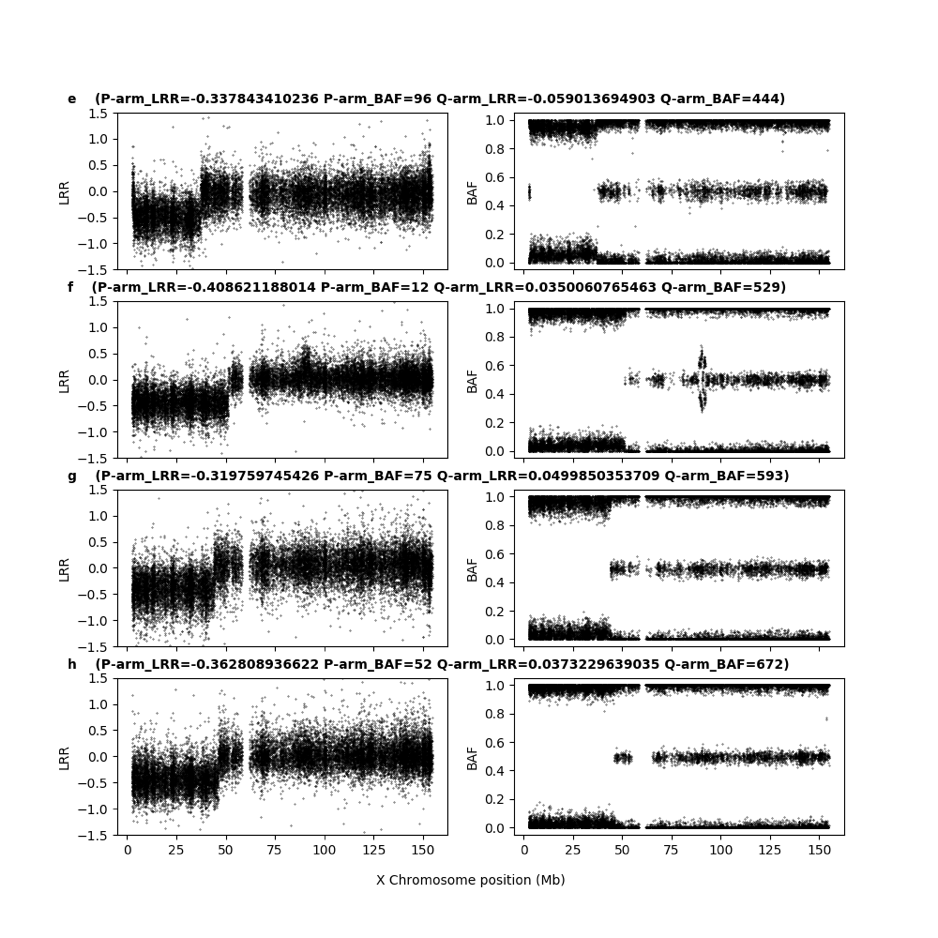

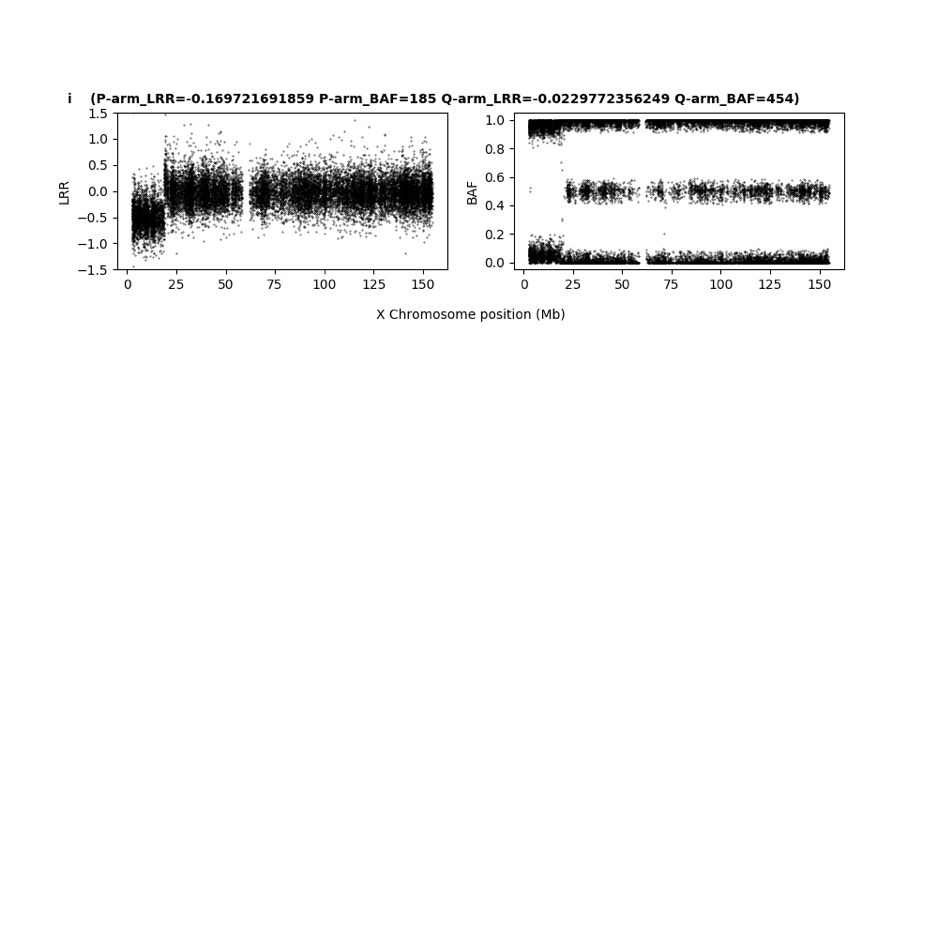


**Supplemental table S1.** Summary of demographic characteristics of 244,848 female participants of white European ancestry with valid genetic data. Characteristics are stratified by socioeconomic, anthropometric, cognitive, health and reproductive measures where available

| **Category** | **Trait** | **Count** | **Mean (SD)** | **Percentage** |
| --- | --- | --- | --- | --- |
| General | Age attended assessment centre (years) | 244,848 | 57.10 (7.94) |  |
| Socioeconomic status | Townsend Deprivation Index (TDI) | 244,564 | -1.5 (2.93) |  |
|  | Age full time education completed (years) | 166,708 | 16.62 (2.05) |  |
|  | Household income before tax: | 203,370 | 2.53 (1.18) |  |
|  | <£18 000 | 49,483 |  | 24.33 |
|  | £18 000 to £30 999 | 53,785 |  | 26.45 |
|  | £31 000 to £51 999 | 52,030 |  | 25.58 |
|  | £52 000 to £100 000 | 38,302 |  | 18.83 |
|  | >£100 000 | 9,770 |  | 4.8 |
| Anthropometric traits | Height (cm) | 244,363 | 162.65 (6.23) |  |
|  | Body mass index (BMI) | 243,827 | 27.02 (5.14) |  |
|  | Waist circumference (cm) | 244,415 | 84.55 (12.49) |  |
|  | Waist hip ratio | 244,377 | 0.82 (0.07) |  |
|  | Birthweight (kg) | 131,120 | 3.35 (0.42) |  |
|  | Whole body fat mass (kg) | 240,819 | 26.88 (9.99) |  |
|  | Sitting to standing height ratio | 244,113 | 0.53 (0.02) |  |
|  | Average arm fat percentage across both arms | 240,255 | 36.09 (8.91) |  |
|  | Average leg fat percentage across both legs | 240,291 | 40.30 (5.58) |  |
|  | Trunk fat percentage | 240,747 | 34.08 (7.78) |  |
|  | Trunk to leg ratio | 240,182 | 0.84 (0.12) |  |
|  | Trunk to limb ratio | 239,643 | 0.89 (0.10) |  |
| Cognitive ability | Total number incorrect matches in pairs memory test | 244,635 | 4.12 (3.21) |  |
|  | Fluid intelligence score | 118,917 | 5.78 (2.04) |  |
|  | Time to correctly identify matches (ms) | 243,273 | 564.26 (114.16) |  |
|  | Numeric memory test: max number of digits remembered | 76,605 | 6.77 (1.43) |  |
| Mental health | Happiness rating: | 80,123 | 2.56 (0.68) |  |
|  | Extremely happy | 3,998 |  | 4.99 |
|  | Very happy | 30,934 |  | 38.61 |
|  | Moderately happy | 41,913 |  | 52.31 |
|  | Moderately unhappy | 2,709 |  | 3.38 |
|  | Very unhappy | 449 |  | 0.56 |
|  | Extremely unhappy | 120 |  | 0.15 |
|  | Eysenck personality inventory (EPI) | 191,476 | 4.77 (3.25) |  |
|  | Tripartite model of subjective well-being (SWB) | 80,584 | -0.11 (1.01) |  |
|  | Number of depression episodes | 33,017 | 5.96 (35.86) |  |
| Reproductive traits | Menarche age (years) | 237,063 | 12.95 (1.56) |  |
|  | Age of natural menopause (years) | 108,639 | 50.02 (4.53) |  |
|  | Number pregnancies - includes live births, still births and miscarriages | 240,368 | 2.30 (1.58) |  |
|  | Number term pregnancies - includes live births and still births | 241,096 | 1.84 (1.20) |  |
|  | Number of live births | 244,694 | 1.81 (1.17) |  |
|  | Number of stillbirths | 241,194 | 0.03 (0.20) |  |
|  | Number of miscarriages | 241,014 | 0.30 (0.74) |  |
|  | Number of pregnancy terminations | 240,759 | 0.16 (0.46) |  |
|  | Mean length in days of menstrual cycle (days) | 35,499 | 26.56 (3.58) |  |
| Physical health | Overall health rating | 243,965 | 2.08 (0.71) |  |
|  | Excellent | 42,783 |  | 17.54 |
|  | Good | 146,560 |  | 60.07 |
|  | Fair | 45,729 |  | 18.74 |
|  | Poor | 8,893 |  | 3.65 |
|  | Number of treatments/medications reported | 244,819 | 2.56 (2.66) |  |
|  | Number of operations reported | 244,819 | 2.01 (1.69) |  |
|  | Number of cancers reported | 244,819 | 0.11 (0.34) |  |
|  | Number of non cancer illnesses reported | 244,819 | 1.90 (1.93) |  |
|  | Left ear Speech Reception Threshold (SNR) | 76,991 | -6.71 (1.90) |  |
|  | Right ear Speech Reception Threshold (SNR) | 76,922 | -6.67 (1.92) |  |
|  | Bone mineral density (g/cm2) | 240,382 | 0.52 (0.12) |  |
|  | Pulse rate (bpm) | 229,939 | 70.18 (10.61) |  |
|  | Pulse wave Arterial Stiffness index | 79,006 | 8.81 (3.93) |  |
|  | Maximum hand grip strength | 243,021 | 22.25 (6.53) |  |

**Supplemental Table S2.** Association of 46,X,i(Xq) and 46,XX,del(X) with inverse normalised height

| **Trait** | **Status** | **N** | **Effect (SD)** | **Low 95% CI (SD)** | **High 95% CI (SD)** | ***P*** |
| --- | --- | --- | --- | --- | --- | --- |
| Height (inverse normalised) | 46,XX | 244,508 |  |  |  |  |
|  | 46,X,i(Xq) | 5 | -2.54 | -3.16 | -1.92 | 6.70E-16 |
|  | 46,XX,del(X) | 9 | -1.55 | -2.01 | -1.09 | 4.20E-11 |

**Supplemental Table S3.** Association between X chromosome ploidy and nine untransformed phenotypes. The effect of the aneuploidy on each trait is given in SD units compared with 46,XX women

| Trait | Status | N | Effect | Low 95% CI | High 95% CI | *P* |
| --- | --- | --- | --- | --- | --- | --- |
| Height (cm) | 45,X (all) | 216 | -5.35 | -6.15 | -4.54 | 1.4E-38 |
|  | 45,X (> 80%) | 30 | -17.64 | -19.81 | -15.48 | 1.6E-57 |
|  | 45,X (<80%) | 186 | -3.36 | -4.23 | -2.49 | 3.3E-14 |
|  | 47,XXX | 110 | 5.32 | 4.19 | 6.45 | 2.9E-20 |
| Menarche Age (Years) | 45,X (all) | 191 | 0.26 | 0.03 | 0.48 | 2.4E-02 |
|  | 45,X (> 80%) | 14 | 1.14 | 0.18 | 2.11 | 2.1E-02 |
|  | 45,X (<80%) | 177 | 0.21 | -0.02 | 0.44 | 7.6E-02 |
|  | 47,XXX | 104 | -0.15 | -0.45 | 0.16 | 3.4E-01 |
| Natural Menopause Age | 45,X (all) | 100 | 0.08 | -0.79 | 0.96 | 8.5E-01 |
| (Years) | 45,X (> 80%) | 5 | -4.80 | -8.71 | -0.88 | 1.6E-02 |
|  | 45,X (<80%) | 95 | 0.34 | -0.56 | 1.24 | 4.6E-01 |
|  | 47,XXX | 60 | -4.86 | -5.99 | -3.73 | 3.9E-17 |
| Number of | 45,X (all) | 215 | -0.39 | -0.60 | -0.18 | 2.5E-04 |
| Pregnancies | 45,X (> 80%) | 29 | -2.04 | -2.61 | -1.47 | 2.5E-12 |
|  | 45,X (<80%) | 186 | -0.14 | -0.36 | 0.09 | 2.4E-01 |
|  | 47,XXX | 107 | -0.37 | -0.67 | -0.07 | 1.5E-02 |
| Fluid Intelligence Score | 45,X (all) | 90 | 0.17 | -0.24 | 0.59 | 4.2E-01 |
| (0-13) | 45,X (> 80%) | 11 | -0.14 | -1.33 | 1.04 | 8.1E-01 |
|  | 45,X (<80%) | 79 | 0.22 | -0.23 | 0.66 | 3.4E-01 |
|  | 47,XXX | 51 | -1.53 | -2.08 | -0.98 | 5.4E-08 |
| Household Income | 45,X (all) | 177 | -0.16 | -0.32 | 0.00 | 5.2E-02 |
| Category (1-5) | 45,X (> 80%) | 21 | -0.47 | -0.93 | -0.01 | 4.7E-02 |
|  | 45,X (<80%) | 156 | -0.12 | -0.28 | 0.05 | 1.8E-01 |
|  | 47,XXX | 78 | -1.04 | -1.27 | -0.80 | 1.9E-17 |
| Townsend Deprivation Index | 45,X (all) | 216 | 0.34 | -0.03 | 0.70 | 6.9E-02 |
|  | 45,X (> 80%) | 30 | 0.26 | -0.71 | 1.24 | 6.0E-01 |
|  | 45,X (<80%) | 186 | 0.35 | -0.04 | 0.74 | 8.1E-02 |
|  | 47,XXX | 110 | 0.83 | 0.32 | 1.35 | 1.3E-03 |
| Birthweight (kg) | 45,X (all) | 103 | -0.16 | -0.26 | -0.05 | 3.4E-03 |
|  | 45,X (> 80%) | 13 | -0.47 | -0.74 | -0.21 | 4.2E-04 |
|  | 45,X (<80%) | 89 | -0.10 | -0.21 | 0.02 | 9.9E-02 |
|  | 47,XXX | 56 | -0.18 | -0.33 | -0.03 | 1.7E-02 |
| Body Mass Index (kgm^2^) | 45,X (all) | 215 | -0.32 | -1.00 | 0.37 | 3.6E-01 |
|  | 45,X (> 80%) | 30 | 0.33 | -1.50 | 2.16 | 7.2E-01 |
|  | 45,X (<80%) | 185 | -0.42 | -1.16 | 0.31 | 2.6E-01 |
|  | 47,XXX | 110 | 2.14 | 1.18 | 3.09 | 1.2E-05 |

**Supplemental Table S4.** Degree of X chromosome loss in samples tested by conventional cytogenetics, compared to SNP arrays

| **Sample** | **Cytogenetic estimate** | **Illumina array estimate** | **Affymetrix array estimate** |
| --- | --- | --- | --- |
| 1 | 100% 45,X | 100% 45,X (**Fig. S1a**) | 100% 45,X (**Fig. S1b**) |
| 2 | 11% 45,X* | 2% 45,X (**Fig. S1c**) | 13% 45,X (**Fig. S1d**) |
| 3 | 26% 45,X | 25% 45,X (**Fig. S1e**) | 23% 45,X (**Fig. S1f**) |
| 4 | 46,XX | 46,XX (**Fig. S1g**) | 46,XX (**Fig. S1h**) |
| 5 | 24% 45,X | 29% 45,X (**Fig. S1i**) | 30% 45,X (**Fig. S1j**) |
| 6 | 50% 45,X | 48% 45,X (**Fig. S1k**) | 67% 45,X^+^ (**Fig. S1l**) |

* Cytogenetics report stated that this could be due to age-related loss of the X, this was the only control sample from a patient possibly old enough to demonstrate age-related loss (34 years old) and was referred in her mid-thirties for infertility. Both SNP arrays estimated a low level of mosaicism consistent with the cytogenetic estimate, but the LRRs and BAFs were not sufficiently outside the normal range for a sample such as this to be included in our case series. Sample 2

+ While the LRR was consistent with the cytogenetic estimate of % mosaicism, the BAF plot was inconsistent across the X chromosome in the Affymetrix data and would have been excluded from our case series, as we selected only individuals with evidence of whole X chromosome imbalance (**Supplemental Fig. 5k**).
